# Supplementary material for: Common mental health and emotional and behavioural disorders among adolescents and young adults in Harare and Mashonaland East, Zimbabwe: a population-based prevalence study
Source: BMJ Open. 2023 Mar 14;13(3):e065276. doi: 10.1136/bmjopen-2022-065276 (PMC10016291; doi:10.1136/bmjopen-2022-065276)
Supplement: Supplementary data [file bmjopen-2022-065276supp001.pdf]

Supplementary Material

Questionnaire (YTM Q02 n6)

Shona	English
**SECTION 0 RECRUITMENT INFORMATION**	**SECTION 0 RECRUITMENT INFORMATION**
[Q01] Date of Interview	[Q01] Date of Interview
[Q02] Whats the Suburb Name?	[Q02] Whats the Suburb Name?
[Q022] Whats the Suburb Name?	[Q022] Whats the Suburb Name?
[Q03] Cluster ID?	[Q03] Cluster ID?
[Q032] Cluster ID?	[Q032] Cluster ID?
[Q04] Dwell ID?	[Q04] Dwell ID?
[Q042] Dwell ID?	[Q042] Dwell ID?
[Q05] Household ID?	[Q05] Household ID?
[Q05m] Manually enter the Household ID?	[Q05m] Manually enter the Household ID?
[Q05m] Manually enter the Household ID?	[Q05m] Manually enter the Household ID?
[Q06] Respondent No?	[Q06] Respondent No?
[Q062] Respondent No?	[Q062] Respondent No?
[Q07] Select your name	[Q07] Select your name
[Q08] Please select the language for this interview	[Q08] Please select the language for this interview
[Q101] Mukomana kana musikana here?	[Q101] Respondent Sex
[Q102] Wave nemakore mangani?	[Q102] How old were you at your last birthday?
[Q103] Panguva ino wakamira sei panyaya dzewanano?	[Q103] What is your current marital status?
[Q104] Wakambopinda chikoro here?	[Q104] Have you ever attended school?
[Q106] Kuchikoro wakasvika pai: primary, secondary, kana pamusoro?	[Q106] What is the highest level of school you attended: primary, secondary, or higher?
[Q105] Ndezvipi zvinonyatsotsanangura zvauri kuita mazuvano?	[Q105] Which of the following best describes your current occupational status?
[Q107] Uri vechitendero chipi?	[Q107] What is your religion?
[Q107] Tsanangudza chimwe chitendero?	[Q107] Specify other religion?
[Q108] Unowanzotaura mutauro upi kana usiri kumba?	[Q108] Outside your home, what language do you speak the most?
[Q108] Tsanangudza mumwe mutauro waunotaura usiri pamba?	[Q108] Specify other language?
**NGUVA YAWAGARA PAMBA PANO KANA MUNHARAUNDA INO**	**DURATION OF STAY AT RESIDENCE**
[Q109] Wave nenguva yakareba zvakadii uchigara munharaunda ino?	[Q109] How long have you lived in this community?
[Q110] Mumwedzi gumi nemiviri yapfuura, wakambofamba wakanogara kuneimwe nzvimbo kwemwedzi kana kupfuura here?	[Q110] In the last 12 months, have you travelled and stayed in a place other than this community for one month or more?
[Q111] Kusanganisa nguva yese yawakagara kunze kwenharaunda ino, ungati wakagara nguva yakadii mumwedzi negumi nemiviri yapfuura?	[Q111] In total approximately how long have you spent outside this community in the last 12 months?
**MHURI**	**FAMILY**

[Q112] Baba vako vakakubereka vapenyu here?	[Q112] Is your natural father alive?
[Q113] Mai vako vakakubereka vapenyu here?	[Q113] Is your natural mother alive?
Chimbonditaurira nezvinhu zve MICHINA (TECHNOLOGY) zvamunazvo mumba menyu	Please tell us about some technological devices- which of the following do you have at home
**MICHINA (TECHNOLOGY) IRI MUMBA**	**TECHNOLOGY AT HOME**
[Q201h] Normal/'desktop' computer	[Q201h] Normal/'desktop' computer
[Q201h] Laptop computer (including notebooks and netbooks)	[Q201h] Laptop computer (including notebooks and netbooks)
[Q201h] Tablet/ iPad	[Q201h] Tablet/ iPad
[Q201h] Feature (non-smart) Cell phone	[Q201h] Feature (non-smart) Cell phone
[Q201h] Basic feature (calls and sms only) Cellphone	[Q201h] Basic feature (calls and sms only) Cellphone
[Q201h] Smart cell phone	[Q201h] Smart cell phone
[Q201h6] Ipod or other MP3 player	[Q201h6] Ipod or other MP3 player
[Q201h] TV	[Q201h] TV
[Q201h] Radio	[Q201h] Radio
[Q201h] Digital camera	[Q201h] Digital camera
[Q201h] Gaming console (Playstation, Xbox, Nintendo wii etc.)	[Q201h] Gaming console (Playstation, Xbox, Nintendo wii etc.)
[Q201h] Handheld gaming device (e.g. DS, PSP, …)	[Q201h] Handheld gaming device (e.g. DS, PSP, …)
**UNOSHANDISA MICHINA(TECHNOLOGY) IPI**	**USE OF TECHNOLOGY**
[Q201u] Normal/'desktop' computer	[Q201u] Normal/'desktop' computer
[Q201u] Laptop computer (including notebooks and netbooks)	[Q201u] Laptop computer (including notebooks and netbooks)
[Q201u] Tablet/ iPad	[Q201u] Tablet/ iPad
[Q201u] Feature (non-smart) Cell phone	[Q201u] Feature (non-smart) Cell phone
[Q201u] Basic feature (calls and sms only) Cellphone	[Q201u] Basic feature (calls and sms only) Cellphone
[Q201u] Smart cell phone	[Q201u] Smart cell phone
[Q201u6] Ipod or other MP3 player	[Q201u6] Ipod or other MP3 player
[Q201u] TV	[Q201u] TV
[Q201u] Radio	[Q201u] Radio
[Q201u] Digital camera	[Q201u] Digital camera
[Q201u] Gaming console (Playstation, Xbox, Nintendo wii etc.)	[Q201u] Gaming console (Playstation, Xbox, Nintendo wii etc.)
[Q201u] Handheld gaming device (e.g. DS, PSP, …)	[Q201u] Handheld gaming device (e.g. DS, PSP, …)
[Q202] Wakamboshandisa internet here?	[Q202] Have you ever used the internet??
**KUSHANDISA INTERNET**	**USE OF INTERNET**
[Q203] Kana wakashandisa, wakapedzisira kushandisa internet rinhi?	[Q203] If yes, when is the last time that you used the internet?
Bvunza kuti anoshandisa internet kupi, nguva yakadini	Ask about when the participants uses the internet, how often do they do so
**Unoshandisa Internet nguva yakadini**	**How ofte do you use internet**
[Q204]Pacomputer kuchikoro kana kubasa	[Q204]On a computer at school/ work
[Q204]Pacomputer kumba	[Q204]On a computer at home
[Q204]Pacell/mobile phone	[Q204]On a cell/mobile phone
[Q204]Pacomputer kulibrary/ kana community internet shop	[Q204]At a computer in the library/ other community internet facility
[Q204]Ku internet café inobhadharwa	[Q204]At a commercial internet café- where you pay for the access
[Q204]Pacomputer kumba kwevamwewo	[Q204]At a computer in someone else's house
[Q205] Unopedza nguva yakadini uri painternet?	[Q205] On average how much time do you spend on the internet when you go online?
[Q206] Chii chaunofarira kunyanya painternet?	[Q206] What do you like the most about the internet?

[Q206] Tsanangudza chimwe chikonzero chaunofarira painternet?	[Q206] Specify other reason what you like about the internet?
[Q207] Chii chausinga farire kunyaya painternet?	[Q207] What do you dislike the most about the internet?
[Q207] Tsanangudza chimwe chikonzero chausingafarire painternet?	[Q207] Specify other reason what you dislike about the internet?
[Q208] Chii chaungade kuti chishanduke kuti kushandisa kwako internet kuve nani?	[Q208] What one change would you like to see so that use of the internet could be better for you?
[Q208] Tsanangudza chimwe chinhu chaungade kuti chishanduke?	[Q208] Specify other change you would like to see?
[Q209] Une cell phone here kana kuti unoshandisa cell phone yaunokumbidzana kana kushandisa nevamwe?	[Q209] Do you have or share mobile (cell) phone)?
**VANE MOBILE PHONE KANA VANOSHANDISA**	**OWNERSHIP AND USE OF MOBILE PHONE**
[Q209a] Kana ati hongu; Une cell phone ngani dzako pachako?	[Q209a] If Yes, how many phones you own by yourself ?
[Q209b] Kana ati hongu; Une cell phone ngani dzaunokumbidzana kushandisa nevamwe?	[Q209b] If Yes, how many phones do share with someone else?
[Q210] Une maline mangani e cell phone aunokushandisa parizvino?	[Q210] How many mobile phone numbers do you currently use?
[Q211] Mugore rapfuura, maline mangani e cell phone yawakanga unayo kusanganisira aunawo parizvino?	[Q211] How many different mobile phone numbers have you had in the past year including your current number (s)?
Ngatimbotaura nema cell phone ${Q209amob} awunawo	Lets talk about the ${Q209amob} mobile phones you own
**MHANDO DZEMAMOBILE PHONE DZAANADZO**	**MOBILE PHONE TYPES OWNED**
**Phone Yokutanga Yaanayo**	**Phone 1 Owned**
[Q212O]Foni yekutanga yaunayo inonzii?	[Q212O]What is the make of the first phone you own?
[Q212O]Model yefoni yekutanga chii?	[Q212O]What is the model of the first phone you own?
[Q212O] Foni yekutanga iyi smart phone here?	[Q212O] Is the first phone you own a smart phone?
[Q212O] Foni yekutanga iyi wakaitenga gore ripi?	[Q212O] Which year was the first phone you own purchased?
**Phone Yechipiri Yaanayo**	**Phone 2 Owned**
[Q212O]Foni yechipiri yaunayo inonzii?	[Q212O]What is the make of the second phone you own?
[Q212O]Model yefoni yechipiri chii?	[Q212O]What is the model of the second phone you own?
[Q212O] Foni yechipiri iyi smart phone here?	[Q212O] Is the second phone you own a smart phone?
[Q212O] Foni yechipiri iyi wakaitenga gore ripi?	[Q212O]Which year was the second phone you own purchased?
**Phone Yechitatu Yaanayo**	**Phone 3 Owned**
[Q212O]Foni yechitatu yaunayo inonzii?	[Q212O]What is the make of the third phone you own?
[Q212O]Model yefoni yechitatu chii?	[Q212O]What is the model of the third phone you own?
[Q212O] Foni yechitatu iyi smart phone here?	[Q212O]Is the third phone you own a smart phone?
[Q212O] Foni yechitatu iyi wakaitenga gore ripi?	[Q212O]Which year was the third phone you own purchased?
Nhatimbotaurai nezvema cell phone ${Q209bmob} aunokumbidzana nevamwe	Lets talk about the ${Q209bmob} mobile phones you share
**MOBILE PHONE TYPES SHARED**	**MOBILE PHONE TYPES SHARED**

**Phone Yokutanga Yaanokumbidzana kana kushandisa nevamwe**	**Phone 1 Shared**
[Q212S]Foni yekutanga yaunokumbidzana nevamwe inonzii?	[Q212S]What is the make of the first phone you share?
[Q212S]Model yefoni yekutanga chii?	[Q212S]What is the model of the first phone you share?
[Q212S] Foni yekutanga iyi smart phone here?	[Q212S] Is the first phone you share a smart phone?
[Q212S] Foni yekutanga iyi wakaitenga rinhi?	[Q212S] Which year was the first phone you share purchased?
[Q213S] Unoshandisa kana kukumbidzana cell phone yokutanga nani?	[Q213S] Who do you share ownership of your first phone with?
[Q213S] Tsanangudza vamwe vanhu vaunoshandisa phone navo	[Q213S] Specify other
[Q213S] Kakawanda zvakadii kamunowana mukana wekushandisa phone yokutanga?	[Q232S] How often do you have access to the first phone you share?
**Phone Yechipiri Yaanokumbidzana kana kushandisa nevamwe**	**Phone 2 Shared**
[Q212S]Foni yechipiri yaunokumbidzana nevamwe inonzii?	[Q212S]What is the make of the second phone you share?
[Q212S]Model yefoni yechipiri chii?	[Q212S]What is the model of the second phone you share?
[Q212S] Foni yechipiri iyi smart phone here?	[Q212S] Is the second phone you share a smart phone?
[Q212S] Foni yechipiri iyi wakaitenga rinhi?	[Q212S]Which year was the second phone you share purchased?
[Q213S] Unoshandisa kana kukumbidzana cell phone yechipiri nani?	[Q213S] Who do you share ownership of your second phone with?
[Q213S] Tsanangudza vamwe vanhu vaunoshandisa phone navo	[Q213C] Specify other
[Q213S] Kakawanda zvakadii kamunowana mukana wekushandisa phone yechipiri?	[Q232S] How often do you have access to the second phone you share?
**Phone Yechitatu Yaanokumbidzana kana kushandisa nevamwe**	**Phone 3 Shared**
[Q212S]Foni yechitatu yaunokumbidzana nevamwe inonzii?	[Q212S]What is the make of the third phone you share?
[Q212S]Model yefoni yechitatu chii?	[Q212S]What is the model of the third phone you share?
[Q212S] Foni yechitatu iyi smart phone here?	[Q212S]Is the third phone you share a smart phone?
[Q212S] Foni yechitatu iyi wakaitenga rinhi?	[Q212S]Which year was the third phone you share purchased?
[Q213S] Unoshandisa kana kukumbidzana cell phone yechitatu nani?	[Q213S] Who do you share ownership of your third phone with?
[Q213S] Tsanangudza vamwe vanhu vaunoshandisa phone navo	[Q213C] Specify other
[Q213S] Kakawanda zvakadii kamunowana mukana wekushandisa phone yechitatu?	[Q232S] How often do you have access to the third phone you share?
**ZVEVANE PHONE KANA KUWANA MUKANA WE PHONE**	**FOR THOSE WITH PHONES OR ACCESS**
**MHANDO DZEMALINE DZINOSHANDISWA**	**MOBILE PROVIDERS USED**
[Q214prov] Unowanzoshandisa line ripi?	[Q214prov] Which mobile provider do you use normally?
[Q215]Unoshandisawo futi Econet	[Q215] Also use Econet network
[Q215] Unoshandisawo futi Telecel	[Q215] Also use Telecel network
[Q215] Unoshandisawo futi Netone	[Q215] Also use Netone network
[Q215]Unoshandisawo futi Africom	[Q215] Also use Africom network
**MARI DZINOSHANDISWA PA MOBILE PHONE**	**EXPENDITURE ON MOBILE PHONE**

[Q216air] Mari yekufonesa pa cell phone inogarotengwa here kana kuti inotengwa pamwedzi pamwedzi (contract)?	[Q216 air]For your phone, does airtime need to be purchased in advance or is it paid monthly through a contract?
[Q217amt] Pasvondo rapfuura, wakashandisa mari inosvika madhora mangani pa airtime?	[Q217amt] How much airtime, in dollars, did you use in the past week?
[Q218exp] Pasvondo rapfuura, wakashandisa mari inosvika madhora mangani pane zvimwe zvinhu zvenyu tisingasanganise ne airtime?	[Q218exp] How much money did you spend on other things for yourself in the past week excluding airtime for cell phones?
**NDIANI ANOWANZOBHADHARA MARI DZINOSHANDISWA PA MOBILE PHONE**	**WHO USUALLY PAYS FOR EXPENDITURE ON MOBILE PHONE**
[Q219]Ndini	[Q219]I do
[Q219]Vabereki/ Vanondichengeta	[Q219]My parents/ legal guardians
[Q219]Dzimwe Hama	[Q219]Other relative
[Q219]Shamwari	[Q219]Friend
[Q219]Mudiwa diwa	[Q219]Boyfriend/girlfriend
[Q219]Handishandise mari paairtime	[Q219]I don't spend money on airtime
[Q219]Vamwewo (Tsanangudza pazasi)	[Q219]Other (specify below)
[Q219_97]Handizivi	[Q219_97]Don't know
[Q219_99]Hapana	[Q219_99]No response
[Q219_other] Tsanangudza vamwe vano badhara	[Q219_other] specify Other person who pays for credit
[Q220_age] Wakatanga kushandisa cell phone une makore mangani?	[Q220_age] At what age did you start using a mobile phone?
**MIBVUNZO YEVANE PHONE CHETE**	**ONLY FOR THOSE WHO OWN A PHONE**
[Q221]Wakawana cell phone yako yekutanga sei?	[Q221] How did you get your first phone?
[Q221] Tsanangudza zvimwe zvakaita kuti uve nephone yekutanga	[Q221] Specify other reason why you got your first phone
[Q222] Akatenga cell phone yako yekutanga ndiyani?	[Q222]Who bought your first phone?
[Q222] Tsanangura mumwe akatenga phone	[Q222] Specify other person who bought first phone
**Inguva dzipi dzinowanzo kunge dzakadzima cell phone yako?**	**When is your phone usually switched off**
[Q223]Hapana pandinodzima	[Q223] Never
[Q223]Pandinenge ndakavata	[Q223]When I am sleeping
[Q223]Pandinenge ndiri kuchikoro/kubasa	[Q223]When I am at school/ work
[Q223]Kana ndisisina mari yekufonesa	[Q223]When I have run out of credit
[Q223]Kana bhatiri rava nemoto mushoma	[Q223]When the battery is low
[Q223]Dzimwewo nguva (tsanangurai pazasi)	[Q223]Other (specify below)
[Q223]Handizivi	[Q223]Don't know
[Q223]Hapana minduro	[Q223]No response
[Q223] Tsanangurai dzimwe nguva dzinodzimwa phone	[Q223]Specify other time phone is switched off
[Q224] Kakawanda zvakadii kamunoita kuti cell phone yenyu isarire zvinonzwika ('SILENT' or 'VIBRATE')?	[Q224] How often do you place your phone on 'SILENT' or 'VIBRATE' mode?
[Q225]Unotakura cell phone yako kuchikoro here?	[Q225] Do you take your cell phone to school ?
[Q226] Unogona kugara usina cell phone kwezuva rese here?	[Q226] Can you do without a mobile for a day?
[Q227] Kana ati kwete, nemhaka yei?	[Q227] Why cannot you not do without a phone
[Q228] Kwamuri chii chakanakira kuita cell phone?	[Q228] According to you, the best part of having a mobile phone is
[Q228] Tsanangurai zvimwe zvakanakira kuita cell phone?	[Q228] What are the other best part of having a phone
**Vasina Phone**	**For those with No Phone**
[Q229] Kana usina cell phone, nemhaka yei musina?	[Q229] If you don't have a mobile phone, kindly tell me why not?

[Q229] Zwimwe zvinoita kuti musave ne phone	[Q229] Whats the other reason for not having a phone
[Q235] Mune hurongwa here hwekutenga cell phone munguva inotevera?	[Q235] Are you planning to buy a mobile phone in near future?
[Q236] Kana mati hongu, riinhi?	[Q236] When are you planning to buy a mobile phone?
**Unoshandisa cell phone zvakadini kana uchiita izvi:**	**How frequently do you use a mobile phone for:**
[Q237]Kufona	[Q237]Phone calls
[Q237]Kutumira ma message kana pictures (kwete ne app)	[Q237]Sending text or picture messages (not via an app)
[Q237]Kutumira ma message kana chat (pa Whatsapp, Facebook)	[Q237]Sending instant messages or chat services (e.g Whatsapp, Facebook etc.)
[Q237]Kutamba ma games	[Q237]Playing games
[Q237]Kuona TV/movies/videos	[Q237]Watch TV/movies/videos
[Q237Kuterera kana ku downloader music/radio	[Q237]Listen to/ download music/radio
[Q237]Kunzwa news dzemitambo kana zvimwe	[Q237]Getting news updates (sports or other)
[Q237]Calculator	[Q237]Calculator
[Q237]]Social network yakafanana ne twitter, Instagram, Facebook	[Q237]]Social networking sites e.g. twitter, Instagram, Facebook
[Q237]Kubhadhara kana kutengesa zvinhu	[Q237]Making payments/ purchasing goods
**Unoshandisa cell phone zvakadini kana uchiita izvi:**	**How frequently do you use a mobile phone for:**
[Q237]Camera	[Q237]Camera
[Q237]Kufamba kwako nema maps	[Q237]Navigating with maps
[Q237]E-mailing	[Q237]E-mailing
[Q237]Uchitsvaga ruzivo rwezvekuchikoro uchiita homework kana zvekubasa	[Q237]Research for school/work or doing homework /revision e.g. Ruzivo
[Q237]Uchitsvaga ruzivo rwezveutano	[Q237]Search for information about my health
[Q237]Uchitsvaga ruzivo rwezvekudanana ne zvepabonde	[Q237]Search for information about relationships and sex
[Q237]Calendar	[Q237]Calendar
[Q237]Clock	[Q237]Clock
[Q237]Dictionary	[Q237]Dictionary
[Q237]Bible	[Q237]Bible
**Ndeapi ma apps matatu aunowanzoshandisa?**	**What are the three apps that you most often use ?**
[Q238_1]Sarudza app yokutanga	[Q238_1]Select the first app
[Q238_1]Tsanagudza rimwe zita	[Q238_1]Specify other
[Q238_2] Sarudza app yechipiri	[Q238_2]Select the second app
[Q238_2]Tsanagudza rimwe zita	[Q238_2]Specify other
[Q238_3]Sarudza app yechitatu	[Q238_3]Select the third app
[Q238_3]Tsanagudza rimwe zita	[Q238_3]Specify other
[Q239] Unombotamba ma games pa cell phone?	[Q239] Do you play any games on a cell phone?
**Ndeapi ma games matatu aunowanzotamba pa cell phone?**	**What are the three games you most often play on a cell phone?**
[Q239] Zita re Game rokutanga	[Q239] Name Game 1
[Q239] Tsanangudza Zita re Game rokutanga	[Q239] Specify Name Game 1
[Q239] Zita re Game rechipiri	[Q239] Name Game 2
[Q239] Tsanangudza Zita re Game rechipiri	[Q239] Specify Name Game 2
[Q239] Zita re Game rechitatu	[Q239] Name Game 3
[Q239] Tsanangudza Zita re Game rechitatu	[Q239] Specify Name Game 3
[Q240]Wakamboshandisa phone kuti uone kuti utano hwako hurikufamba sei?	[Q240] Have you ever used your phone for tracking your health?
[Q241]Nditaurire ma apps aunoshandisa kuona kuti utano hwako hwakamira sei	[Q241]Please list the applications that you use to track your personal health measures

**ZVAKAVANZIKA kana PRIVATE Dzako**	**PRIVACY**
[Q242]Unofunga kuti zvaunotumira paphone zviri 'private' kana zvakavanzika here?	[Q242]How 'private' do you consider the information that you send when using a phone?
[Q243]Unofunga kuti zvaunotumirwa paphone zviri 'private' kana zvakavanzika here?	[Q243]How 'private' do you consider the information that you receive when using a phone?
[Q244]Unofunga kuti zviri paphone zviri 'private' kana zvakavanzika here?	[Q244]How 'private' do you consider the information stored in your mobile phone?
[Q245] Une mapasswords yeku Locker phone yako here?	[Q245] Do you have passwords to lock/unlock your phone?
[Q246]Mune mapasswords eApplications ipi zvayo pacell phone yako?	[Q246]Do you have passwords for any applications on your phone?
[Q246b]Kana wati hongu, ndeapi maApplications anema passwords??	[Q246b]If yes, which ones?
**SECTION 3 PFUNGWA DZAKO - SSQ**	**SECTION 3 MENTAL HEALTH SSQ**
Iye zvinondichakubvunzai kuti mainzwa sei muvhiki rapfuura. Ndapota pindurai hongu kana kwete pamubvunzo umwe neumwe	Now I am going to ask you how you have been feeling over the past week. Please respond yes or no to each question
Muvhiki rapfuura	**In the past week**
[Q301]Pane pandaimboona ndichinyanya kufungisisa kana kufunga zvakawanda	[Q301]There were times in which I was thinking deeply or thinking about many things
[Q302]Pane pandaimbotadza kuisa pfungwa dzangu pamwechete	[Q302]I found myself sometimes failing to concentrate
[Q303]Pane pandaimboshatirwa kana kuita hasha zvenhando	[Q303]I lost my temper or got annoyed over trivial matters
[Q304]Pane pandaimborota hope dzinotyisa kana dzisina kunaka	[Q304]I had nightmares or bad dreams
[Q305]Pane pandaimboona kana kunzwa zvinhu zvaisaonekwa kana kunzwikwa nevamwe	[Q305]I sometimes saw or heard things which others could not see or hear
[Q306]Pane pandaimborwadziwa nemudumbu	[Q306]My stomach was aching
Muvhiki rapfuura	**In the past week**
[Q307]Pane pandaimbovhundutswa nezvinhu zvisina mature	[Q307]I was frightened by trivial things
[Q308]Pane pandaimbotadza kurara kana kushaya hope	[Q308]I sometimes failed to sleep or lost sleep
[Q309]Pane pandaimbonzwa kuomerwa neupenyu zvekuti ndaimbochema kana kunzwa kuda kuchema	[Q309]There were moments when I felt life was so tough that I cried or wanted to cry
[Q310]Pane pandaimbonzwa kuneta (kurukutika)	[Q310]I felt run down (tired)
[Q311]Pane pandaimboita pfungwa dzekuda kuzviuraya	[Q311]At times I felt like committing suicide
[Q312]Ndainzwa kusafara nezvinhu zvandaiita zuva nezuva	[Q312]I was generally unhappy with things that I would be doing each day
[Q313]Basa rangu rava kurasarira mumashure	[Q313]My work was lagging behind
[Q314]Ndainzwa zvichindiomera kuti ndoita zvipi	[Q314]I felt I had problems in deciding what to do
**PFUNGWA DZAKO - SDQ**	**MENTAL HEALTH AND WELL-BEING SDQ**
**Ndinokumbira kuti upe mhinduro dzako wakatarisa zvaiitika kana mamiriro epfungwa dzako mwedzi mitanhatu yapfuura.**	Please give your answers based on how things have been for you over the last six months.
**MUMWEDZI MITANHATU YAPFUURA NDAINZWA KANA KUFUNGA IZVI**	**MENTAL HEALTH AND WELL-BEING SDQ in the last six months**
[Q401]Ndinoyedza kuve munhu akanaka kune vamwe vanhu. Ndinoda kuita zvinoita kuti vafare	[Q401]I try to be nice to other people. I care about their feelings.
[Q402]Handigadzikane, handigoni kugara panzvimbo nguva yakareba	[Q402]I am restless, I cannot stay still for long
[Q403]Ndinogara ndichinzwa musoro, mudumbu nekungorwara rwara	[Q403]I get a lot of headaches, stomach-aches or sickness

[Q404]Nguva zhinji ndinogovana nevamwe(zvekudya, zvekutambisa, zvinyoreso nezvimwe)	[Q404]I usually share with others (food, games, pens etc.)
[Q405]Ndinotsamwa zvekutsamwa zviya zvakare ndinokurumidza kushatirwa	[Q405]I get very angry and often lose my temper
[Q406]Nguva zhinji ndinenge ndiri ndega. Ndinotamba ndega kana kugara ndega	[Q406]I am usually on my own. I generally play alone or keep to myself
[Q407]Ndinowanzoita zvandinenge ndaudzwa	[Q407]I usually do as I am told
[Q408]Ndinofunganya zvakanyanya	[Q408]I worry a lot
**MUMWEDZI MITANHATU YAPFUURA NDAINZWA KANA KUFUNGA IZVI**	**MENTAL HEALTH AND WELL-BEING SDQ in the last six months**
[Q409]Ndinobatsira kana mumwe munhu arwadziswa kana asiri kunzwa zvakanaka kana ashatiriswa	[Q409]I am helpful if someone is hurt, upset or feeling ill
[Q410]Ndinogara ndichingoshanyarika ndisingagadzikane	[Q410]I am constantly fidgeting or squirming
[Q411]Ndine shamwari imwechete kana dzakawanda	[Q411]I have one good friend or more
[Q412]Ndinorwa zvakanyanya .Ndinogona kuti vamwe vanhu vaite zvandinoda	[Q412]I fight a lot. I can make other people do what I want.
[Q413]Nguva zhinji ndinenge ndakasuwa, moyo wangu uchirwadza kana kuda kuchema	[Q413]I am often unhappy, down-hearted or tearful
[Q414]Vamwe vanhu vezera rangu vanondifarira	[Q414]Other people my age generally like me
[Q415]Ndinokurumidza kuita charinga, ndinotadza kunyatsoita sezvandinotarisirwa	[Q415]I am easily distracted, I find it difficult to concentrate
[Q416]Ndinonzwa kutya munzvimbo itsva. Ndinokurumidza kushaya chivimbo nezvandinenge ndichiita	[Q416]I am nervous in new situations. I easily lose confidence.
[Q417]Ndine tsitsi kuvanhu vechidiki	[Q417]I am kind to younger children.
[Q418]Ndinogara ndichipomerwa kunyepa nekusataura chokwadi	[Q418]I am often accused of lying or cheating
**MUMWEDZI MITANHATU YAPFUURA NDAINZWA KANA KUFUNGA IZVI**	**MENTAL HEALTH AND WELL-BEING SDQ in the last six months**
[Q419]Vamwe vana vanondisvora vachindichisidzira nekundirova kana kundituka kana kungoita zvinondidzikisira	[Q419]Other children or young people pick on me or bully me
[Q420]Kazhinji ndinobatsira vamwe(vabereki, vadzidzisi nevana)	[Q420]I often volunteer to help others (parents, teachers, children)
[Q421]Ndinonyatsofunga ndisati ndaita zvinhu	[Q421]I think before I do things
[Q422]Ndinotora zvinhu zvisiri zvangu kubva kumba, kuchikoro kana kumwewo	[Q422]I take things that are not mine from home, school or elsewhere
[Q423]Ndinowirirana zvakanaka nevanhu vakuru panevezera rangu	[Q423]I get on better with adults than with people my own age
[Q424]Ndine zvandinotya, ndinokurumidza kuvhunduka/kutya	[Q424]I have many fears, I am easily scared
[Q425]Ndinopedza basa randinenge ndichiita. Ndinonyatsoita sezvinotarisirwa	[Q425]I finish the work I'm doing. My attention is good

Table S1: Shona symptom questionnaire (SSQ): item response rates by questionnaire version used (n=634)

	**Total** [a]	**Version 1**	**Version 2**	**p-value for age-adjusted difference between versions** [b]
n	634	123	511	
Median SSQ (IQR)	7 (4, 9)	7 (4,9)	7 (4,9)	
Mean SSQ (95% CI)	6.44 (6.13, 6.74)	6.36 (5.60, 7.12)	6.46 (6.13, 6.79)	p=0.826
At risk for CMD (SSQ>=8)	37.38 (32.99, 41.99)	41.46 (32.40, 51.14)	36.40 (31.48, 41.62)	p=0.300
At risk for CMD (SSQ>=9)	28.55 (24.88, 32.53)	31.71 (24.33, 40.14)	27.79 (23.66, 32.33)	p=0.346
Severely at risk of CMD (SSQ>=11)	11.51 (9.08, 14.51)	13.82 (8.10, 22.59)	10.96 (8.42, 14.15)	p=0.410
I sometimes saw or heard things which others could not see or hear	9.78 (7.47, 12.71)	12.20 (7.07, 20.24)	9.20 (6.78, 12.36)	p=0.369
At times I felt like committing suicide	11.20 (9.01, 13.84)	14.63 (9.13, 22.64)	10.37 (8.10, 13.20)	p=0.166
Red flag	22.08 (18.55, 26.07)	27.64 (19.02, 38.32)	20.74 (17.04, 25.02)	p=0.160

a. mean and proportions taking into account clustered study design but not adjusted for age
b. Adjusted Wald test accounting for clustered study design and adjusting for age in years

Table S2 SSQ – item response rates by sex and overall response (yes, no) (n=634)

**Shona Symptom Questionnaire items**		**Males**		**Females**		**Total**
	N		N		N	
There were times in which I was thinking deeply or thinking about many things	153	64.56%	253	63.73%	406	64.04%
I found myself sometimes failing to concentrate	145	61.18%	225	56.68%	370	58.36%
I lost my temper or got annoyed over trivial matters	129	54.4%	237	59.7%	366	57.7%
I had nightmares or bad dreams	143	60.3%	257	64.7%	400	63.1%
I sometimes saw or heard things which others could not see or hear	34	14.4%	28	7.1%	62	**9.8%**
My stomach was aching	146	61.6%	273	68.8%	419	66.1%
I was frightened by trivial things	88	37.1%	150	37.8%	238	37.5%
I sometimes failed to sleep or lost sleep	119	50.2%	211	53.2%	330	52.1%

There were moments when I felt life was so tough that I cried or wanted to cry	116	49.0%	227	57.2%	343	54.1%
I felt run down (tired)	127	53.6%	242	61.0%	369	58.2%
At times I felt like committing suicide	17	7.2%	54	13.6%	71	**11.2%**
I was generally unhappy with things that I would be doing each day	91	38.4%	110	27.7%	201	31.7%
My work was lagging behind	85	35.9%	97	24.4%	182	28.7%
I felt I had problems in deciding what to do	117	49.4%	207	52.1%	324	51.1%

Table S3: Strength and Difficulties Questionnaire (SDQ): item response rates by sex and overall response (n=634)

**Mean SDQ scores**	All (n=634)			Males (n=237)			Females (n=397)		
	**Mean**	S.D.	Alpha	**Mean**	S.D.	Alpha	**Mean**	S.D.	Alpha
Difficulties total	**10.74**	6.25	0.75	**10.72**	6.23	0.74	**10.75**	6.28	0.75
Externalising score	**4.31**	3.48	0.68	**4.65**	3.59	0.67	**4.10**	3.41	0.69
Internalising score	**6.43**	3.94	0.63	**6.07**	3.69	0.59	**6.64**	4.08	0.64
Emotional symptoms	**3.66**	2.66	0.60	**3.38**	2.56	0.59	**3.83**	2.71	0.61
Conduct problems	**2.22**	1.97	0.46	**2.24**	2.09	0.49	**2.20**	1.89	0.46
Hyperactivity/inattention	**2.09**	2.03	0.52	**2.41**	2.07	0.50	**1.90**	1.99	0.54
Peer problems	**2.77**	2.07	0.38	**2.69**	1.94	0.33	**2.81**	2.15	0.41
Prosocial behaviour	**8.13**	2.22	0.68	**8.14**	2.24	0.69	**8.12**	2.20	0.67

Range of all scales 0-10, except Difficulties Total which ranges 0-40

Table S4 Mean SDQ scores by sex with adjustment for age and cluster sampling design

Mean SDQ scores	All (n=634)		Males (n=237)		Females (n=397)		Difference		
							Wald		
	Mean	95% CI	Mean	95% CI	Mean	95% CI	F	d.f.	P
Difficulties total	**10.74**	6.25	**10.71**	9.84 -11.58	**10.75**	10.12-11.38	0.01	1,123	0.9379
Externalising score	**4.31**	3.48	**4.63**	4.14-5.13	**4.11**	3.79-4.44	3.86	1,123	0.0517
Internalising score	**6.43**	3.94	**6.08**	5.58-6.57	**6.64**	6.23-7.05	3.02	1,123	0.0848
