# Supplementary material for: Common mental health and emotional and behavioural disorders among adolescents and young adults in Harare and Mashonaland East, Zimbabwe: a population-based prevalence study
Source: BMJ Open. 2023 Mar 14;13(3):e065276. doi: 10.1136/bmjopen-2022-065276 (PMC10016291; doi:10.1136/bmjopen-2022-065276)
Supplement: Author's manuscript [file bmjopen-2022-065276.draft_revisions.pdf]

BMJ Open is committed to open peer review. As part of this commitment we make the peer review history of every article we publish publicly available.

When an article is published we post the peer reviewers' comments and the authors' responses online. We also post the versions of the paper that were used during peer review. These are the versions that the peer review comments apply to.

The versions of the paper that follow are the versions that were submitted during the peer review process. They are not the versions of record or the final published versions. They should not be cited or distributed as the published version of this manuscript.

BMJ Open is an open access journal and the full, final, typeset and author-corrected version of record of the manuscript is available on our site with no access controls, subscription charges or pay-per-view fees (http://bmjopen.bmj.com).

If you have any questions on BMJ Open's open peer review process please email info.bmjopen@bmj.com

BMJ Open

Common mental health and behavioural disorders among adolescents and young adults in Harare and Mashonaland East, Zimbabwe: A population-based prevalence study

Journal:	*BMJ Open*
Manuscript ID	bmjopen-2022-065276
Article Type:	Original research
Date Submitted by the Author:	05-Jun-2022
Complete List of Authors:	Doyle, Aoife; London School of Hygiene & Tropical Medicine; Biomedical Research and Training Institute<br>Bandason, T; Biomedical Research and Training Institute<br>Dauya, E; Biomedical Research and Training Institute<br>McHugh, Grace; Biomedical Research and Training Institute<br>Grundy, Chris; London School of Hygiene & Tropical Medicine<br>Simms, Victoria; London School of Hygiene & Tropical Medicine; Biomedical Research and Training Institute<br>Chibanda, D; College of Health Sciences University of Zimbabwe, Department of Psychiatry; London School of Hygiene & Tropical Medicine<br>Ferrand, Rashida; Biomedical Research and Training Institute; London School of Hygiene & Tropical Medicine, Department of Clinical Research
Keywords:	MENTAL HEALTH, Child & adolescent psychiatry < PSYCHIATRY, EPIDEMIOLOGY

SCHOLARONE™
Manuscripts

I, the Submitting Author has the right to grant and does grant on behalf of all authors of the Work (as defined in the below author licence), an exclusive licence and/or a non-exclusive licence for contributions from authors who are: i) UK Crown employees; ii) where BMJ has agreed a CC-BY licence shall apply, and/or iii) in accordance with the terms applicable for US Federal Government officers or employees acting as part of their official duties; on a worldwide, perpetual, irrevocable, royalty-free basis to BMJ Publishing Group Ltd ("BMJ") its licensees and where the relevant Journal is co-owned by BMJ to the co-owners of the Journal, to publish the Work in this journal and any other BMJ products and to exploit all rights, as set out in our licence.

The Submitting Author accepts and understands that any supply made under these terms is made by BMJ to the Submitting Author unless you are acting as an employee on behalf of your employer or a postgraduate student of an affiliated institution which is paying any applicable article publishing charge ("APC") for Open Access articles. Where the Submitting Author wishes to make the Work available on an Open Access basis (and intends to pay the relevant APC), the terms of reuse of such Open Access shall be governed by a Creative Commons licence – details of these licences and which Creative Commons licence will apply to this Work are set out in our licence referred to above.

Other than as permitted in any relevant BMJ Author's Self Archiving Policies, I confirm this Work has not been accepted for publication elsewhere, is not being considered for publication elsewhere and does not duplicate material already published. I confirm all authors consent to publication of this Work and authorise the granting of this licence.

For peer review only

Common mental health and behavioural disorders among adolescents and young adults in Harare and Mashonaland East, Zimbabwe: A population-based prevalence study

Authors:

Doyle AM[1,2], Bandason T[2], Dauya, E[2], McHugh G[2], Grundy C[1], V Simms[1, 2], Chibanda D[3,4], Ferrand, RA[2,5]

Affiliations:

1 Medical Research Council International Statistics and Epidemiology Group, Faculty of Epidemiology and Population Health, London School of Hygiene & Tropical Medicine, London, UK

2 The Health Research Unit Zim (THRU ZIM), Biomedical Research and Training Institute, Zimbabwe

3 Department of Psychiatry, College of Health Sciences, University of Zimbabwe, Harare, Zimbabwe.

4 Centre for Global Mental Health, London School of Hygiene & Tropical Medicine, London, UK

5 Department of Clinical Research, London School of Hygiene & Tropical Medicine, London, UK

Corresponding Author:

Dr. Aoife Doyle

Medical Research Council International Statistics and Epidemiology Group

London School of Hygiene & Tropical Medicine

London WC1E7HT, UK

+447967497294

Aoife.doyle@lshtm.ac.uk

Ms Tsitsi Bandason

The Health Research Unit Zim (THRU ZIM)

Biomedical Research and Training Institute

Harare, Zimbabwe

vakoma@gmail.com

Ms Ethel Dauya

The Health Research Unit Zim (THRU ZIM)

Biomedical Research and Training Institute

For peer review only

For peer review only - http://bmjopen.bmj.com/site/about/guidelines.xhtml

Harare, Zimbabwe

edauya@brti.co.zw

Dr Grace Mc Hugh

The Health Research Unit Zim (THRU ZIM)

Biomedical Research and Training Institute

Harare, Zimbabwe

graceandandre@gmail.com

Dr Chris Grundy

Medical Research Council International Statistics and Epidemiology Group

London School of Hygiene & Tropical Medicine

London WC1E7HT, UK

Chris.grundy@lshtm.ac.uk

Dr Victoria Simms

Medical Research Council International Statistics and Epidemiology Group

London School of Hygiene & Tropical Medicine

London WC1E7HT, UK

Victoria.simms@lshtm.ac.uk

Dr Dixon Chibanda

Centre for Global Mental Health

London School of Hygiene & Tropical Medicine

London WC1E7HT, UK

Dixon.Chibanda@lshtm.ac.uk

Prof Rashida Ferrand

Department of Clinical Research

London School of Hygiene & Tropical Medicine

London WC1E7HT, UK

For peer review only

Rashida.ferrand@lshtm.ac.uk

Word count- ~4000 (4000 max for BMJ Open)

For peer review only

For peer review only - http://bmjopen.bmj.com/site/about/guidelines.xhtml

Abstract (300 words max): current 287

Objectives To estimate the prevalence of common mental health disorders (CMDs) and behavioural disorders among young people and to explore the correlates of CMDs risk.

Setting Five urban and peri-urban communities in Harare and Mashonaland East, Zimbabwe

Design Population-based cross-sectional study

Participants Young people aged 13-24 years living in households in the study areas

Outcome measures The primary outcome was the proportion of participants screening positive for probable CMDs defined as a Shona Symptoms Questionnaire (SSQ) score ≥8. Secondary outcomes were emotional and behavioural disorders measured using the Strength and Difficulties Questionnaire (SDQ), and adjusted odds ratios for factors associated with CMD.

Results Out of 634 young people, 37.4% (95% CI 33.0%, 42.0%) screened positive for probable CMDs, 9.8% (95% CI 7.5%, 12.7%) reported perceptual symptoms, and 11.2% (95%CI 9.0%, 13.8%) reported suicidal ideation. Using UK norms to define normal, borderline and abnormal scores for each of the SDQ domains, a high proportion (15.8%) of Zimbabwean young people had abnormal scores for emotional symptoms, and a low proportion had abnormal scores for hyperactivity/inattention scores (2.8%) and prosocial scores (7.1%). We created local cut-offs for the emotional symptoms, hyperactivity/attention, and prosocial SDQ domains. The odds of probable CMDs increased with each year of age (OR 1.09, p<0.001) and was higher among those who were out of school and not working compared to those in school or working (adj OR 1.67 (1.07, 2.62), p=0.04). One in five participants (22.1%) were referred immediately for further clinical assessment but uptake of referral services was low.

Conclusions We observed a high prevalence of symptoms of CMDs among general population urban and peri-urban young people especially among those with no employment. There is a need for more accessible and acceptable youth-friendly mental health services.

Strengths and limitations of this study

- In the context of limited population-level data on the mental health of young people, we included a representative general population sample of young people in five urban and peri-urban communities in Zimbabwe
- Demonstration of the feasibility of use of audio-computer assisted self-interview (ACASI) to collect self-reported measures of mental health and behavioural disorders in this young population.
- The SSQ and SDQ tools have not been extensively validated for use in the full age group and the translation of the SDQ into Shona did not follow recommended procedures. Additional validation studies are needed for tools to measure adolescent mental health.

For peer review only - http://bmjopen.bmj.com/site/about/guidelines.xhtml

Keywords:
*Adolescent; Young Adult; Cross-Sectional Studies; Mental health; Zimbabwe

Background:
Mental health disorders such as depressive, anxiety and childhood behavioural disorders are common among young people globally,[1] and in 2019 were among the top causes of morbidity among 10-19 year olds.[2] Poor mental health among adolescents is associated with increased risky behaviours,[3, 4] can impact on the management of other chronic health conditions, such as adherence to HIV treatment,[4-6] and can adversely affect educational and employment achievements.

Despite an increased focus on mental health programming and services, the global burden of mental health conditions is estimated to have remained relatively constant between 1990 and 2019.[1] In many low-and middle income settings, there are considerable treatment gaps including a lack of services and/or adequately trained staff.[7] Also, young people may not be aware of the available mental health services or find it difficult to attend health facilities as they are at school or at work. Additional barriers include stigma around mental health illness and young people's mistrust in service provision.

Understanding the epidemiology of mental health disorders in young people is essential to inform appropriate service provision and intervention development, and to inform policies and resource allocation. One of the challenges facing adolescent mental health programming for young people globally are gaps in data on the prevalence and determinants of disorders.[8, 9] In Zimbabwe, there are limited population-level data on the mental health of young people. A 2006 population-based survey on mental health disorders among rural 15- 23 year olds found 52% to be at risk of being affected and 24% to be at risk of being severely affected of common mental health disorders.[3] A 2009-2011 survey found a prevalence of psychological distress of 4.5% among males and 8.2% of females aged 15-24 years.[4] More recent published data on adolescent mental health in Zimbabwe is primarily from studies in adolescents living with HIV. For example, in 2016/17, 20.4% of adolescents living with HIV recruited into a trial at public clinics in two rural districts were at risk of CMDs.[10]

The primary aim of this study was to estimate the prevalence of common mental health disorders (CMDs) among young people. Secondary aims were to estimate the prevalence of behavioural disorders and to explore factors associated with CMDs.

Methods:
A cross-sectional population-based survey was conducted in 2018 among young people (13-24 years) in five communities in urban and peri-urban Harare and Mashonaland East, Zimbabwe selected for participation in a cluster randomised trial of a community-based, multi-component HIV and sexual and reproductive health intervention for young people (the CHIEDZA trial). The aim of the survey was to describe young people's access to and use of technology including mobile phones, and to describe their mental health and well-being. The mobile phone and technology use results have been reported separately.[11] The survey was undertaken as part of formative research to inform the trial intervention, and the findings informed the development of the technology and general health information and counselling components of the intervention. The survey communities were purposively selected to represent both urban and peri-urban communities. The trial protocol has been published,[12] and randomisation and trial implementation occurred after this survey was completed.

Eligible participants were aged 13 to 24 years, resident in the study community at the time of the survey, and either provided informed consent (16-24 years) or provided assent with guardian consent

For peer review only - http://bmjopen.bmj.com/site/about/guidelines.xhtml

(age 13-15 years). A simple random sample of 100 GPS coordinates (primary sampling unit) were sampled per cluster from all potential points in the study areas using ArcGIS software version 10.5 (Esri, Redlands, USA). Points were randomly ordered and then sequentially visited by a team of interviewers. All households with front doors within 20 meters of the sampled GPS point were visited. The household head was interviewed to obtain basic demographic information about the household and was asked for consent to interview any eligible YP. If the household head was not available, another household member aged 16+ years or a neighbour was asked to provide information on the composition of the household to determine potential household eligibility. Households with young people were visited a further two times in order to interview the household head. All young people in the selected households were eligible for recruitment. The target sample size of 686 would provide +/-6% precision around an expected prevalence of 50% or participants owning a mobile phone and assuming a design effect of 2 and 10% non-response.

Data collection

Participants responded to a short (approximately 30 minute) audio computer-assisted self-interviewing (ACASI) tablet-based questionnaire **(Supplementary material)**. Questions were adapted from pre-existing questionnaires.[13-16] The questionnaire was developed in English and translated into Shona (the local language) and participants could respond in English or Shona. Modifications were made to the questionnaire following pretesting with the study team and following the pilot survey which was conducted outside the selected study sites.

The Shona symptom questionnaire (SSQ) is a locally-validated 14 item scale asking about symptoms in the past week with dichotomised yes/no response categories.[17] Two different versions of the SSQ were used. For piloting and the first two weeks of data collection (~20% of data collection), we used a Shona version that had been created by the study team through translation of the English version of the SSQ into Shona so that it would be understandable by young people. For the remainder of the survey a self-completed version of the Shona SSQ which had previously been validated among 15-24 year olds was used.[18] Standard cut-offs of SSQ score >=8 indicate risk of being affected by CMD and a score >=11 indicate at risk of being severely affected by CMD [17].

The Strengths and Difficulties Questionnaire (SDQ) [19] is a widely-used freely- available 25 item scale which measures emotional and behavioural problems in the past six months. A scoping review of the use of SDQ in Africa found 54 studies in 12 countries, however, concluded that very little was known about the psychometric properties of SDQ in African settings. [20] A validated Shona version of the SDQ does not exist. Shona speaking study team members created a Shona version which was then back translated and checked by a psychiatrist (DC). The SDQ has five subscales: Emotional symptoms, conduct problems, hyperactivity, peer problems and prosocial behaviour.[21] Each scale has 5 items each with the response categories ('not true', 'somewhat true', and 'certainly true') associated with a score between 0 and 2. The mean SDQ-25 score is used as a measure of psychosocial well-being, with the maximum total SDQ score (sum of all scales except the prosocial scale) of 40. In low-risk or general population samples, responses to SDQ items can be used to create a three-subscale division i.e. internalising problems (sum of emotional and peer scales, 10 items), externalising problems (sum of conduct and hyperactivity scales, 10 items) and the prosocial scale (5 items).[22] Additionally, respondents can be described according to their SDQ 'caseness' profile: (i)Neither subscale elevated, (ii)elevated internalising sub-scale, (iii)elevated externalising sub-scale, and (iv) both sub-scales elevated. To facilitate the use of SDQ as a screening tool, UK norms for mean total SDQ and sub-scale scores have been created to allow identification of the 80th-90th centile (borderline) and >90th centile (abnormal) scores.[19] We used the self-report version of the SDQ questionnaire for adolescents 11-17 years (https://www.sdqinfo.org/py/sdqinfo/b0.py).

For peer review only - http://bmjopen.bmj.com/site/about/guidelines.xhtml

Data management and analysis

Data were collected and recorded using Open Data Kit (ODK) survey software with built-in logic checks and skip patterns on Android tablets. Data was analysed using STATA v17.0 (StatCorp, Tx, USA) using survey commands to account for the one-stage cluster sampling design.

The prevalence of common mental health disorders was described according to the standard adult cut-offs of SSQ score >=8 and score >=11 defining those at risk of being affected and severely affected by CMDs, respectively. We also described the prevalence of those at risk of being affected by CMD using a more conservative cut-off of SSQ>=9.[23] The Cronbach's alpha intra-class correlation coefficient was calculated to assess internal consistency of the SSQ scale with high internal consistency traditionally defined as alpha>0.7. Adjusted Wald tests were used to compare proportions between sub-populations. Age-adjusted Wald tests were used to explore potential differences in the SSQ findings according to the translation version of the questionnaire used.

Mean SDQ scores (total, domain and sub-scale) and their internal consistencies were described. UK norms, and where necessary Zimbabwe-specific norms, were used to define normal, borderline and abnormal scores for each of the domains. We calculated the mean, standard deviation, and Chronbach's alpha for the SDQ scores according to sex. Age-adjusted differences in means according to sex were examined taking into account the clustered sampling design. The proportion of this Zimbabwean sample in the 'normal', 'borderline' and 'abnormal' ranges were calculated based on cut-offs derived by the SDQ authors using UK data.[24] As the use of UK norms have been shown to be inappropriate in other settings,[25] Zimbabwean cut-offs were generated to classify the 80th-90th centile and >90th centile bands. Participants were described according to their SDQ 'caseness' profile.

Participants with missing data for one or more of the SSQ or SDQ questions were excluded from the analysis of that scale. Multiple logistic regression was used to explore the factors associated with risk of being affected by CMDs (SSQ>=8), with adjustment for the one-stage cluster sampling design. Potential explanatory variables were age, sex, marital status, community of residence, highest level of school attended, current occupational status, religion, travel for at least 1 month in past 12 months, length of time living in the community and orphanhood status, with age being considered an *a priori* potential confounder. Wald tests adjusted for the clustered sampling design were used at each step of the analysis. Community D, where only ten participants were interviewed due to logistical reasons, was excluded from regression analysis.

Ethical considerations

Ethical approval was obtained from Institutional Review Board of the Biomedical Research and Training Institute (BRTI, AP149/2018), the Medical Research Council of Zimbabwe (MCRZ, MRCZ/A/2362) and the London School of Hygiene and Tropical Medicine Research Ethics Committee (LSHTM REC, No.15919). Written informed consent was obtained from parents or guardians of study participants aged below 16 years, along with participant assent. Participants aged 16 years and older consented independently.

YP identified as 'red flags' based on their SSQ responses were referred immediately to health facilities for further assessment. Participants who scored 11 or higher on the SSQ or who responded 'yes' to either of the following questions were considered 'red flags': item 5 "In the past week, I sometimes saw or heard things which others could not see or hear"; item 11 "In the past week, at times I felt like committing suicide". In such instances, the research assistant was required to accompany the respondent to the nearest health facility so that they could seek professional care e.g. for mental health issues. At the time of study commencement, participants were referred to the "Friendship Bench" service at local government health facilities. The Friendship Bench lay health workers deliver basic cognitive behavioural therapy with an emphasis on problem solving therapy, activity scheduling, and peer led group support. However, local mental health services were reduced as staff were dealing

For peer review only - http://bmjopen.bmj.com/site/about/guidelines.xhtml

with a cholera outbreak and young people were reluctant to attend as they were not comfortable discussing their issues with the elderly counsellors. An alternative referral pathway was established, whereby, young people were referred to a psychiatrist's private clinic. Travel and initial consultation expenses were covered by the project and uptake of referrals was monitored through phone calls with the young people and through communication with the doctor.

Patient and public involvement

Patients and the public were not directly involved in the design, conduct or analysis of this study. Formative work conducted in 2018 in the study communities as part of the CHIEDZA trial involved workshops and interviews with young people with the aim of understanding their health priorities and health service preferences.

Results:

A total of 1212 households were sampled from 140 GPS point clusters in five communities (A 25, B 48, C 21, D 42, E 4) Figure 1. YP in the target age range (n=719) were identified in 491 households (41.1% of successfully interviewed households). Stratified sampling led to the recruitment of approximately 60% of participants from Harare, and 40% of the participants from Mashonaland East.

Figure 1: Survey recruitment (HH Household, YP young people)

INSERT Figure 1 HERE

Demographic profile of study population

A total of 634/719 (88.2%) eligible YP, mean age 18.0 years (SD 3.3) and 62.6% (397/634) female, participated. The majority (83.9%, 532/634) had never been married, 86.8% (550/634) had attended secondary school or higher, and only 14.7% (51/346) of out of school participants reported that they were working. Over a quarter (28.7%, 182/634) reported that they had travelled for at least one month in the past 12 months and 17.7% (112/634) had lived less than one year in their community. Approximately one third of respondents reported that one or both of their parents were dead or that a parent's location was unknown (**Table 1**).

Common mental health disorders

The median SSQ score was 7 (IQR 4, 9) overall, 6 (IQR 4,9) in males, and 7 (IQR 4,9) in females. Cronbach's alpha was 0.77 suggesting that the instruments are measuring the same construct (mood). In total, 37.38% (237/634) were at risk of CMD (SSQ >=8) and 11.51% (73/634) were severely at risk of CMD (SSQ >=11). Using a more conservative cut-off of SSQ>=9, 28.55% (181/634) were at risk of CMD. A fifth of those interviewed (22.08%) were identified as giving a red flag response i.e. had a SSQ >=11 or reported having seen or heard things which others could not see or hear (9.78% (62/634)) or that they felt like committing suicide in the past week 11.20% (71/634). The prevalence of CMD was similar according to the translation version of the questionnaire used **(Table S1**). The prevalence of CMD was similar among males and females. However, males were more likely than females to report having heard or seen things which others could not see or hear (14.35% vs. 7.05%, p=0.01)) and females were more likely to report suicidal ideation than males (13.60% vs. 7.17%, p=0.01). **(Table 2)**. Individual SSQ question responses and outcomes according to age group are presented in **Table S2**.

For peer review only - http://bmjopen.bmj.com/site/about/guidelines.xhtml

A total of 140 young people out of 634 (22.08%) were identified as needing immediate referral for assessment by a psychiatrist, however, we were only able to confirm that 23 of these young people (16%) were seen by the psychiatrist. The study team attempted to revisit the young people to encourage them to attend the referral but many refused, were too busy or had relocated. It is possible that some participants may have accessed the Friendship Bench at local health facilities.

For peer review only

For peer review only - http://bmjopen.bmj.com/site/about/guidelines.xhtml

Table 1: Demographic characteristics of the study population (N=634)

	Sex of respondent					
	Male (n=237, 37.4%)		Female (n=397, 62.6%)		Total (N=634)	
	No.	%	No.	%	No.	%
**Age group (years)**						
13-15y	65	27.4	96	24.2	161	25.4
16-17y	47	19.8	74	18.6	121	19.1
18-19y	55	23.2	89	22.4	144	22.7
20-24y	70	29.5	138	34.8	208	32.8
**Mean age (years)**	17.8 (17.4, 18.2)		18.2 (17.8, 18.5)		18.0 (17.8, 18.3)	
**Marital status**						
Married	5	2.1	64	16.1	69	10.9
Cohabiting	1	0.4	15	3.8	16	2.5
Never married	225	94.9	307	77.3	532	83.9
Divorced/separated	6	2.5	11	2.8	17	2.7
**Highest level of school attended**						
Primary	32	13.5	50	12.6	82	12.9
Secondary	195	82.3	330	83.1	525	82.8
Higher (Tertiary)	9	3.8	16	4	25	3.9
Never been to school	1	0.4	1	0.3	2	0.3
**Current occupational status**						
In school/university	121	51.1	167	42.1	288	45.4
Out of school (working)	22	9.3	29	7.3	51	8
Out of school (not working)	94	39.7	201	50.6	295	46.5
**Religion**[1]						
Roman Catholic	27	11.5	38	9.6	65	10.3
Protestant	55	23.5	102	25.7	157	24.9
Pentecostal	96	41.0	169	42.6	265	42.0
Apostolic	19	8.1	67	16.9	86	13.6
Other Christian/Muslim/Other	5	2.1	3	0.8	8	1.3
No religion	32	13.7	18	4.5	50	7.9
**Travelled for at least 1 month in past 12 months**						
No	170	71.7	282	71	452	71.3
Yes	67	28.3	115	29	182	28.7

For peer review only - http://bmjopen.bmj.com/site/about/guidelines.xhtml

**How long lived in community?** [2]						
<1 year	29	12.3	83	20.9	112	17.7
1-4 years	44	18.7	98	24.7	142	22.5
5+ years	162	68.9	216	54.4	378	59.8
**Orphan status**						
Double orphan	23	9.7	40	10.1	63	9.9
Mother dead, father alive	21	8.9	31	7.8	52	8.2
Mother alive, father dead or unknown	40	16.9	70	17.6	110	17.4
Both parents alive	153	64.6	256	64.5	409	64.5

[1]n=3 no response [2] n=2 don't know

For peer review only

Table 2: Shona symptom questionnaire (SSQ): item response rates by sex and overall response (n=634)

	**Total** [a]	**Male** [a]	**Female** [a]	**p-value for age-adjusted difference between sexes** [b]
n	634	237	397	
Median SSQ (IQR)	7 (4, 9)	6 (4,9)	7 (4,9)	
Mean SSQ (95% CI)	6.44 (6.13, 6.74)	6.37 (5.95, 6.79)	6.48 (6.11, 6.84)	p=0.811
At risk for CMD (SSQ>=8)	37.38 (32.99, 41.99)	34.18 (27.76, 41.23)	39.29 (34.11, 44.73)	p=0.278
At risk for CMD (SSQ>=9)	28.55 (24.88, 32.53)	26.58 (21.49, 32.39)	29.72 (25.22, 34.65)	p=0.443
Severely at risk of CMD (SSQ>=11)	11.51 (9.08, 14.51)	10.97 (7.86,15.11)	11.84 (8.79, 15.76)	p=0.803
I sometimes saw or heard things which others could not see or hear	9.78 (7.47, 12.71)	14.35 (10.03, 20.11)	7.05 (4.72, 10.41)	p=0.011
At times I felt like committing suicide	11.20 (9.01, 13.84)	7.17 (4.57, 11.09)	13.60 (10.69, 17.16)	p=0.012
Red flag	22.08 (18.55, 26.07)	21.10 (16.20, 27.00)	22.67 (18.36, 27.66)	p=0.693

a. mean and proportions taking into account clustered study design but not adjusted for age
b. Adjusted Wald test accounting for clustered study design and adjusting for age in years

Prevalence of behavioural disorders

For peer review only - http://bmjopen.bmj.com/site/about/guidelines.xhtml

The mean total difficulties SDQ score was 10.7 (SD 6.3, alpha 0.75), externalising symptoms score was 4.31 (SD 3.48, alpha 0.68) and internalising symptom score was 6.43 (SD 3.94, alpha 0.63). The mean scores for emotional symptoms, conduct problems, hyperactivity/inattention, and peer problems were 3.66, 2.22, 2.09 and 2.77 respectively. The mean prosocial behaviour score was 8.13 (**Table S3**).

Alpha was 0.75 for the total difficulties score and ranged from 0.38 to 0.68 for sub-scales. There was no evidence of a sex difference in total difficulties score and weak evidence of males having higher externalising scores than females (4.63 vs 4.11, p=0.05) and females having higher internalising scores than males (6.64 vs 6.08, p=0.08) (**Table S4**).

Using UK norms to define normal, borderline and abnormal scores for each of the domains, a high proportion (15.8%) of Zimbabwean young people had abnormal scores for emotional symptoms, and a low proportion had abnormal scores for hyperactivity/inattention scores (2.8%) and prosocial scores (7.1%). Using the Zimbabwean study data, we created country-specific cut-offs where the scores within each band differed for the emotional symptoms, hyperactivity/attention, and prosocial domains only. Using the Zimbabwean norms, a total of 13.9% had an elevated internalising sub-scale, 11.5% an elevated externalising sub-scale and 6.9% with both scales elevated (**Table 3**).

For peer review only

For peer review only - http://bmjopen.bmj.com/site/about/guidelines.xhtml

Table 3: Strength and Difficulties Questionnaire (SDQ): banding of self-report raw scores in Zimbabwe and UK

		UK norms				Zimbabwe norms			
			All (n=634)	Male (n=237)	Female (n=397)		All (n=634)	Male (n=237)	Female (n=397)
		Band	%	%	%	Band	%	%	%
Total Difficulties	Normal	0-15	78.4	78.9	78.1	0-15	78.4	78.9	78.1
	Borderline	16-19	11	11.4	10.8	16-19	11.0	11.4	10.8
	Abnormal	20-40	10.6	9.7	11.1	20-40	10.6	9.7	11.1
Emotional symptoms	Normal	0-5	74.1	77.6	72.0	0-5	74.1	77.6	72.0
	Borderline	6	10.1	10.1	10.1	6 to 7	13.7	13.9	13.6
	Abnormal	7 to 10	15.8	12.2	17.9	8 to 10	12.2	8.4	14.4
Conduct problems	Normal	0-3	74.9	75.1	74.8	0-3	74.9	75.1	74.8
	Borderline	4	12.8	11.0	13.9	4	12.8	11.0	13.9
	Abnormal	5 to 10	12.3	13.9	11.3	5 to 10	12.3	13.9	11.3
Hyperactivity/inattention	Normal	0-5	92.3	89.9	93.7	0-3	77.3	72.6	80.1
	Borderline	6	4.9	6.8	3.8	4	11.0	13.5	9.6
	Abnormal	7 to 10	2.8	3.4	2.5	5 to 10	11.7	13.9	10.3
Peer problems	Normal	0-3	64.7	66.2	63.7	0-3	64.7	66.2	63.7
	Borderline	4 to 5	22.7	23.2	22.4	4 to 5	22.7	23.2	22.4
	Abnormal	6 to 10	12.6	10.6	13.9	6 to 10	12.6	10.6	13.9
Prosocial behaviour	Normal	6 to 10	89.9	90.7	89.4	7 to 10	79.2	77.6	80.1

For peer review only

	Borderline	5	3.0	3.4	2.8	6	10.7	13.1	9.3
	Abnormal	0-4	7.1	5.9	7.8	0 to 5	10.1	9.3	10.6
SDQ caseness profile	Neither subscale elevated						67.7	67.9	67.5
	Elevated internalising sub-scale						13.9	10.1	16.1
	Elevated externalising sub-scale						11.5	15.6	9.1
	Elevated internalising and externalising sub-scale						6.9	6.3	7.3

For peer review only

Correlates of being at risk of common mental health disorders

The odds of CMD increased by 9% with each year increase in age (OR 1.09, p<0.001). Those who were out of school and not working had higher odds of CMD compared to those in school or those working (adjusted OR 1.67 (1.07, 2.62), p=0.04). Following adjustment for age, there was no evidence of an association between risk of CMD and participants' sex, marital status, education level, religion, travel in the past 12 months, length lived in the community or orphanhood status **(Table 4)**.

Table 4: Associations between demographic characteristics and >=8 score on SSQ

For peer review only

	N	n	%	Crude OR	Age Adj OR
**Sex**					
Male	237	81	34.18 (27.76, 41.23)	REF	REF
Female	387	150	38.76 (33.61, 44.18)	1.22 (0.87, 1.72)	1.19 (0.84, 1.67)
			p=0.254[a]	p=0.254	
**Age group**					
13-15y	159	42	26.42 (19.46, 34.79)	REF	
16-17y	119	47	39.50 (31.17, 48.48)	1.86 (1.14, 3.05)	
18-19y	144	58	40.28 (31.98, 49.18)	1.91 (1.16, 3.14)	
20-24y	202	84	41.58 (35.67, 47.75)	2.12 (1.37, 3.27)	
			p=0.0117		
**Age in years**				1.09 (1.04, 1.15)	
				p<0.001	
**Marital status**					
Married/Cohabiting	79	40	50.63 (40.54, 60.68)	REF	REF
Never married	528	182	34.47 (29.76, 39.51)	0.51 (0.32, 0.82)	0.68 (0.40, 1.13)
Widowed/Divorced/separated	17	9	52.94 (31.08, 73.73)	1.10 (0.37, 3.23)	1.06 (0.36, 3.17)
			p=0.0071	p=0.0035	p=0.22
**Highest level of school attended**					
None/primary	82	21	25.61 (16.10, 38.19)	REF	REF
Secondary	517	203	39.26 (34.37, 44.38)	1.88 (1.01, 3.51)	1.32 (0.67, 2.60)
Higher	25	7	28.00 (13.96, 48.25)	1.13 (0.43, 2.94)	0.57 (0.21, 1.56)
			p=0.0696	p=0.1228	p=0.1625
**Occupation**					
In school	287	83	28.92 (23.13, 35.50)	REF	REF
Out of school working	51	18	35.29 (22.70, 50.33)	1.34 (0.71, 2.52)	0.99 (0.49, 2.00)
Out of school not working	286	130	45.45 (39.45, 51.60)	2.05 (1.40, 2.99)	1.67 (1.07, 2.62)
			p=0.0006	p=0.0013	p=0.0410

For peer review only - http://bmjopen.bmj.com/site/about/guidelines.xhtml

**Religion**					
Roman Catholic	64	22	34.38 (23.59, 47.05)	REF	REF
Other Christian/ Muslim/No religion	557	208	37.34 (32.72, 42.21)	1.14 (0.65, 1.98)	1.19 (0.67, 2.11)
			p=0.6452	p=0.6454	p=0.5538
**Community of residence**					
A	178	71	39.89 (31.41,49.02)	REF	REF
B	140	48	34.29 (26.11,43.51)	0.78 (0.46, 1.35)	0.78 (0.45, 1.34)
C	147	64	43.54 (34.60,52.91)	1.16 (0.69, 1.97)	1.16 (0.68, 1.97)
E	159	48	30.19 (22.73,38.87)	0.65 (0.38, 1.11)	0.66 (0.38, 1.13)
				p=0.1625	p=0.1711
**Travelled for at last 1 month in last 12 months**					
No	444	161	36.26 (31.79, 40.99)	REF	REF
Yes	180	70	38.89 (30.47, 48.03)	1.12 (0.76, 1.65)	1.11 (0.75, 1.65)
			p=0.5705	p=0.5706	p=0.5980
**How long lived in the community**					
<1 year	112	42	37.50 (29.04, 46.80)	REF	REF
1-4 years	138	56	40.58 (32.45, 49.26)	1.14 (0.72, 1.81)	1.14 (0.70, 1.84)
5+ years	372	133	35.75 (30.37, 41.52)	0.93 (0.60, 1.44)	0.99 (0.64, 1.54)
			p=0.5865	p=0.6234	p=0.7846
**Orphan status**					
Double orphan	61	28	45.90 (33.22, 59.14)	REF	REF
Mother dead, father alive	52	25	48.08 (35.92, 60.46)	1.09 (0.51, 2.33)	1.08 (0.51, 2.31)
Mother alive, father dead	108	36	33.33 (24.94, 42.93)	0.59 (0.30, 1.15)	0.61 (0.31, 1.20)
Both parents alive	403	142	35.24 (29.83, 41.04)	0.64 (0.37, 1.11)	0.76 (0.44, 1.31)
			p=0.1118	p=0.0861	p=0.2217

[a] *p-value from adjusted wald test*

Note Community D (n=10) dropped from this analysis so total N is 624

Adjusted for age in years

Discussion:

Principal findings

We observed a high prevalence of self-reported symptoms of CMD in this population sample of 13-24 year olds in urban and peri-urban Zimbabwe. Over one in five young people required immediate referral for psychiatrist assessment but there was very low uptake of referral to mental health services. The odds of being affected by CMD increased with age and was higher among those out of

For peer review only

For peer review only - http://bmjopen.bmj.com/site/about/guidelines.xhtml

school and not working. Self-reported behavioural disorders were also common. Compared to UK norms for the Strength and Difficulties questionnaire, a lower proportion had abnormal scores for hyperactivity/inattention and prosocial sub-scales and a higher proportion had abnormal emotional symptoms scores which necessitated the creation of Zimbabwe-specific norms.

Common mental and behavioural disorders

We found 37% of young participants to be at risk of CMD (SSQ>=8), and 12% to be severely at risk of CMD (SSQ>=11). High prevalence of symptoms of CMD was also observed in a 2006 population-based survey among rural Zimbabwean youth (15-23 years)[3], and among HIV+ adolescents in Harare in 2009 [5] and in two rural districts in 2016/17.[10] Considerably lower prevalence of symptoms of CMDs was observed in a 2009-11 general population survey of 15-24 year olds.[4] [26] While all these studies used the SSQ, it is difficult to make comparisons given the different populations, socio-economic environments, and modes of data collection used i.e. self-completed vs interviewer administered. Previous research has shown that symptom reporting varied according to method with higher reporting in studies using self-administered methods.[27] The observed increase in risk of CMD with age is consistent with the literature.[28-31] We did not observe a higher prevalence of CMD among females compared to males which is in contrast to other adolescent studies. [32, 33]

Similar high prevalence of CMD has been observed in systematic reviews of studies among adolescents in sub-Saharan Africa with estimated prevalence of 27% for depression and 30% for anxiety disorders,[28] and 30-50% for emotional or behavioural difficulties or significant psychological distress in adolescents with HIV.[29]

The high prevalence of symptoms of CMD among young people in Zimbabwe may in part be due to the challenging economic situation with limited job opportunities, and the HIV and associated orphanhood epidemic.[34] Higher prevalence of CMD symptoms among those out of school and not working in this study has also been observed in HIV+ adolescent populations.[29] Interviews with adolescents who participated in the recent youth version of the Friendship Bench suggest that hopelessness and a feeling of lack of control over one's situation have an important impact on mental wellbeing.[35]

Measurement of common mental health and behavioural disorders

This study demonstrated that self-reported SSQ and SDQ are feasible to measure in 13-24 year olds in urban and peri-urban Zimbabwe. While the SSQ has been validated in several populations,[17, 23] there is no consensus on the most appropriate cut-offs to be used for young people. [3, 10, 26] Some authors have found that a cut-off of ≥5 provides the best performance and increases sensitivity of the screening tool, [26] however, their conclusions were based on interviewer-administered questionnaires where some of the SSQ questions had non-standard wording. A more recent validation of SSQ against MINI-KID in 12-17 year olds attending primary care clinics in urban Zimbabwe found the optimal cutpoint of SSQ >=8 (Unpublished data).

In this first documented use of a Shona language version of the Strength and difficulties questionnaire we observed good internal consistency for total difficulties but lower consistency within some of the sub-scales. In line with studies in other sub-Saharan African settings, the translation of a few of the terms within the SDQ were challenging,[20] for example, in the hyperactivity sub-scale there is a question about 'fidgeting' for which there is no equivalent term in Shona. The patterns of behavioural disorders observed in this study (lower abnormal prosocial and hyperactivity sub-scale scores, higher emotional and peer sub-scale scores) are similar to patterns observed in studies of South African adolescents [25] and children[36], and Namibian adolescents.[37]

For peer review only - http://bmjopen.bmj.com/site/about/guidelines.xhtml

Multiple screening tools exist for common mental health disorders and while the SSQ is widely used in Zimbabwe, and the SDQ used widely globally, there may be other tools that are more appropriate for the Zimbabwean adolescent population. A first step will therefore be to decide on which tool or tools are most promising and then plan the necessary careful adaptation and validation work. UNICEF with WHO and other partners are currently developing tools for the Measurement of Mental Health Among Adolescents at the Population Level (MMAP) with a focus on PHQ-9 and GAD-7.[38] If the SDQ is to be used again in Zimbabwean adolescents then additional work is needed to develop a Shona version and then to develop country-specific SDQ cut-offs for 'caseness'. [25]

Services for young people

The high burden of CMD among young people and reluctance to attend adult mental health services demonstrate an urgent need for youth-friendly mental health services. The original referral pathway was to refer the young people to the Friendship Bench Service at nearby local government health facilities. However, health facility staff shortages due to an ongoing cholera outbreak coupled with YPs reluctance to access adult Friendship Bench services required the creation of an alternative referral pathway. While some young people successfully attended referral appointments at the hospital in Harare, there were many young people who did not attend care. This study has highlighted the need to involve young people when developing research study protocols and to establish the feasibility and acceptability of proposed referral pathways. In addition to increasing the availability of high-quality youth-friendly mental health services, increased education and skill-building for young people and their families may help to support healthy mental health and well-being.[31]

Strengths and weaknesses of the study

A strength of this study is a representative general population sample of young people in five urban and peri-urban communities in Zimbabwe, in the context of limited population-level estimates of mental health disorders among young people. We demonstrated the feasibility of using ACASI to collect self-reported measures of mental health and behavioural disorders in this young population. Only the self-report version of the SDQ was used, however, for optimal use it is recommended that teachers and parents also complete the SDQ allowing triangulation of data. [39] The tools used were not validated for use in the full age group, the translation of the Strength and Difficulties Questionnaire into Shona did not follow recommended procedures, and two different Shona versions of SSQ were used. The self-reported measures used may have been subject to recall bias and social desirability bias.

Conclusion:

We observed a high prevalence of probable CMDs among general population urban and peri-urban young people, however there was a low uptake of referrals to existing mental health services. The use of self-reported SSQ and SDQ were feasible in this population but additional research is needed to determine the validity and most appropriate cut-offs for these measurement tools in young Zimbabweans. Important outstanding research questions for young people's mental health in this resource-constrained setting include how to identify those most at risk, and how to ensure that they are linked to appropriate youth-focused mental health services.

Author contributions

Obtained funding: AMD, RAF

For peer review only - http://bmjopen.bmj.com/site/about/guidelines.xhtml

For peer review only

Wrote the study protocol: AMD, TB, ED, GM, CG, DC, RAF

Assisted with fieldwork and data collection which was overseen by: AMD, ED, TB

Did central data monitoring: TB

Conducted statistical analysis: AMD

Advised on statistical analysis: VS

Drafted the initial manuscript: AMD

Provided substantial edits to the manuscript: TB, ED, GM, CG, VS, DC, RAF

All authors contributed to interpretation of the results and have read and approved the final manuscript

Funding

Financial support for this study was provided through joint funding under the UK Medical Research Council (MRC)/ UK Department for International Development (DFID) Concordat agreement which is supported by the European Union under the EDCTP2 programme (reference MR/K012126/1) with AMD receiving support (G0700837). AMD is supported by a UKRI Future Leader Fellowship [grant number MR/T043156/1]. RAF is funded by the Wellcome Trust through a Senior Fellowship in Clinical Science (206316Z/17/Z).

Disclaimer

The funders had no role in study design, data collection and analysis, decision to publish or preparation of the manuscript.

Competing interests

None declared

Data availability

Data are available upon reasonable request. Data for this study are available upon reasonable request to the principal authors.

Acknowledgements

We thank the study participants and their communities for participating in and facilitating this research. We are grateful to the field team who conducted the interviews, and the other Biomedical Research and Training institute, Harare, who supported the study.

Abbreviations

CMDs Common mental health disorders

SDQ Strength and Difficulties Questionnaire

ACASI Audio-computer assisted self-interview

SSQ Shona Symptoms Questionnaire

ODK Open Data Kit

For peer review only - http://bmjopen.bmj.com/site/about/guidelines.xhtml

Ethics Approval Statement

Ethical approval was obtained from Institutional Review Board of the Biomedical Research and Training Institute (BRTI, AP149/2018), the Medical Research Council of Zimbabwe (MCRZ, MRCZ/A/2362) and the London School of Hygiene and Tropical Medicine Research Ethics Committee (LSHTM REC, No.15919). Written informed consent was obtained from parents or guardians of study participants aged below 16 years, along with participant assent. Participants aged 16 years and older consented independently.

References:

1. Global Burden of Disease, *Global, regional, and national burden of 12 mental disorders in 204 countries and territories, 1990-2019: a systematic analysis for the Global Burden of Disease Study 2019.* Lancet Psychiatry, 2022.
2. Guthold, R., et al., *The Top Global Causes of Adolescent Mortality and Morbidity by Age and Sex, 2019.* J Adolesc Health, 2021. **69**(4): p. 540.
3. Langhaug, L.F., et al., *High prevalence of affective disorders among adolescents living in Rural Zimbabwe.* J Community Health, 2010. **35**(4): p. 355-64.
4. Tlhajoane, M., et al., *Prevalence and Associations of Psychological Distress, HIV Infection and HIV Care Service Utilization in East Zimbabwe.* AIDS Behav, 2018. **22**(5): p. 1485-1495.
5. Mavhu, W., et al., *Enhancing psychosocial support for HIV positive adolescents in Harare, Zimbabwe.* PLoS One, 2013. **8**(7): p. e70254.
6. Willis, N., et al., *Understanding the experience and manifestation of depression in adolescents living with HIV in Harare, Zimbabwe.* PLoS One, 2018. **13**(1): p. e0190423.
7. Mangezi, W. and D. Chibanda, *Mental health in Zimbabwe.* Int Psychiatry, 2010. **7**(4): p. 93-94.
8. Guthold, R., et al., *Priority Areas for Adolescent Health Measurement.* J Adolesc Health, 2021. **68**(5): p. 888-898.
9. Erskine, H.E., et al., *The global coverage of prevalence data for mental disorders in children and adolescents.* Epidemiol Psychiatr Sci, 2017. **26**(4): p. 395-402.
10. Mavhu, W., et al., *Effect of a differentiated service delivery model on virological failure in adolescents with HIV in Zimbabwe (Zvandiri): a cluster-randomised controlled trial.* Lancet Glob Health, 2020. **8**(2): p. e264-e275.
11. Doyle, A.M., et al., *Mobile Phone Access and Implications for Digital Health Interventions Among Adolescents and Young Adults in Zimbabwe: Cross-Sectional Survey.* JMIR Mhealth Uhealth, 2021. **9**(1): p. e21244.
12. Dziva Chikwari, C., et al., *The impact of community-based integrated HIV and sexual and reproductive health services for youth on population-level HIV viral load and sexually transmitted infections in Zimbabwe: protocol for the CHIEDZA cluster-randomised trial [version 1; peer review: awaiting peer review].* Wellcome Open Research, 2022. **7**(54).
13. GSMA, *A toolkit for researching women's internet access and use*. 2018, GSM Association. p. 48.
14. MACRO, *Study of Mobile Phone Usage Among the Teenagers and Youth In Mumbai*. 2004, MACRO.
15. Kreutzer, T., *Online and Digital Media Usage on Cell Phones among low-Income Urban Youth in Cape Town* in *Centre for Film and Media Studies*. 2009, University of Cape Town: Cape Town. p. 123.
16. UNICEF, *mHealth and Young People in South Africa*. 2017. p. 48.

For peer review only - http://bmjopen.bmj.com/site/about/guidelines.xhtml

17. Patel, V., et al., *The Shona Symptom Questionnaire: the development of an indigenous measure of common mental disorders in Harare.* Acta Psychiatr Scand, 1997. **95**(6): p. 469-75.
18. Verhey, R., J.D. Turner, and D. Chibanda, *The Friendship Bench Training Manual*.
19. Goodman, R., *The Strengths and Difficulties Questionnaire: a research note.* J Child Psychol Psychiatry, 1997. **38**(5): p. 581-6.
20. Hoosen, N., et al., *The Strengths and Difficulties Questionnaire (SDQ) in Africa: a scoping review of its application and validation.* Child Adolesc Psychiatry Ment Health, 2018. **12**: p. 6.
21. Goodman, R., *Psychometric properties of the strengths and difficulties questionnaire.* J Am Acad Child Adolesc Psychiatry, 2001. **40**(11): p. 1337-45.
22. Goodman, A., D.L. Lamping, and G.B. Ploubidis, *When to use broader internalising and externalising subscales instead of the hypothesised five subscales on the Strengths and Difficulties Questionnaire (SDQ): data from British parents, teachers and children.* J Abnorm Child Psychol, 2010. **38**(8): p. 1179-91.
23. Chibanda, D., et al., *Validation of screening tools for depression and anxiety disorders in a primary care population with high HIV prevalence in Zimbabwe.* J Affect Disord, 2016. **198**: p. 50-5.
24. *Scoring the Strengths & Difficulties Questionnaire for age 4-17*. 2014 [cited 2022 25 Jan 2022]; Available from: https://www.ehcap.co.uk/content/sites/ehcap/uploads/NewsDocuments/236/SDQEnglishUK4-17scoring-1.PDF.
25. de Vries, P.J., et al., *Measuring adolescent mental health around the globe: psychometric properties of the self-report Strengths and Difficulties Questionnaire in South Africa, and comparison with UK, Australian and Chinese data.* Epidemiol Psychiatr Sci, 2018. **27**(4): p. 369-380.
26. Haney, E., et al., *One size does not fit all: psychometric properties of the Shona Symptom Questionnaire (SSQ) among adolescents and young adults in Zimbabwe.* J Affect Disord, 2014. **167**: p. 358-67.
27. Langhaug, L.F., et al., *Difference in prevalence of common mental disorder as measured using four questionnaire delivery methods among young people in rural Zimbabwe.* J Affect Disord, 2009. **118**(1-3): p. 220-3.
28. Jörns-Presentati, A., et al., *The prevalence of mental health problems in sub-Saharan adolescents: A systematic review.* PLoS One, 2021. **16**(5): p. e0251689.
29. Dessauvagie, A.S., et al., *The prevalence of mental health problems in sub-Saharan adolescents living with HIV: a systematic review.* Glob Ment Health (Camb), 2020. **7**: p. e29.
30. Too, E.K., et al., *Prevalence and factors associated with common mental disorders in young people living with HIV in sub-Saharan Africa: a systematic review.* J Int AIDS Soc, 2021. **24 Suppl 2**(Suppl 2): p. e25705.
31. WHO, *Global accelerated action for the health of adolescents (AA-HA!): guidance to support country implementation*. 2017. p. 148.
32. Anjum, A., et al., *Investigating the prevalence of and factors associated with depressive symptoms among urban and semi-urban school adolescents in Bangladesh: a pilot study.* Int Health, 2019.
33. Girma, S., et al., *Depression and its determinants among adolescents in Jimma town, Southwest Ethiopia.* PLoS One, 2021. **16**(5): p. e0250927.
34. Nyamukapa, C.A., et al., *Causes and consequences of psychological distress among orphans in eastern Zimbabwe.* AIDS Care, 2010. **22**(8): p. 988-996.
35. Broström, S., et al., *"Seeing a Brighter Future" - Experiences of Adolescents with Common Mental Disorders Receiving the Problem-Solving Therapy "Youth Friendship Bench" in Zimbabwe.* Issues Ment Health Nurs, 2021. **42**(11): p. 1019-1029.

For peer review only - http://bmjopen.bmj.com/site/about/guidelines.xhtml

36. Mellins, C.A., et al., *Screening for Mental Health Among Young South African Children: The Use of the Strengths and Difficulties Questionnaire (SDQ).* Glob Soc Welf, 2018. **5**(1): p. 29-38.
37. Gentz, S.G., et al., *Predictors of mental health problems in adolescents living with HIV in Namibia.* Child Adolesc Ment Health, 2017. **22**(4): p. 179-185.
38. Carvajal, L., J.H. Requejo, and C.E. Irwin, *The Measurement of Mental Health Problems Among Adolescents and Young Adults Throughout the World.* J Adolesc Health, 2021. **69**(3): p. 361-362.
39. Goodman, R., et al., *Using the Strengths and Difficulties Questionnaire (SDQ) to screen for child psychiatric disorders in a community sample.* Br J Psychiatry, 2000. **177**: p. 534-9.

For peer review only

For peer review only - http://bmjopen.bmj.com/site/about/guidelines.xhtml

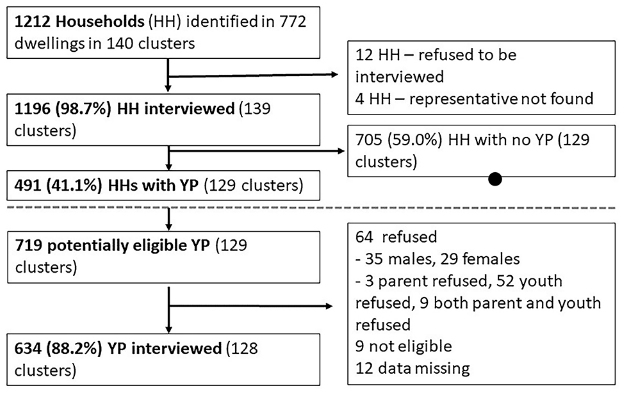
1212 Households (HH) identified in 772 dwellings in 140 clusters
12 HH – refused to be interviewed
4 HH – representative not found
1196 (98.7%) HH interviewed (139 clusters)
705 (59.0%) HH with no YP (129 clusters)
491 (41.1%) HHs with YP (129 clusters)
719 potentially eligible YP (129 clusters)
64 refused
- 35 males, 29 females
- 3 parent refused, 52 youth refused, 9 both parent and youth refused
9 not eligible
12 data missing
634 (88.2%) YP interviewed (128 clusters)

Supplementary Material

Questionnaire (YTM Q02 n6)

Shona	English
**SECTION 0 RECRUITMENT INFORMATION**	**SECTION 0 RECRUITMENT INFORMATION**
[Q01] Date of Interview	[Q01] Date of Interview
[Q02] Whats the Suburb Name?	[Q02] Whats the Suburb Name?
[Q022] Whats the Suburb Name?	[Q022] Whats the Suburb Name?
[Q03] Cluster ID?	[Q03] Cluster ID?
[Q032] Cluster ID?	[Q032] Cluster ID?
[Q04] Dwell ID?	[Q04] Dwell ID?
[Q042] Dwell ID?	[Q042] Dwell ID?
[Q05] Household ID?	[Q05] Household ID?
[Q05m] Manually enter the Household ID?	[Q05m] Manually enter the Household ID?
[Q05m] Manually enter the Household ID?	[Q05m] Manually enter the Household ID?
[Q06] Respondent No?	[Q06] Respondent No?
[Q062] Respondent No?	[Q062] Respondent No?
[Q07] Select your name	[Q07] Select your name
[Q08] Please select the language for this interview	[Q08] Please select the language for this interview
[Q101] Mukomana kana musikana here?	[Q101] Respondent Sex
[Q102] Wave nemakore mangani?	[Q102] How old were you at your last birthday?
[Q103] Panguva ino wakamira sei panyaya dzewanano?	[Q103] What is your current marital status?
[Q104] Wakambopinda chikoro here?	[Q104] Have you ever attended school?
[Q106] Kuchikoro wakasvika pai: primary, secondary, kana pamusoro?	[Q106] What is the highest level of school you attended: primary, secondary, or higher?
[Q105] Ndezvipi zvinonyatsotsanangura zvauri kuita mazuvano?	[Q105] Which of the following best describes your current occupational status?
[Q107] Uri vechitendero chipi?	[Q107] What is your religion?
[Q107] Tsanangudza chimwe chitendero?	[Q107] Specify other religion?
[Q108] Unowanzotaura mutauro upi kana usiri kumba?	[Q108] Outside your home, what language do you speak the most?
[Q108] Tsanangudza mumwe mutauro waunotaura usiri pamba?	[Q108] Specify other language?
**NGUVA YAWAGARA PAMBA PANO KANA MUNHARAUNDA INO**	**DURATION OF STAY AT RESIDENCE**
[Q109] Wave nenguva yakareba zvakadii uchigara munharaunda ino?	[Q109] How long have you lived in this community?
[Q110] Mumwedzi gumi nemiviri yapfuura, wakambofamba wakanogara kuneimwe nzvimbo kwemwedzi kana kupfuura here?	[Q110] In the last 12 months, have you travelled and stayed in a place other than this community for one month or more?
[Q111] Kusanganisa nguva yese yawakagara kunze kwenharaunda ino, ungati wakagara nguva yakadii mumwedzi negumi nemiviri yapfuura?	[Q111] In total approximately how long have you spent outside this community in the last 12 months?
**MHURI**	**FAMILY**

For peer review only

[Q112] Baba vako vakakubereka vapenyu here?	[Q112] Is your natural father alive?
[Q113] Mai vako vakakubereka vapenyu here?	[Q113] Is your natural mother alive?
Chimbonditaurira nezvinhu zve MICHINA (TECHNOLOGY) zvamunazvo mumba menyu	Please tell us about some technological devices- which of the following do you have at home
**MICHINA (TECHNOLOGY) IRI MUMBA**	**TECHNOLOGY AT HOME**
[Q201h] Normal/'desktop' computer	[Q201h] Normal/'desktop' computer
[Q201h] Laptop computer (including notebooks and netbooks)	[Q201h] Laptop computer (including notebooks and netbooks)
[Q201h] Tablet/ iPad	[Q201h] Tablet/ iPad
[Q201h] Feature (non-smart) Cell phone	[Q201h] Feature (non-smart) Cell phone
[Q201h] Basic feature (calls and sms only) Cellphone	[Q201h] Basic feature (calls and sms only) Cellphone
[Q201h] Smart cell phone	[Q201h] Smart cell phone
[Q201h6] Ipod or other MP3 player	[Q201h6] Ipod or other MP3 player
[Q201h] TV	[Q201h] TV
[Q201h] Radio	[Q201h] Radio
[Q201h] Digital camera	[Q201h] Digital camera
[Q201h] Gaming console (Playstation, Xbox, Nintendo wii etc.)	[Q201h] Gaming console (Playstation, Xbox, Nintendo wii etc.)
[Q201h] Handheld gaming device (e.g. DS, PSP, …)	[Q201h] Handheld gaming device (e.g. DS, PSP, …)
**UNOSHANDISA MICHINA(TECHNOLOGY) IPI**	**USE OF TECHNOLOGY**
[Q201u] Normal/'desktop' computer	[Q201u] Normal/'desktop' computer
[Q201u] Laptop computer (including notebooks and netbooks)	[Q201u] Laptop computer (including notebooks and netbooks)
[Q201u] Tablet/ iPad	[Q201u] Tablet/ iPad
[Q201u] Feature (non-smart) Cell phone	[Q201u] Feature (non-smart) Cell phone
[Q201u] Basic feature (calls and sms only) Cellphone	[Q201u] Basic feature (calls and sms only) Cellphone
[Q201u] Smart cell phone	[Q201u] Smart cell phone
[Q201u6] Ipod or other MP3 player	[Q201u6] Ipod or other MP3 player
[Q201u] TV	[Q201u] TV
[Q201u] Radio	[Q201u] Radio
[Q201u] Digital camera	[Q201u] Digital camera
[Q201u] Gaming console (Playstation, Xbox, Nintendo wii etc.)	[Q201u] Gaming console (Playstation, Xbox, Nintendo wii etc.)
[Q201u] Handheld gaming device (e.g. DS, PSP, …)	[Q201u] Handheld gaming device (e.g. DS, PSP, …)
[Q202] Wakamboshandisa internet here?	[Q202] Have you ever used the internet??
**KUSHANDISA INTERNET**	**USE OF INTERNET**
[Q203] Kana wakashandisa, wakapedzisira kushandisa internet rinhi?	[Q203] If yes, when is the last time that you used the internet?
Bvunza kuti anoshandisa internet kupi, nguva yakadini	Ask about when the participants uses the internet, how often do they do so
**Unoshandisa Internet nguva yakadini**	**How ofte do you use internet**
[Q204]Pacomputer kuchikoro kana kubasa	[Q204]On a computer at school/ work
[Q204]Pacomputer kumba	[Q204]On a computer at home
[Q204]Pacell/mobile phone	[Q204]On a cell/mobile phone
[Q204]Pacomputer kulibrary/ kana community internet shop	[Q204]At a computer in the library/ other community internet facility
[Q204]Ku internet café inobhadharwa	[Q204]At a commercial internet café- where you pay for the access
[Q204]Pacomputer kumba kwevamwewo	[Q204]At a computer in someone else's house
[Q205] Unopedza nguva yakadini uri painternet?	[Q205] On average how much time do you spend on the internet when you go online?
[Q206] Chii chaunofarira kunyanya painternet?	[Q206] What do you like the most about the internet?

For peer review only

[Q206] Tsanangudza chimwe chikonzero chaunofarira painternet?	[Q206] Specify other reason what you like about the internet?
[Q207] Chii chausinga farire kunyaya painternet?	[Q207] What do you dislike the most about the internet?
[Q207] Tsanangudza chimwe chikonzero chausingafarire painternet?	[Q207] Specify other reason what you dislike about the internet?
[Q208] Chii chaungade kuti chishanduke kuti kushandisa kwako internet kuve nani?	[Q208] What one change would you like to see so that use of the internet could be better for you?
[Q208] Tsanangudza chimwe chinhu chaungade kuti chishanduke?	[Q208] Specify other change you would like to see?
[Q209] Une cell phone here kana kuti unoshandisa cell phone yaunokumbidzana kana kushandisa nevamwe?	[Q209] Do you have or share mobile (cell) phone)?
**VANE MOBILE PHONE KANA VANOSHANDISA**	**OWNERSHIP AND USE OF MOBILE PHONE**
[Q209a] Kana ati hongu; Une cell phone ngani dzako pachako?	[Q209a] If Yes, how many phones you own by yourself ?
[Q209b] Kana ati hongu; Une cell phone ngani dzaunokumbidzana kushandisa nevamwe?	[Q209b] If Yes, how many phones do share with someone else?
[Q210] Une maline mangani e cell phone aunokushandisa parizvino?	[Q210] How many mobile phone numbers do you currently use?
[Q211] Mugore rapfuura, maline mangani e cell phone yawakanga unayo kusanganisira aunawo parizvino?	[Q211] How many different mobile phone numbers have you had in the past year including your current number (s)?
Ngatimbotaura nema cell phone ${Q209amob} awunawo	Lets talk about the ${Q209amob} mobile phones you own
**MHANDO DZEMAMOBILE PHONE DZAANADZO**	**MOBILE PHONE TYPES OWNED**
**Phone Yokutanga Yaanayo**	**Phone 1 Owned**
[Q212O]Foni yekutanga yaunayo inonzii?	[Q212O]What is the make of the first phone you own?
[Q212O]Model yefoni yekutanga chii?	[Q212O]What is the model of the first phone you own?
[Q212O] Foni yekutanga iyi smart phone here?	[Q212O] Is the first phone you own a smart phone?
[Q212O] Foni yekutanga iyi wakaitenga gore ripi?	[Q212O] Which year was the first phone you own purchased?
**Phone Yechipiri Yaanayo**	**Phone 2 Owned**
[Q212O]Foni yechipiri yaunayo inonzii?	[Q212O]What is the make of the second phone you own?
[Q212O]Model yefoni yechipiri chii?	[Q212O]What is the model of the second phone you own?
[Q212O] Foni yechipiri iyi smart phone here?	[Q212O] Is the second phone you own a smart phone?
[Q212O] Foni yechipiri iyi wakaitenga gore ripi?	[Q212O]Which year was the second phone you own purchased?
**Phone Yechitatu Yaanayo**	**Phone 3 Owned**
[Q212O]Foni yechitatu yaunayo inonzii?	[Q212O]What is the make of the third phone you own?
[Q212O]Model yefoni yechitatu chii?	[Q212O]What is the model of the third phone you own?
[Q212O] Foni yechitatu iyi smart phone here?	[Q212O]Is the third phone you own a smart phone?
[Q212O] Foni yechitatu iyi wakaitenga gore ripi?	[Q212O]Which year was the third phone you own purchased?
Nhatimbotaurai nezvema cell phone ${Q209bmob} aunokumbidzana nevamwe	Lets talk about the ${Q209bmob} mobile phones you share
**MOBILE PHONE TYPES SHARED**	**MOBILE PHONE TYPES SHARED**

For peer review only

**Phone Yokutanga Yaanokumbidzana kana kushandisa nevamwe**	**Phone 1 Shared**
[Q212S]Foni yekutanga yaunokumbidzana nevamwe inonzii?	[Q212S]What is the make of the first phone you share?
[Q212S]Model yefoni yekutanga chii?	[Q212S]What is the model of the first phone you share?
[Q212S] Foni yekutanga iyi smart phone here?	[Q212S] Is the first phone you share a smart phone?
[Q212S] Foni yekutanga iyi wakaitenga rinhi?	[Q212S] Which year was the first phone you share purchased?
[Q213S] Unoshandisa kana kukumbidzana cell phone yokutanga nani?	[Q213S] Who do you share ownership of your first phone with?
[Q213S] Tsanangudza vamwe vanhu vaunoshandisa phone navo	[Q213S] Specify other
[Q213S] Kakawanda zvakadii kamunowana mukana wekushandisa phone yokutanga?	[Q232S] How often do you have access to the first phone you share?
**Phone Yechipiri Yaanokumbidzana kana kushandisa nevamwe**	**Phone 2 Shared**
[Q212S]Foni yechipiri yaunokumbidzana nevamwe inonzii?	[Q212S]What is the make of the second phone you share?
[Q212S]Model yefoni yechipiri chii?	[Q212S]What is the model of the second phone you share?
[Q212S] Foni yechipiri iyi smart phone here?	[Q212S] Is the second phone you share a smart phone?
[Q212S] Foni yechipiri iyi wakaitenga rinhi?	[Q212S]Which year was the second phone you share purchased?
[Q213S] Unoshandisa kana kukumbidzana cell phone yechipiri nani?	[Q213S] Who do you share ownership of your second phone with?
[Q213S] Tsanangudza vamwe vanhu vaunoshandisa phone navo	[Q213C] Specify other
[Q213S] Kakawanda zvakadii kamunowana mukana wekushandisa phone yechipiri?	[Q232S] How often do you have access to the second phone you share?
**Phone Yechitatu Yaanokumbidzana kana kushandisa nevamwe**	**Phone 3 Shared**
[Q212S]Foni yechitatu yaunokumbidzana nevamwe inonzii?	[Q212S]What is the make of the third phone you share?
[Q212S]Model yefoni yechitatu chii?	[Q212S]What is the model of the third phone you share?
[Q212S] Foni yechitatu iyi smart phone here?	[Q212S]Is the third phone you share a smart phone?
[Q212S] Foni yechitatu iyi wakaitenga rinhi?	[Q212S]Which year was the third phone you share purchased?
[Q213S] Unoshandisa kana kukumbidzana cell phone yechitatu nani?	[Q213S] Who do you share ownership of your third phone with?
[Q213S] Tsanangudza vamwe vanhu vaunoshandisa phone navo	[Q213C] Specify other
[Q213S] Kakawanda zvakadii kamunowana mukana wekushandisa phone yechitatu?	[Q232S] How often do you have access to the third phone you share?
**ZVEVANE PHONE KANA KUWANA MUKANA WE PHONE**	**FOR THOSE WITH PHONES OR ACCESS**
**MHANDO DZEMALINE DZINOSHANDISWA**	**MOBILE PROVIDERS USED**
[Q214prov] Unowanzoshandisa line ripi?	[Q214prov] Which mobile provider do you use normally?
[Q215]Unoshandisawo futi Econet	[Q215] Also use Econet network
[Q215] Unoshandisawo futi Telecel	[Q215] Also use Telecel network
[Q215] Unoshandisawo futi Netone	[Q215] Also use Netone network
[Q215]Unoshandisawo futi Africom	[Q215] Also use Africom network
**MARI DZINOSHANDISWA PA MOBILE PHONE**	**EXPENDITURE ON MOBILE PHONE**

[Q216air] Mari yekufonesa pa cell phone inogarotengwa here kana kuti inotengwa pamwedzi pamwedzi (contract)?	[Q216 air]For your phone, does airtime need to be purchased in advance or is it paid monthly through a contract?
[Q217amt] Pasvondo rapfuura, wakashandisa mari inosvika madhora mangani pa airtime?	[Q217amt] How much airtime, in dollars, did you use in the past week?
[Q218exp] Pasvondo rapfuura, wakashandisa mari inosvika madhora mangani pane zvimwe zvinhu zvenyu tisingasanganise ne airtime?	[Q218exp] How much money did you spend on other things for yourself in the past week excluding airtime for cell phones?
**NDIANI ANOWANZOBHADHARA MARI DZINOSHANDISWA PA MOBILE PHONE**	**WHO USUALLY PAYS FOR EXPENDITURE ON MOBILE PHONE**
[Q219]Ndini	[Q219]I do
[Q219]Vabereki/ Vanondichengeta	[Q219]My parents/ legal guardians
[Q219]Dzimwe Hama	[Q219]Other relative
[Q219]Shamwari	[Q219]Friend
[Q219]Mudiwa diwa	[Q219]Boyfriend/girlfriend
[Q219]Handishandise mari paairtime	[Q219]I don't spend money on airtime
[Q219]Vamwewo (Tsanangudza pazasi)	[Q219]Other (specify below)
[Q219_97]Handizivi	[Q219_97]Don't know
[Q219_99]Hapana	[Q219_99]No response
[Q219_other] Tsanangudza vamwe vano badhara	[Q219_other] specify Other person who pays for credit
[Q220_age] Wakatanga kushandisa cell phone une makore mangani?	[Q220_age] At what age did you start using a mobile phone?
**MIBVUNZO YEVANE PHONE CHETE**	**ONLY FOR THOSE WHO OWN A PHONE**
[Q221]Wakawana cell phone yako yekutanga sei?	[Q221] How did you get your first phone?
[Q221] Tsanangudza zvimwe zvakaita kuti uve nephone yekutanga	[Q221] Specify other reason why you got your first phone
[Q222] Akatenga cell phone yako yekutanga ndiyani?	[Q222]Who bought your first phone?
[Q222] Tsanangura mumwe akatenga phone	[Q222] Specify other person who bought first phone
**Inguva dzipi dzinowanzo kunge dzakadzima cell phone yako?**	**When is your phone usually switched off**
[Q223]Hapana pandinodzima	[Q223] Never
[Q223]Pandinenge ndakavata	[Q223]When I am sleeping
[Q223]Pandinenge ndiri kuchikoro/kubasa	[Q223]When I am at school/ work
[Q223]Kana ndisisina mari yekufonesa	[Q223]When I have run out of credit
[Q223]Kana bhatiri rava nemoto mushoma	[Q223]When the battery is low
[Q223]Dzimwewo nguva (tsanangurai pazasi)	[Q223]Other (specify below)
[Q223]Handizivi	[Q223]Don't know
[Q223]Hapana minduro	[Q223]No response
[Q223] Tsanangurai dzimwe nguva dzinodzimwa phone	[Q223]Specify other time phone is switched off
[Q224] Kakawanda zvakadii kamunoita kuti cell phone yenyu isarire zvinonzwika ('SILENT' or 'VIBRATE')?	[Q224] How often do you place your phone on 'SILENT' or 'VIBRATE' mode?
[Q225]Unotakura cell phone yako kuchikoro here?	[Q225] Do you take your cell phone to school ?
[Q226] Unogona kugara usina cell phone kwezuva rese here?	[Q226] Can you do without a mobile for a day?
[Q227] Kana ati kwete, nemhaka yei?	[Q227] Why cannot you not do without a phone
[Q228] Kwamuri chii chakanakira kuita cell phone?	[Q228] According to you, the best part of having a mobile phone is
[Q228] Tsanangurai zvimwe zvakanakira kuita cell phone?	[Q228] What are the other best part of having a phone
**Vasina Phone**	**For those with No Phone**
[Q229] Kana usina cell phone, nemhaka yei musina?	[Q229] If you don't have a mobile phone, kindly tell me why not?

[Q229] Zwimwe zvinoita kuti musave ne phone	[Q229] Whats the other reason for not having a phone
[Q235] Mune hurongwa here hwekutenga cell phone munguva inotevera?	[Q235] Are you planning to buy a mobile phone in near future?
[Q236] Kana mati hongu, riinhi?	[Q236] When are you planning to buy a mobile phone?
**Unoshandisa cell phone zvakadini kana uchiita izvi:**	**How frequently do you use a mobile phone for:**
[Q237]Kufona	[Q237]Phone calls
[Q237]Kutumira ma message kana pictures (kwete ne app)	[Q237]Sending text or picture messages (not via an app)
[Q237]Kutumira ma message kana chat (pa Whatsapp, Facebook)	[Q237]Sending instant messages or chat services (e.g Whatsapp, Facebook etc.)
[Q237]Kutamba ma games	[Q237]Playing games
[Q237]Kuona TV/movies/videos	[Q237]Watch TV/movies/videos
[Q237Kuterera kana ku downloader music/radio	[Q237]Listen to/ download music/radio
[Q237]Kunzwa news dzemitambo kana zvimwe	[Q237]Getting news updates (sports or other)
[Q237]Calculator	[Q237]Calculator
[Q237]]Social network yakafanana ne twitter, Instagram, Facebook	[Q237]]Social networking sites e.g. twitter, Instagram, Facebook
[Q237]Kubhadhara kana kutengesa zvinhu	[Q237]Making payments/ purchasing goods
**Unoshandisa cell phone zvakadini kana uchiita izvi:**	**How frequently do you use a mobile phone for:**
[Q237]Camera	[Q237]Camera
[Q237]Kufamba kwako nema maps	[Q237]Navigating with maps
[Q237]E-mailing	[Q237]E-mailing
[Q237]Uchitsvaga ruzivo rwezvekuchikoro uchiita homework kana zvekubasa	[Q237]Research for school/work or doing homework /revision e.g. Ruzivo
[Q237]Uchitsvaga ruzivo rwezveutano	[Q237]Search for information about my health
[Q237]Uchitsvaga ruzivo rwezvekudanana ne zvepabonde	[Q237]Search for information about relationships and sex
[Q237]Calendar	[Q237]Calendar
[Q237]Clock	[Q237]Clock
[Q237]Dictionary	[Q237]Dictionary
[Q237]Bible	[Q237]Bible
**Ndeapi ma apps matatu aunowanzoshandisa?**	**What are the three apps that you most often use ?**
[Q238_1]Sarudza app yokutanga	[Q238_1]Select the first app
[Q238_1]Tsanagudza rimwe zita	[Q238_1]Specify other
[Q238_2] Sarudza app yechipiri	[Q238_2]Select the second app
[Q238_2]Tsanagudza rimwe zita	[Q238_2]Specify other
[Q238_3]Sarudza app yechitatu	[Q238_3]Select the third app
[Q238_3]Tsanagudza rimwe zita	[Q238_3]Specify other
[Q239] Unombotamba ma games pa cell phone?	[Q239] Do you play any games on a cell phone?
**Ndeapi ma games matatu aunowanzotamba pa cell phone?**	**What are the three games you most often play on a cell phone?**
[Q239] Zita re Game rokutanga	[Q239] Name Game 1
[Q239] Tsanangudza Zita re Game rokutanga	[Q239] Specify Name Game 1
[Q239] Zita re Game rechipiri	[Q239] Name Game 2
[Q239] Tsanangudza Zita re Game rechipiri	[Q239] Specify Name Game 2
[Q239] Zita re Game rechitatu	[Q239] Name Game 3
[Q239] Tsanangudza Zita re Game rechitatu	[Q239] Specify Name Game 3
[Q240]Wakamboshandisa phone kuti uone kuti utano hwako hurikufamba sei?	[Q240] Have you ever used your phone for tracking your health?
[Q241]Nditaurire ma apps aunoshandisa kuona kuti utano hwako hwakamira sei	[Q241]Please list the applications that you use to track your personal health measures

For peer review only

ZVAKAVANZIKA kana PRIVATE Dzako	PRIVACY
[Q242]Unofunga kuti zvaunotumira paphone zviri 'private' kana zvakavanzika here?	[Q242]How 'private' do you consider the information that you send when using a phone?
[Q243]Unofunga kuti zvaunotumirwa paphone zviri 'private' kana zvakavanzika here?	[Q243]How 'private' do you consider the information that you receive when using a phone?
[Q244]Unofunga kuti zviri paphone zviri 'private' kana zvakavanzika here?	[Q244]How 'private' do you consider the information stored in your mobile phone?
[Q245] Une mapasswords yeku Locker phone yako here?	[Q245] Do you have passwords to lock/unlock your phone?
[Q246]Mune mapasswords eApplications ipi zvayo pacell phone yako?	[Q246]Do you have passwords for any applications on your phone?
[Q246b]Kana wati hongu, ndeapi maApplications anema passwords??	[Q246b]If yes, which ones?
**SECTION 3 PFUNGWA DZAKO - SSQ**	**SECTION 3 MENTAL HEALTH SSQ**
Iye zvinondichakubvunzai kuti mainzwa sei muvhiki rapfuura. Ndapota pindurai hongu kana kwete pamubvunzo umwe neumwe	Now I am going to ask you how you have been feeling over the past week. Please respond yes or no to each question
Muvhiki rapfuura	**In the past week**
[Q301]Pane pandaimboona ndichinyanya kufungisisa kana kufunga zvakawanda	[Q301]There were times in which I was thinking deeply or thinking about many things
[Q302]Pane pandaimbotadza kuisa pfungwa dzangu pamwechete	[Q302]I found myself sometimes failing to concentrate
[Q303]Pane pandaimboshatirwa kana kuita hasha zvenhando	[Q303]I lost my temper or got annoyed over trivial matters
[Q304]Pane pandaimborota hope dzinotyisa kana dzisina kunaka	[Q304]I had nightmares or bad dreams
[Q305]Pane pandaimboona kana kunzwa zvinhu zvaisaonekwa kana kunzwikwa nevamwe	[Q305]I sometimes saw or heard things which others could not see or hear
[Q306]Pane pandaimborwadziwa nemudumbu	[Q306]My stomach was aching
Muvhiki rapfuura	**In the past week**
[Q307]Pane pandaimbovhundutswa nezvinhu zvisina mature	[Q307]I was frightened by trivial things
[Q308]Pane pandaimbotadza kurara kana kushaya hope	[Q308]I sometimes failed to sleep or lost sleep
[Q309]Pane pandaimbonzwa kuomerwa neupenyu zvekuti ndaimbochema kana kunzwa kuda kuchema	[Q309]There were moments when I felt life was so tough that I cried or wanted to cry
[Q310]Pane pandaimbonzwa kuneta (kurukutika)	[Q310]I felt run down (tired)
[Q311]Pane pandaimboita pfungwa dzekuda kuzviuraya	[Q311]At times I felt like committing suicide
[Q312]Ndainzwa kusafara nezvinhu zvandaiita zuva nezuva	[Q312]I was generally unhappy with things that I would be doing each day
[Q313]Basa rangu rava kurasarira mumashure	[Q313]My work was lagging behind
[Q314]Ndainzwa zvichindiomera kuti ndoita zvipi	[Q314]I felt I had problems in deciding what to do
**PFUNGWA DZAKO - SDQ**	**MENTAL HEALTH AND WELL-BEING SDQ**
**Ndinokumbira kuti upe mhinduro dzako wakatarisa zvaiitika kana mamiriro epfungwa dzako mwedzi mitanhatu yapfuura.**	Please give your answers based on how things have been for you over the last six months.
**MUMWEDZI MITANHATU YAPFUURA NDAINZWA KANA KUFUNGA IZVI**	**MENTAL HEALTH AND WELL-BEING SDQ in the last six months**
[Q401]Ndinoyedza kuve munhu akanaka kune vamwe vanhu. Ndinoda kuita zvinoita kuti vafare	[Q401]I try to be nice to other people. I care about their feelings.
[Q402]Handigadzikane, handigoni kugara panzvimbo nguva yakareba	[Q402]I am restless, I cannot stay still for long
[Q403]Ndinogara ndichinzwa musoro, mudumbu nekungorwara rwara	[Q403]I get a lot of headaches, stomach-aches or sickness

For peer review only

[Q404]Nguva zhinji ndinogovana nevamwe(zvekudya, zvekutambisa, zvinyoreso nezvimwe)	[Q404]I usually share with others (food, games, pens etc.)
[Q405]Ndinotsamwa zvekutsamwa zviya zvakare ndinokurumidza kushatirwa	[Q405]I get very angry and often lose my temper
[Q406]Nguva zhinji ndinenge ndiri ndega. Ndinotamba ndega kana kugara ndega	[Q406]I am usually on my own. I generally play alone or keep to myself
[Q407]Ndinowanzoita zvandinenge ndaudzwa	[Q407]I usually do as I am told
[Q408]Ndinofunganya zvakanyanya	[Q408]I worry a lot
**MUMWEDZI MITANHATU YAPFUURA NDAINZWA KANA KUFUNGA IZVI**	**MENTAL HEALTH AND WELL-BEING SDQ in the last six months**
[Q409]Ndinobatsira kana mumwe munhu arwadziswa kana asiri kunzwa zvakanaka kana ashatiriswa	[Q409]I am helpful if someone is hurt, upset or feeling ill
[Q410]Ndinogara ndichingoshanyarika ndisingagadzikane	[Q410]I am constantly fidgeting or squirming
[Q411]Ndine shamwari imwechete kana dzakawanda	[Q411]I have one good friend or more
[Q412]Ndinorwa zvakanyanya .Ndinogona kuti vamwe vanhu vaite zvandinoda	[Q412]I fight a lot. I can make other people do what I want.
[Q413]Nguva zhinji ndinenge ndakasuwa, moyo wangu uchirwadza kana kuda kuchema	[Q413]I am often unhappy, down-hearted or tearful
[Q414]Vamwe vanhu vezera rangu vanondifarira	[Q414]Other people my age generally like me
[Q415]Ndinokurumidza kuita charinga, ndinotadza kunyatsoita sezvandinotarisirwa	[Q415]I am easily distracted, I find it difficult to concentrate
[Q416]Ndinonzwa kutya munzvimbo itsva. Ndinokurumidza kushaya chivimbo nezvandinenge ndichiita	[Q416]I am nervous in new situations. I easily lose confidence.
[Q417]Ndine tsitsi kuvanhu vechidiki	[Q417]I am kind to younger children.
[Q418]Ndinogara ndichipomerwa kunyepa nekusataura chokwadi	[Q418]I am often accused of lying or cheating
**MUMWEDZI MITANHATU YAPFUURA NDAINZWA KANA KUFUNGA IZVI**	**MENTAL HEALTH AND WELL-BEING SDQ in the last six months**
[Q419]Vamwe vana vanondisvora vachindichisidzira nekundirova kana kundituka kana kungoita zvinondidzikisira	[Q419]Other children or young people pick on me or bully me
[Q420]Kazhinji ndinobatsira vamwe(vabereki, vadzidzisi nevana)	[Q420]I often volunteer to help others (parents, teachers, children)
[Q421]Ndinonyatsofunga ndisati ndaita zvinhu	[Q421]I think before I do things
[Q422]Ndinotora zvinhu zvisiri zvangu kubva kumba, kuchikoro kana kumwewo	[Q422]I take things that are not mine from home, school or elsewhere
[Q423]Ndinowirirana zvakanaka nevanhu vakuru panevezera rangu	[Q423]I get on better with adults than with people my own age
[Q424]Ndine zvandinotya, ndinokurumidza kuvhunduka/kutya	[Q424]I have many fears, I am easily scared
[Q425]Ndinopedza basa randinenge ndichiita. Ndinonyatsoita sezvinotarisirwa	[Q425]I finish the work I'm doing. My attention is good

For peer review only

Table S1: Shona symptom questionnaire (SSQ): item response rates by questionnaire version used (n=634)

	Total [a]	Version 1	Version 2	p-value for age-adjusted difference between versions [b]
n	634	123	511	
Median SSQ (IQR)	7 (4, 9)	7 (4,9)	7 (4,9)	
Mean SSQ (95% CI)	6.44 (6.13, 6.74)	6.36 (5.60, 7.12)	6.46 (6.13, 6.79)	p=0.826
At risk for CMD (SSQ>=8)	37.38 (32.99, 41.99)	41.46 (32.40, 51.14)	36.40 (31.48, 41.62)	p=0.300
At risk for CMD (SSQ>=9)	28.55 (24.88, 32.53)	31.71 (24.33, 40.14)	27.79 (23.66, 32.33)	p=0.346
Severely at risk of CMD (SSQ>=11)	11.51 (9.08, 14.51)	13.82 (8.10, 22.59)	10.96 (8.42, 14.15)	p=0.410
I sometimes saw or heard things which others could not see or hear	9.78 (7.47, 12.71)	12.20 (7.07, 20.24)	9.20 (6.78, 12.36)	p=0.369
At times I felt like committing suicide	11.20 (9.01, 13.84)	14.63 (9.13, 22.64)	10.37 (8.10, 13.20)	p=0.166
Red flag	22.08 (18.55, 26.07)	27.64 (19.02, 38.32)	20.74 (17.04, 25.02)	p=0.160

a. mean and proportions taking into account clustered study design but not adjusted for age
b. Adjusted Wald test accounting for clustered study design and adjusting for age in years

For peer review only

Table S2 SSQ – item response rates by sex and overall response (yes, no) (n=634)

Shona Symptom Questionnaire items		Males		Females		Total
	N		N		N	
There were times in which I was thinking deeply or thinking about many things	153	64.56%	253	63.73%	406	64.04%
I found myself sometimes failing to concentrate	145	61.18%	225	56.68%	370	58.36%
I lost my temper or got annoyed over trivial matters	129	54.4%	237	59.7%	366	57.7%
I had nightmares or bad dreams	143	60.3%	257	64.7%	400	63.1%
I sometimes saw or heard things which others could not see or hear	34	14.4%	28	7.1%	62	**9.8%**
My stomach was aching	146	61.6%	273	68.8%	419	66.1%
I was frightened by trivial things	88	37.1%	150	37.8%	238	37.5%
I sometimes failed to sleep or lost sleep	119	50.2%	211	53.2%	330	52.1%

There were moments when I felt life was so tough that I cried or wanted to cry	116	49.0%	227	57.2%	343	54.1%
I felt run down (tired)	127	53.6%	242	61.0%	369	58.2%
At times I felt like committing suicide	17	7.2%	54	13.6%	71	**11.2%**
I was generally unhappy with things that I would be doing each day	91	38.4%	110	27.7%	201	31.7%
My work was lagging behind	85	35.9%	97	24.4%	182	28.7%
I felt I had problems in deciding what to do	117	49.4%	207	52.1%	324	51.1%

For peer review only

Table S3: Strength and Difficulties Questionnaire (SDQ): item response rates by sex and overall response (n=634)

**Mean SDQ scores**	All (n=634)			Males (n=237)			Females (n=397)		
	**Mean**	S.D.	Alpha	**Mean**	S.D.	Alpha	**Mean**	S.D.	Alpha
Difficulties total	**10.74**	6.25	0.75	**10.72**	6.23	0.74	**10.75**	6.28	0.75
Externalising score	**4.31**	3.48	0.68	**4.65**	3.59	0.67	**4.10**	3.41	0.69
Internalising score	**6.43**	3.94	0.63	**6.07**	3.69	0.59	**6.64**	4.08	0.64
Emotional symptoms	**3.66**	2.66	0.60	**3.38**	2.56	0.59	**3.83**	2.71	0.61
Conduct problems	**2.22**	1.97	0.46	**2.24**	2.09	0.49	**2.20**	1.89	0.46
Hyperactivity/inattention	**2.09**	2.03	0.52	**2.41**	2.07	0.50	**1.90**	1.99	0.54
Peer problems	**2.77**	2.07	0.38	**2.69**	1.94	0.33	**2.81**	2.15	0.41
Prosocial behaviour	**8.13**	2.22	0.68	**8.14**	2.24	0.69	**8.12**	2.20	0.67

Range of all scales 0-10, except Difficulties Total which ranges 0-40

For peer review only

Table S4 Mean SDQ scores by sex with adjustment for age and cluster sampling design

Mean SDQ scores	All (n=634)		Males (n=237)		Females (n=397)		Difference Wald		
	Mean	95% CI	Mean	95% CI	Mean	95% CI	F	d.f.	P
Difficulties total	**10.74**	6.25	**10.71**	9.84 -11.58	**10.75**	10.12-11.38	0.01	1,123	0.9379
Externalising score	**4.31**	3.48	**4.63**	4.14-5.13	**4.11**	3.79-4.44	3.86	1,123	0.0517
Internalising score	**6.43**	3.94	**6.08**	5.58-6.57	**6.64**	6.23-7.05	3.02	1,123	0.0848

For peer review only - http://bmjopen.bmj.com/site/about/guidelines.xhtml

STROBE 2007 (v4) Statement—Checklist of items that should be included in reports of *cross-sectional studies*

Section/Topic	Item #	Recommendation	Reported on page #
**Title and abstract**	1	(*a*) Indicate the study's design with a commonly used term in the title or the abstract	3
		(*b*) Provide in the abstract an informative and balanced summary of what was done and what was found	3
**Introduction**			
Background/rationale	2	Explain the scientific background and rationale for the investigation being reported	4
Objectives	3	State specific objectives, including any prespecified hypotheses	4
**Methods**			
Study design	4	Present key elements of study design early in the paper	4
Setting	5	Describe the setting, locations, and relevant dates, including periods of recruitment, exposure, follow-up, and data collection	4
Participants	6	(*a*) Give the eligibility criteria, and the sources and methods of selection of participants	4
Variables	7	Clearly define all outcomes, exposures, predictors, potential confounders, and effect modifiers. Give diagnostic criteria, if applicable	5
Data sources/ measurement	8*	For each variable of interest, give sources of data and details of methods of assessment (measurement). Describe comparability of assessment methods if there is more than one group	5
Bias	9	Describe any efforts to address potential sources of bias	*5*
Study size	10	Explain how the study size was arrived at	5
Quantitative variables	11	Explain how quantitative variables were handled in the analyses. If applicable, describe which groupings were chosen and why	5-6
Statistical methods	12	(*a*) Describe all statistical methods, including those used to control for confounding	5-6
		(*b*) Describe any methods used to examine subgroups and interactions	
		(*c*) Explain how missing data were addressed	
		(*d*) If applicable, describe analytical methods taking account of sampling strategy	
		(*e*) Describe any sensitivity analyses	
**Results**			

For peer review only

Participants	13*	(a) Report numbers of individuals at each stage of study—eg numbers potentially eligible, examined for eligibility, confirmed eligible, included in the study, completing follow-up, and analysed	7
		(b) Give reasons for non-participation at each stage	7
		(c) Consider use of a flow diagram	Figure 1
Descriptive data	14*	(a) Give characteristics of study participants (eg demographic, clinical, social) and information on exposures and potential confounders	7
		(b) Indicate number of participants with missing data for each variable of interest	7
Outcome data	15*	Report numbers of outcome events or summary measures	7-9
Main results	16	(*a*) Give unadjusted estimates and, if applicable, confounder-adjusted estimates and their precision (eg, 95% confidence interval). Make clear which confounders were adjusted for and why they were included	7-14 and Tables 1-4
		(*b*) Report category boundaries when continuous variables were categorized	
		(*c*) If relevant, consider translating estimates of relative risk into absolute risk for a meaningful time period	
Other analyses	17	Report other analyses done—eg analyses of subgroups and interactions, and sensitivity analyses	Supplementary material
**Discussion**			
Key results	18	Summarise key results with reference to study objectives	14
Limitations	19	Discuss limitations of the study, taking into account sources of potential bias or imprecision. Discuss both direction and magnitude of any potential bias	16
Interpretation	20	Give a cautious overall interpretation of results considering objectives, limitations, multiplicity of analyses, results from similar studies, and other relevant evidence	15-16
Generalisability	21	Discuss the generalisability (external validity) of the study results	15
**Other information**			
Funding	22	Give the source of funding and the role of the funders for the present study and, if applicable, for the original study on which the present article is based	17

*Give information separately for cases and controls in case-control studies and, if applicable, for exposed and unexposed groups in cohort and cross-sectional studies.

Note: An Explanation and Elaboration article discusses each checklist item and gives methodological background and published examples of transparent reporting. The STROBE checklist is best used in conjunction with this article (freely available on the Web sites of PLoS Medicine at http://www.plosmedicine.org/, Annals of Internal Medicine at http://www.annals.org/, and Epidemiology at http://www.epidem.com/). Information on the STROBE Initiative is available at www.strobe-statement.org.

For peer review only

BMJ Open

Common mental health and emotional and behavioural disorders among adolescents and young adults in Harare and Mashonaland East, Zimbabwe: A population-based prevalence study

Journal:	*BMJ Open*
Manuscript ID	bmjopen-2022-065276.R1
Article Type:	Original research
Date Submitted by the Author:	08-Dec-2022
Complete List of Authors:	Doyle, Aoife; London School of Hygiene & Tropical Medicine; Biomedical Research and Training Institute<br>Bandason, T; Biomedical Research and Training Institute<br>Dauya, E; Biomedical Research and Training Institute<br>McHugh, Grace; Biomedical Research and Training Institute<br>Grundy, Chris; London School of Hygiene & Tropical Medicine<br>Simms, Victoria; London School of Hygiene & Tropical Medicine; Biomedical Research and Training Institute<br>Chibanda, D; College of Health Sciences University of Zimbabwe, Department of Psychiatry; London School of Hygiene & Tropical Medicine<br>Ferrand, Rashida; Biomedical Research and Training Institute; London School of Hygiene & Tropical Medicine, Department of Clinical Research
**Primary Subject Heading**:	Epidemiology
Secondary Subject Heading:	Global health, Mental health, Public health
Keywords:	MENTAL HEALTH, Child & adolescent psychiatry < PSYCHIATRY, EPIDEMIOLOGY

SCHOLARONE™
Manuscripts

I, the Submitting Author has the right to grant and does grant on behalf of all authors of the Work (as defined in the below author licence), an exclusive licence and/or a non-exclusive licence for contributions from authors who are: i) UK Crown employees; ii) where BMJ has agreed a CC-BY licence shall apply, and/or iii) in accordance with the terms applicable for US Federal Government officers or employees acting as part of their official duties; on a worldwide, perpetual, irrevocable, royalty-free basis to BMJ Publishing Group Ltd ("BMJ") its licensees and where the relevant Journal is co-owned by BMJ to the co-owners of the Journal, to publish the Work in this journal and any other BMJ products and to exploit all rights, as set out in our licence.

The Submitting Author accepts and understands that any supply made under these terms is made by BMJ to the Submitting Author unless you are acting as an employee on behalf of your employer or a postgraduate student of an affiliated institution which is paying any applicable article publishing charge ("APC") for Open Access articles. Where the Submitting Author wishes to make the Work available on an Open Access basis (and intends to pay the relevant APC), the terms of reuse of such Open Access shall be governed by a Creative Commons licence – details of these licences and which Creative Commons licence will apply to this Work are set out in our licence referred to above.

Other than as permitted in any relevant BMJ Author's Self Archiving Policies, I confirm this Work has not been accepted for publication elsewhere, is not being considered for publication elsewhere and does not duplicate material already published. I confirm all authors consent to publication of this Work and authorise the granting of this licence.

For peer review only

Common mental health and emotional and behavioural disorders among adolescents and young adults in Harare and Mashonaland East, Zimbabwe: A population-based prevalence study

Authors:
Doyle AM[1,2], Bandason T[2], Dauya, E[2], McHugh G[2], Grundy C[1], V Simms[1, 2], Chibanda D[3,4], Ferrand, RA[2,5]

Affiliations:
1 Medical Research Council International Statistics and Epidemiology Group, Faculty of Epidemiology and Population Health, London School of Hygiene & Tropical Medicine, London, UK

2 The Health Research Unit Zim (THRU ZIM), Biomedical Research and Training Institute, Zimbabwe

3 Department of Psychiatry, College of Health Sciences, University of Zimbabwe, Harare, Zimbabwe.

4 Centre for Global Mental Health, London School of Hygiene & Tropical Medicine, London, UK
5 Department of Clinical Research, London School of Hygiene & Tropical Medicine, London, UK

Corresponding Author:

Dr. Aoife Doyle

Medical Research Council International Statistics and Epidemiology Group

London School of Hygiene & Tropical Medicine

London WC1E7HT, UK

+447967497294

Aoife.doyle@lshtm.ac.uk

Ms Tsitsi Bandason

The Health Research Unit Zim (THRU ZIM)

Biomedical Research and Training Institute

Harare, Zimbabwe

vakoma@gmail.com

Ms Ethel Dauya

The Health Research Unit Zim (THRU ZIM)

For peer review only - http://bmjopen.bmj.com/site/about/guidelines.xhtml

Biomedical Research and Training Institute

Harare, Zimbabwe

edauya@brti.co.zw

Dr Grace Mc Hugh

The Health Research Unit Zim (THRU ZIM)

Biomedical Research and Training Institute

Harare, Zimbabwe

graceandandre@gmail.com

Dr Chris Grundy

Medical Research Council International Statistics and Epidemiology Group

London School of Hygiene & Tropical Medicine

London WC1E7HT, UK

Chris.grundy@lshtm.ac.uk

Dr Victoria Simms

Medical Research Council International Statistics and Epidemiology Group

London School of Hygiene & Tropical Medicine

London WC1E7HT, UK

Victoria.simms@lshtm.ac.uk

Dr Dixon Chibanda

Centre for Global Mental Health

London School of Hygiene & Tropical Medicine

London WC1E7HT, UK

Dixon.Chibanda@lshtm.ac.uk

Prof Rashida Ferrand

Department of Clinical Research

London School of Hygiene & Tropical Medicine

For peer review only

London WC1E7HT, UK

Rashida.ferrand@lshtm.ac.uk

Word count- ~4000 (4000 max for BMJ Open)

For peer review only

For peer review only - http://bmjopen.bmj.com/site/about/guidelines.xhtml

Abstract (300 words max): current 287

Objectives To estimate the prevalence of common mental health disorders (CMDs) and emotional and behavioural disorders among young people and to explore the correlates of CMDs risk.

Setting Five urban and peri-urban communities in Harare and Mashonaland East, Zimbabwe

Design Population-based cross-sectional study

Participants Young people aged 13-24 years living in households in the study areas

Outcome measures The primary outcome was the proportion of participants screening positive for probable CMDs defined as a Shona Symptoms Questionnaire (SSQ) score ≥8. Secondary outcomes were emotional and behavioural disorders measured using the Strength and Difficulties Questionnaire (SDQ), and adjusted odds ratios for factors associated with CMD.

Results Out of 634 young people, 37.4% (95% CI 33.0%, 42.0%) screened positive for probable CMDs, 9.8% (95% CI 7.5%, 12.7%) reported perceptual symptoms, and 11.2% (95%CI 9.0%, 13.8%) reported suicidal ideation. Using UK norms to define normal, borderline and abnormal scores for each of the SDQ domains, a high proportion (15.8%) of Zimbabwean young people had abnormal scores for emotional symptoms, and a low proportion had abnormal scores for hyperactivity/inattention scores (2.8%) and prosocial scores (7.1%). We created local cut-offs for the emotional symptoms, hyperactivity/attention, and prosocial SDQ domains. The odds of probable CMDs increased with each year of age (OR 1.09, $p<0.001$) and was higher among those who were out of school and not working compared to those in school or working (adj OR 1.67 (1.07, 2.62), $p=0.04$). One in five participants (22.1%) were referred immediately for further clinical assessment but uptake of referral services was low.

Conclusions We observed a high prevalence of symptoms of CMDs among general population urban and peri-urban young people especially among those with no employment. There is a need for more accessible and acceptable youth-friendly mental health services.

Strengths and limitations of this study

- In the context of limited population-level data on the mental health of young people, we included a representative general population sample of young people in five urban and peri-urban communities in Zimbabwe
- Demonstration of the feasibility of use of audio-computer assisted self-interview (ACASI) to collect self-reported measures of mental health and emotional and behavioural disorders in this young population.
- The SSQ and SDQ tools have not been extensively validated for use in the full age group and the translation of the SDQ into Shona did not follow recommended procedures. Additional validation studies are needed for tools to measure adolescent mental health.

For peer review only - http://bmjopen.bmj.com/site/about/guidelines.xhtml

Keywords:

*Adolescent; Young Adult; Cross-Sectional Studies; Mental health; Zimbabwe

Background:

Mental health disorders such as depressive, anxiety and childhood behavioural disorders are common among young people globally,[1] and in 2019 were among the top causes of morbidity among 10-19 year olds.[2] Poor mental health among adolescents is associated with increased risky behaviours,[3, 4] can impact on the management of other chronic health conditions, such as adherence to HIV treatment,[4-6] and can adversely affect educational and employment achievements.

Despite an increased focus on mental health programming and services, the global burden of mental health conditions is estimated to have remained relatively constant between 1990 and 2019.[1] In many low-and middle income settings, there are considerable treatment gaps including a lack of services and/or adequately trained staff.[7] Also, young people may not be aware of the available mental health services or find it difficult to attend health facilities as they are at school or at work. Additional barriers include stigma around mental health conditions and young people's mistrust in service provision.

Understanding the epidemiology of mental health disorders in young people is essential to inform appropriate service provision and intervention development, and to inform policies and resource allocation. One of the challenges facing adolescent mental health programming for young people globally are gaps in data on the prevalence and determinants of disorders.[8, 9] In Zimbabwe, there are limited population-level data on the mental health of young people. A 2006 population-based survey on mental health disorders among rural 15- 23 year olds found 52% to be at risk of being affected and 24% to be at risk of being severely affected of common mental health disorders.[3] A 2009-2011 survey found a prevalence of psychological distress of 4.5% among males and 8.2% of females aged 15-24 years.[4] More recent published data on adolescent mental health in Zimbabwe is primarily from studies in adolescents living with HIV. For example, in 2016/17, 20.4% of adolescents living with HIV recruited into a trial at public clinics in two rural districts were at risk of CMDs.[10]

The primary aim of this study was to estimate the prevalence of common mental health disorders (CMDs) among young people. Secondary aims were to estimate the prevalence of emotional and behavioural disorders and to explore factors associated with CMDs.

Methods:

A cross-sectional population-based survey was conducted in 2018 among young people (13-24 years) in five communities in urban and peri-urban Harare and Mashonaland East, Zimbabwe. The communities were participating in a cluster randomised trial of a community-based, multi-component HIV and sexual and reproductive health intervention for young people (the CHIEDZA trial). The aim of the survey was to describe young people's access to and use of technology including mobile phones, and to describe their mental health and well-being. The mobile phone and technology use results have been reported separately.[11] The survey was undertaken as part of formative research to inform the trial intervention, and the findings informed the development of the technology and general health information and counselling components of the intervention. The survey communities were purposively selected to represent both urban and peri-urban communities. The trial protocol has been published,[12] and randomisation and trial implementation occurred after this survey was completed.

Eligible participants were aged 13 to 24 years, resident in the study community at the time of the survey, and either provided informed consent (16-24 years) or provided assent with guardian consent

For peer review only - http://bmjopen.bmj.com/site/about/guidelines.xhtml

(age 13-15 years). A simple random sample of 100 GPS coordinates (primary sampling unit) were sampled per cluster from all potential points in the study areas using ArcGIS software version 10.5 (Esri, Redlands, USA). Points were randomly ordered and then sequentially visited by a team of interviewers. All households with front doors within 20 meters of the sampled GPS point were visited. The household head was interviewed to obtain basic demographic information about the household and was asked for consent to interview any eligible YP. If the household head was not available, another household member aged 16+ years or a neighbour was asked to provide information on the composition of the household to determine potential household eligibility. Households with young people were visited a further two times in order to interview the household head. All young people in the selected households were eligible for recruitment. The target sample size of 686 would provide +/-6% precision around an expected prevalence of 50% or participants owning a mobile phone and assuming a design effect of 2 and 10% non-response.

Data collection

Participants responded to a short (approximately 30 minute) audio computer-assisted self-interviewing (ACASI) tablet-based questionnaire **(Supplementary material)**. Questions were adapted from pre-existing questionnaires.[13-16] The questionnaire was developed in English and translated into Shona (the local language) and participants could respond in English or Shona. Modifications were made to the questionnaire following pretesting with the study team and following the pilot survey which was conducted outside the selected study sites.

The Shona symptom questionnaire (SSQ) is a locally-validated 14 item scale asking about symptoms of common mental health disorders in the past week with dichotomised yes/no response categories.[17] Two different versions of the SSQ were used. For piloting and the first two weeks of data collection (~20% of data collection), we used a Shona version that had been created by the study team through translation of the English version of the SSQ into Shona so that it would be understandable by young people. For the remainder of the survey a self-completed version of the Shona SSQ which had previously been validated among 15-24 year olds was used.[18] A comparison of the two translation versions revealed no major but some slight differences. For example, the translation of 'My stomach was aching' was 'Ndairwadziwa nemudumbu' in version 1 (stomach was aching) and 'Pane pandaimborwadziwa nemudumbu' (sometimes had stomach ache) in version 2. Standard cut-offs of SSQ score >=8 indicate risk of being affected by CMD and a score >=11 indicate at risk of being severely affected by CMD [17].

The Strengths and Difficulties Questionnaire (SDQ) [19] is a widely-used freely- available 25 item scale which measures emotional and behavioural problems in the past six months. A scoping review of the use of SDQ in Africa found 54 studies in 12 countries, however, concluded that very little was known about the psychometric properties of SDQ in African settings. [20] A validated Shona version of the SDQ does not exist. Shona speaking study team members created a Shona version which was then back translated and checked by a psychiatrist (DC). The SDQ has five subscales: Emotional symptoms, conduct problems, hyperactivity, peer problems and prosocial behaviour.[21] Each scale has 5 items each with the response categories ('not true', 'somewhat true', and 'certainly true') associated with a score between 0 and 2. The mean SDQ-25 score is used as a measure of psychosocial well-being, with the maximum total SDQ score (sum of all scales except the prosocial scale) of 40. In low-risk or general population samples, responses to SDQ items can be used to create a three-subscale division i.e. internalising problems (sum of emotional and peer scales, 10 items), externalising problems (sum of conduct and hyperactivity scales, 10 items) and the prosocial scale (5 items).[22] Additionally, respondents can be described according to their SDQ 'caseness' profile: (i)Neither subscale elevated, (ii)elevated internalising sub-scale, (iii)elevated externalising sub-scale, and (iv) both sub-scales elevated. To facilitate the use of SDQ as a screening tool, UK norms for mean total SDQ and sub-scale scores have been created to allow identification of the 80th-90th centile (borderline) and >90th centile

For peer review only

(abnormal) scores.[19] We used the self-report version of the SDQ questionnaire for adolescents 11-17 years (https://www.sdqinfo.org/py/sdqinfo/b0.py).

Data management and analysis

Data were collected and recorded using Open Data Kit (ODK) survey software with built-in logic checks and skip patterns on Android tablets. Data was analysed using STATA v17.0 (StatCorp, Tx, USA) using survey commands to account for the one-stage cluster sampling design.

The prevalence of common mental health disorders was described according to the standard adult cut-offs of SSQ score >=8 and score >=11 defining those at risk of being affected and severely affected by CMDs, respectively. We also described the prevalence of those at risk of being affected by CMD using a more conservative cut-off of SSQ>=9.[23] The Cronbach's alpha intra-class correlation coefficient was calculated to assess internal consistency of the SSQ scale with high internal consistency traditionally defined as alpha>0.7. Adjusted Wald tests were used to compare proportions between sub-populations. Age-adjusted Wald tests were used to explore potential differences in the SSQ findings according to the translation version of the questionnaire used.

Mean SDQ scores (total, domain and sub-scale) and their internal consistencies were described. UK norms, and where necessary Zimbabwe-specific norms, were used to define normal, borderline and abnormal scores for each of the domains. We calculated the mean, standard deviation, and Chronbach's alpha for the SDQ scores according to sex. Age-adjusted differences in means according to sex were examined taking into account the clustered sampling design. The proportion scores in the 'normal', 'borderline' and 'abnormal' ranges in this Zimbabwean sample were calculated based on cut-offs derived by the SDQ authors using UK data.[24] As the use of UK norms have been shown to be inappropriate in other settings,[25] Zimbabwean cut-offs were generated to classify the 80th-90th centile and >90th centile bands. Participants were described according to their SDQ 'caseness' profile.

Participants with missing data for one or more of the SSQ or SDQ questions were excluded from the analysis of that scale. Multiple logistic regression was used to explore the factors associated with risk of being affected by CMDs (SSQ>=8), with adjustment for the one-stage cluster sampling design. Potential explanatory variables were age, sex, marital status, community of residence, highest level of school attended, current occupational status, religion, travel for at least 1 month in past 12 months, length of time living in the community and orphanhood status, with age being considered an *a priori* potential confounder. Wald tests adjusted for the clustered sampling design were used at each step of the analysis. Community D, where only ten participants were interviewed due to logistical reasons, was excluded from regression analysis.

Ethical considerations

Ethical approval was obtained from Institutional Review Board of the Biomedical Research and Training Institute (BRTI, AP149/2018), the Medical Research Council of Zimbabwe (MCRZ, MRCZ/A/2362) and the London School of Hygiene and Tropical Medicine Research Ethics Committee (LSHTM REC, No.15919). Written informed consent was obtained from parents or guardians of study participants aged below 16 years, along with participant assent. Participants aged 16 years and older consented independently.

YP identified as as needing further assessment based on their SSQ responses were referred immediately to health facilities for further assessment. Participants who scored 11 or higher on the SSQ or who responded 'yes' to either of the following questions were considered in need of further assessment: item 5 "In the past week, I sometimes saw or heard things which others could not see or hear"; item 11 "In the past week, at times I felt like committing suicide". In such instances, the research assistant was required to accompany the respondent to the nearest health facility so that they could seek professional care e.g. for mental health issues. At the time of study commencement, participants

For peer review only - http://bmjopen.bmj.com/site/about/guidelines.xhtml

were referred to the “Friendship Bench” service at local government health facilities. The Friendship Bench lay health workers deliver basic cognitive behavioural therapy with an emphasis on problem solving therapy, activity scheduling, and peer led group support. However, local mental health services were reduced as staff were dealing with a cholera outbreak and young people were reluctant to attend as they were not comfortable discussing their issues with the elderly counsellors. An alternative referral pathway was established, whereby, young people were referred to a psychiatrist’s private clinic. Travel and initial consultation expenses were covered by the project and uptake of referrals was monitored through phone calls with the young people and through communication with the doctor.

Patient and public involvement

Patients and the public were not directly involved in the design, conduct or analysis of this study. Formative work conducted in 2018 in the study communities as part of the CHIEDZA trial involved workshops and interviews with young people with the aim of understanding their health priorities and health service preferences.

Results:

A total of 1212 households were sampled from 140 GPS point clusters in five communities (A 25, B 48, C 21, D 42, E 4) Figure 1. YP in the target age range (n=719) were identified in 491 households (41.1% of successfully interviewed households). Stratified sampling led to the recruitment of approximately 60% of participants from Harare, and 40% of the participants from Mashonaland East.

Figure 1: Survey recruitment (HH Household, YP young people)

INSERT Figure 1 HERE

Demographic profile of study population

A total of 634/719 (88.2%) eligible YP, mean age 18.0 years (SD 3.3) and 62.6% (397/634) female, participated. The majority (83.9%, 532/634) had never been married, 86.8% (550/634) had attended secondary school or higher, and only 14.7% (51/346) of out of school participants reported that they were working. Over a quarter (28.7%, 182/634) reported that they had travelled for at least one month in the past 12 months and 17.7% (112/634) had lived less than one year in their community. Approximately one third of respondents reported that one or both of their parents were dead or that a parent’s location was unknown (**Table 1**).

Common mental health disorders

The median SSQ score was 7 (IQR 4, 9) overall, 6 (IQR 4,9) in males, and 7 (IQR 4,9) in females. Cronbach’s alpha was 0.77 suggesting that the instruments are measuring the same construct (mood). In total, 37.38% (237/634) were at risk of CMD (SSQ >=8) and 11.51% (73/634) were severely at risk of CMD (SSQ >=11). Using a more conservative cut-off of SSQ>=9, 28.55% (181/634) were at risk of CMD. A fifth of those interviewed (22.08%) were identified as needing further assessment i.e. had a SSQ >=11 or reported having seen or heard things which others could not see or hear (9.78% (62/634)) or that they felt like committing suicide in the past week 11.20% (71/634). The prevalence of CMD was similar according to the translation version of the questionnaire used **(Table S1**). The prevalence of CMD was similar among males and females. However, males were more likely than females to report having heard or seen things which others could not see or hear (14.35% vs. 7.05%, p=0.01)) and

For peer review only - http://bmjopen.bmj.com/site/about/guidelines.xhtml

females were more likely to report suicidal ideation than males (13.60% vs. 7.17%, p=0.01). **(Table 2)**. Individual SSQ question responses and outcomes according to age group are presented in **Table S2**.

A total of 140 young people out of 634 (22.08%) were identified as needing immediate referral for assessment by a psychiatrist, however, we were only able to confirm that 23 of these young people (16%) were seen by the psychiatrist. The study team attempted to revisit the young people to encourage them to attend the referral but many refused, were too busy or had relocated. It is possible that some participants may have accessed the Friendship Bench at local health facilities.

For peer review only

Table 1: Demographic characteristics of the study population (N=634)

	**Sex of respondent**					
	Male (n=237, 37.4%)		Female (n=397, 62.6%)		Total (N=634)	
	No.	%	No.	%	No.	%
**Age group (years)**						
13-15y	65	27.4	96	24.2	161	25.4
16-17y	47	19.8	74	18.6	121	19.1
18-19y	55	23.2	89	22.4	144	22.7
20-24y	70	29.5	138	34.8	208	32.8
**Mean age (years)**	17.8 (17.4, 18.2)		18.2 (17.8, 18.5)		18.0 (17.8, 18.3)	
**Marital status**						
Married	5	2.1	64	16.1	69	10.9
Cohabiting	1	0.4	15	3.8	16	2.5
Never married	225	94.9	307	77.3	532	83.9
Divorced/separated	6	2.5	11	2.8	17	2.7
**Highest level of school attended**						
Primary	32	13.5	50	12.6	82	12.9
Secondary	195	82.3	330	83.1	525	82.8
Higher (Tertiary)	9	3.8	16	4	25	3.9
Never been to school	1	0.4	1	0.3	2	0.3
**Current occupational status**						
In school/university	121	51.1	167	42.1	288	45.4
Out of school (working)	22	9.3	29	7.3	51	8
Out of school (not working)	94	39.7	201	50.6	295	46.5
**Religion**[1]						
Roman Catholic	27	11.5	38	9.6	65	10.3
Protestant	55	23.5	102	25.7	157	24.9
Pentecostal	96	41.0	169	42.6	265	42.0
Apostolic	19	8.1	67	16.9	86	13.6
Other Christian/Muslim/Other	5	2.1	3	0.8	8	1.3
No religion	32	13.7	18	4.5	50	7.9
**Travelled for at least 1 month in past 12 months**						
No	170	71.7	282	71	452	71.3
Yes	67	28.3	115	29	182	28.7

For peer review only - http://bmjopen.bmj.com/site/about/guidelines.xhtml

**How long lived in community?** [2]						
<1 year	29	12.3	83	20.9	112	17.7
1-4 years	44	18.7	98	24.7	142	22.5
5+ years	162	68.9	216	54.4	378	59.8
**Orphan status**						
Double orphan	23	9.7	40	10.1	63	9.9
Mother dead, father alive	21	8.9	31	7.8	52	8.2
Mother alive, father dead or unknown	40	16.9	70	17.6	110	17.4
Both parents alive	153	64.6	256	64.5	409	64.5

[1]n=3 no response [2] n=2 don't know

For peer review only

Table 2: Shona symptom questionnaire (SSQ): item response rates by sex and overall response (n=634)

	**Total** [a]	**Male** [a]	**Female** [a]	**p-value for age-adjusted difference between sexes** [b]
n	634	237	397	
Median SSQ (IQR)	7 (4, 9)	6 (4,9)	7 (4,9)	
Mean SSQ (95% CI)	6.44 (6.13, 6.74)	6.37 (5.95, 6.79)	6.48 (6.11, 6.84)	p=0.811
At risk for CMD (SSQ>=8)	37.38 (32.99, 41.99)	34.18 (27.76, 41.23)	39.29 (34.11, 44.73)	p=0.278
At risk for CMD (SSQ>=9)	28.55 (24.88, 32.53)	26.58 (21.49, 32.39)	29.72 (25.22, 34.65)	p=0.443
Severely at risk of CMD (SSQ>=11)	11.51 (9.08, 14.51)	10.97 (7.86,15.11)	11.84 (8.79, 15.76)	p=0.803
I sometimes saw or heard things which others could not see or hear	9.78 (7.47, 12.71)	14.35 (10.03, 20.11)	7.05 (4.72, 10.41)	p=0.011
At times I felt like committing suicide	11.20 (9.01, 13.84)	7.17 (4.57, 11.09)	13.60 (10.69, 17.16)	p=0.012
In need of further assessment	22.08 (18.55, 26.07)	21.10 (16.20, 27.00)	22.67 (18.36, 27.66)	p=0.693

a. mean and proportions taking into account clustered study design but not adjusted for age
b. Adjusted Wald test accounting for clustered study design and adjusting for age in years

Prevalence of emotional and behavioural disorders

For peer review only - http://bmjopen.bmj.com/site/about/guidelines.xhtml

The mean total difficulties SDQ score was 10.7 (SD 6.3, alpha 0.75), externalising symptoms score was 4.31 (SD 3.48, alpha 0.68) and internalising symptom score was 6.43 (SD 3.94, alpha 0.63). The mean scores for emotional symptoms, conduct problems, hyperactivity/inattention, and peer problems were 3.66, 2.22, 2.09 and 2.77 respectively. The mean prosocial behaviour score was 8.13 (**Table S3**).

The Cronbach's alpha, an indication of internal consistency of the measure, was 0.75 for the total difficulties score and ranged from 0.38 to 0.68 for sub-scales. There was no evidence of a sex difference in total difficulties score and weak evidence of males having higher externalising scores than females (4.63 vs 4.11, p=0.05) and females having higher internalising scores than males (6.64 vs 6.08, p=0.08) (**Table S4**).

Using UK norms to define normal, borderline and abnormal scores for each of the domains, a high proportion (15.8%) of Zimbabwean young people had abnormal scores for emotional symptoms, and a low proportion had abnormal scores for hyperactivity/inattention scores (2.8%) and prosocial scores (7.1%). Using the Zimbabwean study data, we created country-specific cut-offs where the scores within each band differed for the emotional symptoms, hyperactivity/attention, and prosocial domains only. Using the Zimbabwean norms, a total of 13.9% had an elevated internalising sub-scale, 11.5% an elevated externalising sub-scale and 6.9% with both scales elevated (**Table 3**).

For peer review only

For peer review only - http://bmjopen.bmj.com/site/about/guidelines.xhtml

Table 3: Strength and Difficulties Questionnaire (SDQ): banding of self-report raw scores in Zimbabwe and UK

		UK norms				Zimbabwe norms			
			All (n=634)	Male (n=237)	Female (n=397)		All (n=634)	Male (n=237)	Female (n=397)
		**Band**	**%**	**%**	**%**	**Band**	**%**	**%**	**%**
Total Difficulties	Normal	0-15	78.4	78.9	78.1	0-15	78.4	78.9	78.1
	Borderline	16-19	11	11.4	10.8	16-19	11.0	11.4	10.8
	Abnormal	20-40	10.6	9.7	11.1	20-40	10.6	9.7	11.1
Emotional symptoms	Normal	0-5	74.1	77.6	72.0	0-5	74.1	77.6	72.0
	Borderline	6	10.1	10.1	10.1	6 to 7	13.7	13.9	13.6
	Abnormal	7 to 10	15.8	12.2	17.9	8 to 10	12.2	8.4	14.4
Conduct problems	Normal	0-3	74.9	75.1	74.8	0-3	74.9	75.1	74.8
	Borderline	4	12.8	11.0	13.9	4	12.8	11.0	13.9
	Abnormal	5 to 10	12.3	13.9	11.3	5 to 10	12.3	13.9	11.3
Hyperactivity/inattention	Normal	0-5	92.3	89.9	93.7	0-3	77.3	72.6	80.1
	Borderline	6	4.9	6.8	3.8	4	11.0	13.5	9.6
	Abnormal	7 to 10	2.8	3.4	2.5	5 to 10	11.7	13.9	10.3
Peer problems	Normal	0-3	64.7	66.2	63.7	0-3	64.7	66.2	63.7
	Borderline	4 to 5	22.7	23.2	22.4	4 to 5	22.7	23.2	22.4
	Abnormal	6 to 10	12.6	10.6	13.9	6 to 10	12.6	10.6	13.9
Prosocial behaviour	Normal	6 to 10	89.9	90.7	89.4	7 to 10	79.2	77.6	80.1

For peer review only - http://bmjopen.bmj.com/site/about/guidelines.xhtml

	Borderline	5	3.0	3.4	2.8	6	10.7	13.1	9.3
	Abnormal	0-4	7.1	5.9	7.8	0 to 5	10.1	9.3	10.6
SDQ caseness profile	Neither subscale elevated						67.7	67.9	67.5
	Elevated internalising sub-scale						13.9	10.1	16.1
	Elevated externalising sub-scale						11.5	15.6	9.1
	Elevated internalising and externalising sub-scale						6.9	6.3	7.3

For peer review only

For peer review only - http://bmjopen.bmj.com/site/about/guidelines.xhtml

Correlates of being at risk of common mental health disorders

The odds of CMD increased by 9% with each year increase in age (OR 1.09, p<0.001). Those who were out of school and not working had higher odds of CMD compared to those in school or those working (adjusted OR 1.67 (1.07, 2.62), p=0.04). Following adjustment for age, there was no evidence of an association between risk of CMD and participants' sex, marital status, education level, religion, travel in the past 12 months, length lived in the community or orphanhood status **(Table 4)**.

Table 4: Associations between demographic characteristics and >=8 score on SSQ

	N	n	%	Crude OR	Age Adj OR
**Sex**					
Male	237	81	34.18 (27.76, 41.23)	REF	REF
Female	387	150	38.76 (33.61, 44.18)	1.22 (0.87, 1.72)	1.19 (0.84, 1.67)
			p=0.254[a]	p=0.254	
**Age group**					
13-15y	159	42	26.42 (19.46, 34.79)	REF	
16-17y	119	47	39.50 (31.17, 48.48)	1.86 (1.14, 3.05)	
18-19y	144	58	40.28 (31.98, 49.18)	1.91 (1.16, 3.14)	
20-24y	202	84	41.58 (35.67, 47.75)	2.12 (1.37, 3.27)	
			p=0.0117		
**Age in years**				1.09 (1.04, 1.15)	
				p<0.001	
**Marital status**					
Married/Cohabiting	79	40	50.63 (40.54, 60.68)	REF	REF
Never married	528	182	34.47 (29.76, 39.51)	0.51 (0.32, 0.82)	0.68 (0.40, 1.13)
Widowed/Divorced/separated	17	9	52.94 (31.08, 73.73)	1.10 (0.37, 3.23)	1.06 (0.36, 3.17)
			p=0.0071	p=0.0035	p=0.22
**Highest level of school attended**					
None/primary	82	21	25.61 (16.10, 38.19)	REF	REF
Secondary	517	203	39.26 (34.37, 44.38)	1.88 (1.01, 3.51)	1.32 (0.67, 2.60)
Higher	25	7	28.00 (13.96, 48.25)	1.13 (0.43, 2.94)	0.57 (0.21, 1.56)
			p=0.0696	p=0.1228	p=0.1625
**Occupation**					
In school	287	83	28.92 (23.13, 35.50)	REF	REF
Out of school working	51	18	35.29 (22.70, 50.33)	1.34 (0.71, 2.52)	0.99 (0.49, 2.00)
Out of school not working	286	130	45.45 (39.45, 51.60)	2.05 (1.40, 2.99)	1.67 (1.07, 2.62)
			p=0.0006	p=0.0013	p=0.0410

For peer review only - http://bmjopen.bmj.com/site/about/guidelines.xhtml

For peer review only

**Religion**					
Roman Catholic	64	22	34.38 (23.59, 47.05)	REF	REF
Other Christian/ Muslim/No religion	557	208	37.34 (32.72, 42.21)	1.14 (0.65, 1.98)	1.19 (0.67, 2.11)
			p=0.6452	p=0.6454	p=0.5538
**Community of residence**					
A	178	71	39.89 (31.41,49.02)	REF	REF
B	140	48	34.29 (26.11,43.51)	0.78 (0.46, 1.35)	0.78 (0.45, 1.34)
C	147	64	43.54 (34.60,52.91)	1.16 (0.69, 1.97)	1.16 (0.68, 1.97)
E	159	48	30.19 (22.73,38.87)	0.65 (0.38, 1.11)	0.66 (0.38, 1.13)
				p=0.1625	p=0.1711
**Travelled for at last 1 month in last 12 months**					
No	444	161	36.26 (31.79, 40.99)	REF	REF
Yes	180	70	38.89 (30.47, 48.03)	1.12 (0.76, 1.65)	1.11 (0.75, 1.65)
			p=0.5705	p=0.5706	p=0.5980
**How long lived in the community**					
<1 year	112	42	37.50 (29.04, 46.80)	REF	REF
1-4 years	138	56	40.58 (32.45, 49.26)	1.14 (0.72, 1.81)	1.14 (0.70, 1.84)
5+ years	372	133	35.75 (30.37, 41.52)	0.93 (0.60, 1.44)	0.99 (0.64, 1.54)
			p=0.5865	p=0.6234	p=0.7846
**Orphan status**					
Double orphan	61	28	45.90 (33.22, 59.14)	REF	REF
Mother dead, father alive	52	25	48.08 (35.92, 60.46)	1.09 (0.51, 2.33)	1.08 (0.51, 2.31)
Mother alive, father dead	108	36	33.33 (24.94, 42.93)	0.59 (0.30, 1.15)	0.61 (0.31, 1.20)
Both parents alive	403	142	35.24 (29.83, 41.04)	0.64 (0.37, 1.11)	0.76 (0.44, 1.31)
			p=0.1118	p=0.0861	p=0.2217

[a] *p-value from adjusted wald test*

Note Community D (n=10) dropped from this analysis so total N is 624

Adjusted for age in years

Discussion:

Principal findings

We observed a high prevalence of self-reported symptoms of CMD in this population sample of 13-24 year olds in urban and peri-urban Zimbabwe. Over one in five young people required immediate referral for psychiatrist assessment but there was very low uptake of referral to mental health services. The odds of being affected by CMD increased with age and was higher among those out of

For peer review only

For peer review only - http://bmjopen.bmj.com/site/about/guidelines.xhtml

school and not working. Self-reported emotional and behavioural disorders were also common. Compared to UK norms for the Strength and Difficulties questionnaire, a lower proportion had abnormal scores for hyperactivity/inattention and prosocial sub-scales and a higher proportion had abnormal emotional symptoms scores which necessitated the creation of Zimbabwe-specific norms.

Common mental and emotional and behavioural disorders

We found 37% of young participants to be at risk of CMD (SSQ>=8), and 12% to be severely at risk of CMD (SSQ>=11). High prevalence of symptoms of CMD was also observed in a 2006 population-based survey among rural Zimbabwean youth (15-23 years)[3], and among HIV+ adolescents in Harare in 2009 [5] and in two rural districts in 2016/17.[10] Considerably lower prevalence of symptoms of CMDs was observed in a 2009-11 general population survey of 15-24 year olds.[4] [26] While all these studies used the SSQ, it is difficult to make comparisons given the different populations, socio-economic environments, and modes of data collection used i.e. self-completed vs interviewer administered. Previous research has shown that symptom reporting varied according to method with higher reporting in studies using self-administered methods.[27] The observed increase in risk of CMD with age is consistent with the literature.[28-31] We did not observe a higher prevalence of CMD among females compared to males which is in contrast to other adolescent studies. [32, 33]

Similar high prevalence of CMD has been observed in systematic reviews of studies among adolescents in sub-Saharan Africa with estimated prevalence of 27% for depression and 30% for anxiety disorders,[28] and 30-50% for emotional or behavioural difficulties or significant psychological distress in adolescents with HIV.[29]

The high prevalence of symptoms of CMD among young people in Zimbabwe may in part be due to the challenging economic situation with limited job opportunities, and the HIV and associated orphanhood epidemic.[34] Higher prevalence of CMD symptoms among those out of school and not working in this study has also been observed in HIV+ adolescent populations.[29] Interviews with adolescents who participated in the recent youth version of the Friendship Bench suggest that hopelessness and a feeling of lack of control over one's situation have an important impact on mental wellbeing.[35]

Measurement of common mental health and emotional and behavioural disorders

This study demonstrated that self-reported SSQ and SDQ are feasible to measure in 13-24 year olds in urban and peri-urban Zimbabwe. While the SSQ has been validated in several populations,[17, 23] there is no consensus on the most appropriate cut-offs to be used for young people. [3, 10, 26] Some authors have found that a cut-off of ≥5 provides the best performance and increases sensitivity of the screening tool, [26] however, their conclusions were based on interviewer-administered questionnaires where some of the SSQ questions had non-standard wording. A more recent validation of SSQ against MINI-KID in 12-17 year olds attending primary care clinics in urban Zimbabwe found the optimal cutpoint of SSQ >=8 (Unpublished data).

In this first documented use of a Shona language version of the Strength and difficulties questionnaire we observed good internal consistency for total difficulties but lower consistency within some of the sub-scales. In line with studies in other sub-Saharan African settings, the translation of a few of the terms within the SDQ were challenging,[20] for example, in the hyperactivity sub-scale there is a question about 'fidgeting' for which there is no equivalent term in Shona. The patterns of emotional and behavioural disorders observed in this study (lower abnormal prosocial and hyperactivity sub-scale scores, higher emotional and peer sub-scale scores) are similar to patterns observed in studies of South African adolescents [25] and children[36], and Namibian adolescents.[37]

For peer review only - http://bmjopen.bmj.com/site/about/guidelines.xhtml

Multiple screening tools exist for common mental health disorders and while the SSQ is widely used in Zimbabwe, and the SDQ used widely globally, there may be other tools that are more appropriate for the Zimbabwean adolescent population. A first step will therefore be to decide on which tool or tools are most promising and then plan the necessary careful adaptation and validation work. UNICEF with WHO and other partners are currently developing tools for the Measurement of Mental Health Among Adolescents at the Population Level (MMAP) with a focus on PHQ-9 and GAD-7.[38] If the SDQ is to be used again in Zimbabwean adolescents then additional work is needed to develop a Shona version and then to develop country-specific SDQ cut-offs for 'caseness'. [25]

Services for young people

The high burden of CMD among young people and reluctance to attend adult mental health services demonstrate an urgent need for youth-friendly mental health services. The original referral pathway was to refer the young people to the Friendship Bench Service at nearby local government health facilities. However, health facility staff shortages due to an ongoing cholera outbreak coupled with YPs reluctance to access adult Friendship Bench services required the creation of an alternative referral pathway. While some young people successfully attended referral appointments at the hospital in Harare, there were many young people who did not attend care. This study has highlighted the need to involve young people when developing research study protocols and to establish the feasibility and acceptability of proposed referral pathways. In addition to increasing the availability of high-quality youth-friendly mental health services, increased education and skill-building for young people and their families may help to support healthy mental health and well-being.[31]

Strengths and weaknesses of the study

A strength of this study is a representative general population sample of young people in five urban and peri-urban communities in Zimbabwe, in the context of limited population-level estimates of mental health disorders among young people. We demonstrated the feasibility of using ACASI to collect self-reported measures of mental health and emotional and behavioural disorders in this young population. Only the self-report version of the SDQ was used, however, for optimal use it is recommended that teachers and parents also complete the SDQ allowing triangulation of data. [39] The tools used were not validated for use in the full age group, the translation of the Strength and Difficulties Questionnaire into Shona did not follow recommended procedures, and two different Shona versions of SSQ were used. We do not think that the use of different SSQ versions had a big impact on the results e.g. similar mean and median SSQ scores (Table S1). When version 1 was used, there was a higher prevalence of those at risk for CMD compared to when version 2 was used, but the differences may have been due to population characteristics and not the tools themselves. The self-reported measures used may have been subject to recall bias and social desirability bias.

Conclusion:

We observed a high prevalence of probable CMDs among general population urban and peri-urban young people, however there was a low uptake of referrals to existing mental health services. The use of self-reported SSQ and SDQ were feasible in this population but additional research is needed to determine the validity and most appropriate cut-offs for these measurement tools in young Zimbabweans. Important outstanding research questions for young people's mental health in this resource-constrained setting include how to identify those most at risk, and how to ensure that they are linked to appropriate youth-focused mental health services.

For peer review only - http://bmjopen.bmj.com/site/about/guidelines.xhtml

Author contributions

Obtained funding: AMD, RAF

Wrote the study protocol: AMD, TB, ED, GM, CG, DC, RAF

Assisted with fieldwork and data collection which was overseen by: AMD, ED, TB

Did central data monitoring: TB

Conducted statistical analysis: AMD

Advised on statistical analysis: VS

Drafted the initial manuscript: AMD

Provided substantial edits to the manuscript: TB, ED, GM, CG, VS, DC, RAF

All authors contributed to interpretation of the results and have read and approved the final manuscript

Funding

Financial support for this study was provided through joint funding under the UK Medical Research Council (MRC)/ UK Department for International Development (DFID) Concordat agreement which is supported by the European Union under the EDCTP2 programme (reference MR/K012126/1) with AMD receiving support (G0700837). AMD is supported by a UKRI Future Leader Fellowship [grant number MR/T043156/1]. RAF is funded by the Wellcome Trust through a Senior Fellowship in Clinical Science (206316Z/17/Z).

Disclaimer

The funders had no role in study design, data collection and analysis, decision to publish or preparation of the manuscript.

Competing interests

None declared

Data availability

Data are available upon reasonable request. Data for this study are available upon reasonable request to the principal authors.

Acknowledgements

We thank the study participants and their communities for participating in and facilitating this research. We are grateful to the field team who conducted the interviews, and the other Biomedical Research and Training institute, Harare, who supported the study.

Abbreviations

CMDs Common mental health disorders

SDQ Strength and Difficulties Questionnaire

ACASI Audio-computer assisted self-interview

For peer review only - http://bmjopen.bmj.com/site/about/guidelines.xhtml

SSQ Shona Symptoms Questionnaire

ODK Open Data Kit

Ethics Approval Statement

Ethical approval was obtained from Institutional Review Board of the Biomedical Research and Training Institute (BRTI, AP149/2018), the Medical Research Council of Zimbabwe (MCRZ, MRCZ/A/2362) and the London School of Hygiene and Tropical Medicine Research Ethics Committee (LSHTM REC, No.15919). Written informed consent was obtained from parents or guardians of study participants aged below 16 years, along with participant assent. Participants aged 16 years and older consented independently.

For peer review only

References:

1. Global Burden of Disease, *Global, regional, and national burden of 12 mental disorders in 204 countries and territories, 1990-2019: a systematic analysis for the Global Burden of Disease Study 2019.* Lancet Psychiatry, 2022.
2. Guthold, R., et al., *The Top Global Causes of Adolescent Mortality and Morbidity by Age and Sex, 2019.* J Adolesc Health, 2021. **69**(4): p. 540.
3. Langhaug, L.F., et al., *High prevalence of affective disorders among adolescents living in Rural Zimbabwe.* J Community Health, 2010. **35**(4): p. 355-64.
4. Tlhajoane, M., et al., *Prevalence and Associations of Psychological Distress, HIV Infection and HIV Care Service Utilization in East Zimbabwe.* AIDS Behav, 2018. **22**(5): p. 1485-1495.
5. Mavhu, W., et al., *Enhancing psychosocial support for HIV positive adolescents in Harare, Zimbabwe.* PLoS One, 2013. **8**(7): p. e70254.
6. Willis, N., et al., *Understanding the experience and manifestation of depression in adolescents living with HIV in Harare, Zimbabwe.* PLoS One, 2018. **13**(1): p. e0190423.
7. Mangezi, W. and D. Chibanda, *Mental health in Zimbabwe.* Int Psychiatry, 2010. **7**(4): p. 93-94.
8. Guthold, R., et al., *Priority Areas for Adolescent Health Measurement.* J Adolesc Health, 2021. **68**(5): p. 888-898.
9. Erskine, H.E., et al., *The global coverage of prevalence data for mental disorders in children and adolescents.* Epidemiol Psychiatr Sci, 2017. **26**(4): p. 395-402.
10. Mavhu, W., et al., *Effect of a differentiated service delivery model on virological failure in adolescents with HIV in Zimbabwe (Zvandiri): a cluster-randomised controlled trial.* Lancet Glob Health, 2020. **8**(2): p. e264-e275.
11. Doyle, A.M., et al., *Mobile Phone Access and Implications for Digital Health Interventions Among Adolescents and Young Adults in Zimbabwe: Cross-Sectional Survey.* JMIR Mhealth Uhealth, 2021. **9**(1): p. e21244.
12. Dziva Chikwari, C., et al., *The impact of community-based integrated HIV and sexual and reproductive health services for youth on population-level HIV viral load and sexually transmitted infections in Zimbabwe: protocol for the CHIEDZA cluster-randomised trial [version 1; peer review: awaiting peer review].* Wellcome Open Research, 2022. **7**(54).
13. GSMA, *A toolkit for researching women's internet access and use*. 2018, GSM Association. p. 48.
14. MACRO, *Study of Mobile Phone Usage Among the Teenagers and Youth In Mumbai*. 2004, MACRO.

For peer review only - http://bmjopen.bmj.com/site/about/guidelines.xhtml

15. Kreutzer, T., *Online and Digital Media Usage on Cell Phones among low-Income Urban Youth in Cape Town* in *Centre for Film and Media Studies*. 2009, University of Cape Town: Cape Town. p. 123.
16. UNICEF, *mHealth and Young People in South Africa*. 2017. p. 48.
17. Patel, V., et al., *The Shona Symptom Questionnaire: the development of an indigenous measure of common mental disorders in Harare.* Acta Psychiatr Scand, 1997. **95**(6): p. 469-75.
18. Verhey, R., J.D. Turner, and D. Chibanda, *The Friendship Bench Training Manual*.
19. Goodman, R., *The Strengths and Difficulties Questionnaire: a research note.* J Child Psychol Psychiatry, 1997. **38**(5): p. 581-6.
20. Hoosen, N., et al., *The Strengths and Difficulties Questionnaire (SDQ) in Africa: a scoping review of its application and validation.* Child Adolesc Psychiatry Ment Health, 2018. **12**: p. 6.
21. Goodman, R., *Psychometric properties of the strengths and difficulties questionnaire.* J Am Acad Child Adolesc Psychiatry, 2001. **40**(11): p. 1337-45.
22. Goodman, A., D.L. Lamping, and G.B. Ploubidis, *When to use broader internalising and externalising subscales instead of the hypothesised five subscales on the Strengths and Difficulties Questionnaire (SDQ): data from British parents, teachers and children.* J Abnorm Child Psychol, 2010. **38**(8): p. 1179-91.
23. Chibanda, D., et al., *Validation of screening tools for depression and anxiety disorders in a primary care population with high HIV prevalence in Zimbabwe.* J Affect Disord, 2016. **198**: p. 50-5.
24. *Scoring the Strengths & Difficulties Questionnaire for age 4-17*. 2014 [cited 2022 25 Jan 2022]; Available from: https://www.ehcap.co.uk/content/sites/ehcap/uploads/NewsDocuments/236/SDQEnglishUK4-17scoring-1.PDF.
25. de Vries, P.J., et al., *Measuring adolescent mental health around the globe: psychometric properties of the self-report Strengths and Difficulties Questionnaire in South Africa, and comparison with UK, Australian and Chinese data.* Epidemiol Psychiatr Sci, 2018. **27**(4): p. 369-380.
26. Haney, E., et al., *One size does not fit all: psychometric properties of the Shona Symptom Questionnaire (SSQ) among adolescents and young adults in Zimbabwe.* J Affect Disord, 2014. **167**: p. 358-67.
27. Langhaug, L.F., et al., *Difference in prevalence of common mental disorder as measured using four questionnaire delivery methods among young people in rural Zimbabwe.* J Affect Disord, 2009. **118**(1-3): p. 220-3.
28. Jörns-Presentati, A., et al., *The prevalence of mental health problems in sub-Saharan adolescents: A systematic review.* PLoS One, 2021. **16**(5): p. e0251689.
29. Dessauvagie, A.S., et al., *The prevalence of mental health problems in sub-Saharan adolescents living with HIV: a systematic review.* Glob Ment Health (Camb), 2020. **7**: p. e29.
30. Too, E.K., et al., *Prevalence and factors associated with common mental disorders in young people living with HIV in sub-Saharan Africa: a systematic review.* J Int AIDS Soc, 2021. **24 Suppl 2**(Suppl 2): p. e25705.
31. WHO, *Global accelerated action for the health of adolescents (AA-HA!): guidance to support country implementation*. 2017. p. 148.
32. Anjum, A., et al., *Investigating the prevalence of and factors associated with depressive symptoms among urban and semi-urban school adolescents in Bangladesh: a pilot study.* Int Health, 2019.
33. Girma, S., et al., *Depression and its determinants among adolescents in Jimma town, Southwest Ethiopia.* PLoS One, 2021. **16**(5): p. e0250927.
34. Nyamukapa, C.A., et al., *Causes and consequences of psychological distress among orphans in eastern Zimbabwe.* AIDS Care, 2010. **22**(8): p. 988-996.

For peer review only - http://bmjopen.bmj.com/site/about/guidelines.xhtml

35. Broström, S., et al., *"Seeing a Brighter Future" - Experiences of Adolescents with Common Mental Disorders Receiving the Problem-Solving Therapy "Youth Friendship Bench" in Zimbabwe.* Issues Ment Health Nurs, 2021. **42**(11): p. 1019-1029.
36. Mellins, C.A., et al., *Screening for Mental Health Among Young South African Children: The Use of the Strengths and Difficulties Questionnaire (SDQ).* Glob Soc Welf, 2018. **5**(1): p. 29-38.
37. Gentz, S.G., et al., *Predictors of mental health problems in adolescents living with HIV in Namibia.* Child Adolesc Ment Health, 2017. **22**(4): p. 179-185.
38. Carvajal, L., J.H. Requejo, and C.E. Irwin, *The Measurement of Mental Health Problems Among Adolescents and Young Adults Throughout the World.* J Adolesc Health, 2021. **69**(3): p. 361-362.
39. Goodman, R., et al., *Using the Strengths and Difficulties Questionnaire (SDQ) to screen for child psychiatric disorders in a community sample.* Br J Psychiatry, 2000. **177**: p. 534-9.

For peer review only

For peer review only - http://bmjopen.bmj.com/site/about/guidelines.xhtml

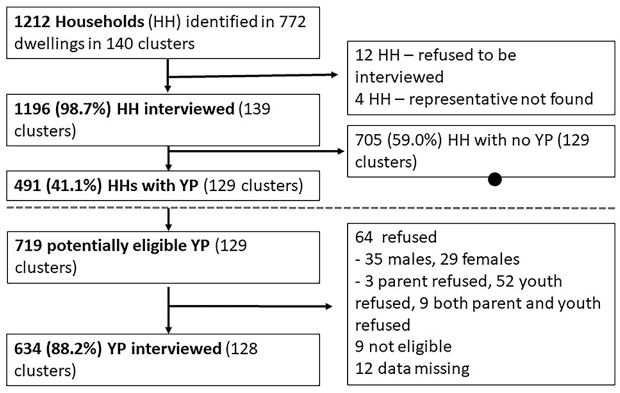
1212 Households (HH) identified in 772 dwellings in 140 clusters
12 HH – refused to be interviewed
4 HH – representative not found
1196 (98.7%) HH interviewed (139 clusters)
705 (59.0%) HH with no YP (129 clusters)
491 (41.1%) HHs with YP (129 clusters)
719 potentially eligible YP (129 clusters)
64 refused
- 35 males, 29 females
- 3 parent refused, 52 youth refused, 9 both parent and youth refused
9 not eligible
12 data missing
634 (88.2%) YP interviewed (128 clusters)

Supplementary Material

Questionnaire (YTM Q02 n6)

Shona	English
**SECTION 0 RECRUITMENT INFORMATION**	**SECTION 0 RECRUITMENT INFORMATION**
[Q01] Date of Interview	[Q01] Date of Interview
[Q02] Whats the Suburb Name?	[Q02] Whats the Suburb Name?
[Q022] Whats the Suburb Name?	[Q022] Whats the Suburb Name?
[Q03] Cluster ID?	[Q03] Cluster ID?
[Q032] Cluster ID?	[Q032] Cluster ID?
[Q04] Dwell ID?	[Q04] Dwell ID?
[Q042] Dwell ID?	[Q042] Dwell ID?
[Q05] Household ID?	[Q05] Household ID?
[Q05m] Manually enter the Household ID?	[Q05m] Manually enter the Household ID?
[Q05m] Manually enter the Household ID?	[Q05m] Manually enter the Household ID?
[Q06] Respondent No?	[Q06] Respondent No?
[Q062] Respondent No?	[Q062] Respondent No?
[Q07] Select your name	[Q07] Select your name
[Q08] Please select the language for this interview	[Q08] Please select the language for this interview
[Q101] Mukomana kana musikana here?	[Q101] Respondent Sex
[Q102] Wave nemakore mangani?	[Q102] How old were you at your last birthday?
[Q103] Panguva ino wakamira sei panyaya dzewanano?	[Q103] What is your current marital status?
[Q104] Wakambopinda chikoro here?	[Q104] Have you ever attended school?
[Q106] Kuchikoro wakasvika pai: primary, secondary, kana pamusoro?	[Q106] What is the highest level of school you attended: primary, secondary, or higher?
[Q105] Ndezvipi zvinonyatsotsanangura zvauri kuita mazuvano?	[Q105] Which of the following best describes your current occupational status?
[Q107] Uri vechitendero chipi?	[Q107] What is your religion?
[Q107] Tsanangudza chimwe chitendero?	[Q107] Specify other religion?
[Q108] Unowanzotaura mutauro upi kana usiri kumba?	[Q108] Outside your home, what language do you speak the most?
[Q108] Tsanangudza mumwe mutauro waunotaura usiri pamba?	[Q108] Specify other language?
**NGUVA YAWAGARA PAMBA PANO KANA MUNHARAUNDA INO**	**DURATION OF STAY AT RESIDENCE**
[Q109] Wave nenguva yakareba zvakadii uchigara munharaunda ino?	[Q109] How long have you lived in this community?
[Q110] Mumwedzi gumi nemiviri yapfuura, wakambofamba wakanogara kuneimwe nzvimbo kwemwedzi kana kupfuura here?	[Q110] In the last 12 months, have you travelled and stayed in a place other than this community for one month or more?
[Q111] Kusanganisa nguva yese yawakagara kunze kwenharaunda ino, ungati wakagara nguva yakadii mumwedzi negumi nemiviri yapfuura?	[Q111] In total approximately how long have you spent outside this community in the last 12 months?
**MHURI**	**FAMILY**

For peer review only

[Q112] Baba vako vakakubereka vapenyu here?	[Q112] Is your natural father alive?
[Q113] Mai vako vakakubereka vapenyu here?	[Q113] Is your natural mother alive?
Chimbonditaurira nezvinhu zve MICHINA (TECHNOLOGY) zvamunazvo mumba menyu	Please tell us about some technological devices- which of the following do you have at home
**MICHINA (TECHNOLOGY) IRI MUMBA**	**TECHNOLOGY AT HOME**
[Q201h] Normal/'desktop' computer	[Q201h] Normal/'desktop' computer
[Q201h] Laptop computer (including notebooks and netbooks)	[Q201h] Laptop computer (including notebooks and netbooks)
[Q201h] Tablet/ iPad	[Q201h] Tablet/ iPad
[Q201h] Feature (non-smart) Cell phone	[Q201h] Feature (non-smart) Cell phone
[Q201h] Basic feature (calls and sms only) Cellphone	[Q201h] Basic feature (calls and sms only) Cellphone
[Q201h] Smart cell phone	[Q201h] Smart cell phone
[Q201h6] Ipod or other MP3 player	[Q201h6] Ipod or other MP3 player
[Q201h] TV	[Q201h] TV
[Q201h] Radio	[Q201h] Radio
[Q201h] Digital camera	[Q201h] Digital camera
[Q201h] Gaming console (Playstation, Xbox, Nintendo wii etc.)	[Q201h] Gaming console (Playstation, Xbox, Nintendo wii etc.)
[Q201h] Handheld gaming device (e.g. DS, PSP, …)	[Q201h] Handheld gaming device (e.g. DS, PSP, …)
**UNOSHANDISA MICHINA(TECHNOLOGY) IPI**	**USE OF TECHNOLOGY**
[Q201u] Normal/'desktop' computer	[Q201u] Normal/'desktop' computer
[Q201u] Laptop computer (including notebooks and netbooks)	[Q201u] Laptop computer (including notebooks and netbooks)
[Q201u] Tablet/ iPad	[Q201u] Tablet/ iPad
[Q201u] Feature (non-smart) Cell phone	[Q201u] Feature (non-smart) Cell phone
[Q201u] Basic feature (calls and sms only) Cellphone	[Q201u] Basic feature (calls and sms only) Cellphone
[Q201u] Smart cell phone	[Q201u] Smart cell phone
[Q201u6] Ipod or other MP3 player	[Q201u6] Ipod or other MP3 player
[Q201u] TV	[Q201u] TV
[Q201u] Radio	[Q201u] Radio
[Q201u] Digital camera	[Q201u] Digital camera
[Q201u] Gaming console (Playstation, Xbox, Nintendo wii etc.)	[Q201u] Gaming console (Playstation, Xbox, Nintendo wii etc.)
[Q201u] Handheld gaming device (e.g. DS, PSP, …)	[Q201u] Handheld gaming device (e.g. DS, PSP, …)
[Q202] Wakamboshandisa internet here?	[Q202] Have you ever used the internet??
**KUSHANDISA INTERNET**	**USE OF INTERNET**
[Q203] Kana wakashandisa, wakapedzisira kushandisa internet rinhi?	[Q203] If yes, when is the last time that you used the internet?
Bvunza kuti anoshandisa internet kupi, nguva yakadini	Ask about when the participants uses the internet, how often do they do so
**Unoshandisa Internet nguva yakadini**	**How ofte do you use internet**
[Q204]Pacomputer kuchikoro kana kubasa	[Q204]On a computer at school/ work
[Q204]Pacomputer kumba	[Q204]On a computer at home
[Q204]Pacell/mobile phone	[Q204]On a cell/mobile phone
[Q204]Pacomputer kulibrary/ kana community internet shop	[Q204]At a computer in the library/ other community internet facility
[Q204]Ku internet café inobhadharwa	[Q204]At a commercial internet café- where you pay for the access
[Q204]Pacomputer kumba kwevamwewo	[Q204]At a computer in someone else's house
[Q205] Unopedza nguva yakadini uri painternet?	[Q205] On average how much time do you spend on the internet when you go online?
[Q206] Chii chaunofarira kunyanya painternet?	[Q206] What do you like the most about the internet?

For peer review only

[Q206] Tsanangudza chimwe chikonzero chaunofarira painternet?	[Q206] Specify other reason what you like about the internet?
[Q207] Chii chausinga farire kunyaya painternet?	[Q207] What do you dislike the most about the internet?
[Q207] Tsanangudza chimwe chikonzero chausingafarire painternet?	[Q207] Specify other reason what you dislike about the internet?
[Q208] Chii chaungade kuti chishanduke kuti kushandisa kwako internet kuve nani?	[Q208] What one change would you like to see so that use of the internet could be better for you?
[Q208] Tsanangudza chimwe chinhu chaungade kuti chishanduke?	[Q208] Specify other change you would like to see?
[Q209] Une cell phone here kana kuti unoshandisa cell phone yaunokumbidzana kana kushandisa nevamwe?	[Q209] Do you have or share mobile (cell) phone)?
**VANE MOBILE PHONE KANA VANOSHANDISA**	**OWNERSHIP AND USE OF MOBILE PHONE**
[Q209a] Kana ati hongu; Une cell phone ngani dzako pachako?	[Q209a] If Yes, how many phones you own by yourself ?
[Q209b] Kana ati hongu; Une cell phone ngani dzaunokumbidzana kushandisa nevamwe?	[Q209b] If Yes, how many phones do share with someone else?
[Q210] Une maline mangani e cell phone aunokushandisa parizvino?	[Q210] How many mobile phone numbers do you currently use?
[Q211] Mugore rapfuura, maline mangani e cell phone yawakanga unayo kusanganisira aunawo parizvino?	[Q211] How many different mobile phone numbers have you had in the past year including your current number (s)?
Ngatimbotaura nema cell phone ${Q209amob} awunawo	Lets talk about the ${Q209amob} mobile phones you own
**MHANDO DZEMAMOBILE PHONE DZAANADZO**	**MOBILE PHONE TYPES OWNED**
**Phone Yokutanga Yaanayo**	**Phone 1 Owned**
[Q212O]Foni yekutanga yaunayo inonzii?	[Q212O]What is the make of the first phone you own?
[Q212O]Model yefoni yekutanga chii?	[Q212O]What is the model of the first phone you own?
[Q212O] Foni yekutanga iyi smart phone here?	[Q212O] Is the first phone you own a smart phone?
[Q212O] Foni yekutanga iyi wakaitenga gore ripi?	[Q212O] Which year was the first phone you own purchased?
**Phone Yechipiri Yaanayo**	**Phone 2 Owned**
[Q212O]Foni yechipiri yaunayo inonzii?	[Q212O]What is the make of the second phone you own?
[Q212O]Model yefoni yechipiri chii?	[Q212O]What is the model of the second phone you own?
[Q212O] Foni yechipiri iyi smart phone here?	[Q212O] Is the second phone you own a smart phone?
[Q212O] Foni yechipiri iyi wakaitenga gore ripi?	[Q212O]Which year was the second phone you own purchased?
**Phone Yechitatu Yaanayo**	**Phone 3 Owned**
[Q212O]Foni yechitatu yaunayo inonzii?	[Q212O]What is the make of the third phone you own?
[Q212O]Model yefoni yechitatu chii?	[Q212O]What is the model of the third phone you own?
[Q212O] Foni yechitatu iyi smart phone here?	[Q212O]Is the third phone you own a smart phone?
[Q212O] Foni yechitatu iyi wakaitenga gore ripi?	[Q212O]Which year was the third phone you own purchased?
Nhatimbotaurai nezvema cell phone ${Q209bmob} aunokumbidzana nevamwe	Lets talk about the ${Q209bmob} mobile phones you share
**MOBILE PHONE TYPES SHARED**	**MOBILE PHONE TYPES SHARED**

For peer review only

**Phone Yokutanga Yaanokumbidzana kana kushandisa nevamwe**	**Phone 1 Shared**
[Q212S]Foni yekutanga yaunokumbidzana nevamwe inonzii?	[Q212S]What is the make of the first phone you share?
[Q212S]Model yefoni yekutanga chii?	[Q212S]What is the model of the first phone you share?
[Q212S] Foni yekutanga iyi smart phone here?	[Q212S] Is the first phone you share a smart phone?
[Q212S] Foni yekutanga iyi wakaitenga rinhi?	[Q212S] Which year was the first phone you share purchased?
[Q213S] Unoshandisa kana kukumbidzana cell phone yokutanga nani?	[Q213S] Who do you share ownership of your first phone with?
[Q213S] Tsanangudza vamwe vanhu vaunoshandisa phone navo	[Q213S] Specify other
[Q213S] Kakawanda zvakadii kamunowana mukana wekushandisa phone yokutanga?	[Q232S] How often do you have access to the first phone you share?
**Phone Yechipiri Yaanokumbidzana kana kushandisa nevamwe**	**Phone 2 Shared**
[Q212S]Foni yechipiri yaunokumbidzana nevamwe inonzii?	[Q212S]What is the make of the second phone you share?
[Q212S]Model yefoni yechipiri chii?	[Q212S]What is the model of the second phone you share?
[Q212S] Foni yechipiri iyi smart phone here?	[Q212S] Is the second phone you share a smart phone?
[Q212S] Foni yechipiri iyi wakaitenga rinhi?	[Q212S]Which year was the second phone you share purchased?
[Q213S] Unoshandisa kana kukumbidzana cell phone yechipiri nani?	[Q213S] Who do you share ownership of your second phone with?
[Q213S] Tsanangudza vamwe vanhu vaunoshandisa phone navo	[Q213C] Specify other
[Q213S] Kakawanda zvakadii kamunowana mukana wekushandisa phone yechipiri?	[Q232S] How often do you have access to the second phone you share?
**Phone Yechitatu Yaanokumbidzana kana kushandisa nevamwe**	**Phone 3 Shared**
[Q212S]Foni yechitatu yaunokumbidzana nevamwe inonzii?	[Q212S]What is the make of the third phone you share?
[Q212S]Model yefoni yechitatu chii?	[Q212S]What is the model of the third phone you share?
[Q212S] Foni yechitatu iyi smart phone here?	[Q212S]Is the third phone you share a smart phone?
[Q212S] Foni yechitatu iyi wakaitenga rinhi?	[Q212S]Which year was the third phone you share purchased?
[Q213S] Unoshandisa kana kukumbidzana cell phone yechitatu nani?	[Q213S] Who do you share ownership of your third phone with?
[Q213S] Tsanangudza vamwe vanhu vaunoshandisa phone navo	[Q213C] Specify other
[Q213S] Kakawanda zvakadii kamunowana mukana wekushandisa phone yechitatu?	[Q232S] How often do you have access to the third phone you share?
**ZVEVANE PHONE KANA KUWANA MUKANA WE PHONE**	**FOR THOSE WITH PHONES OR ACCESS**
**MHANDO DZEMALINE DZINOSHANDISWA**	**MOBILE PROVIDERS USED**
[Q214prov] Unowanzoshandisa line ripi?	[Q214prov] Which mobile provider do you use normally?
[Q215]Unoshandisawo futi Econet	[Q215] Also use Econet network
[Q215] Unoshandisawo futi Telecel	[Q215] Also use Telecel network
[Q215] Unoshandisawo futi Netone	[Q215] Also use Netone network
[Q215]Unoshandisawo futi Africom	[Q215] Also use Africom network
**MARI DZINOSHANDISWA PA MOBILE PHONE**	**EXPENDITURE ON MOBILE PHONE**

For peer review only

[Q216air] Mari yekufonesa pa cell phone inogarotengwa here kana kuti inotengwa pamwedzi pamwedzi (contract)?	[Q216 air]For your phone, does airtime need to be purchased in advance or is it paid monthly through a contract?
[Q217amt] Pasvondo rapfuura, wakashandisa mari inosvika madhora mangani pa airtime?	[Q217amt] How much airtime, in dollars, did you use in the past week?
[Q218exp] Pasvondo rapfuura, wakashandisa mari inosvika madhora mangani pane zvimwe zvinhu zvenyu tisingasanganise ne airtime?	[Q218exp] How much money did you spend on other things for yourself in the past week excluding airtime for cell phones?
**NDIANI ANOWANZOBHADHARA MARI DZINOSHANDISWA PA MOBILE PHONE**	**WHO USUALLY PAYS FOR EXPENDITURE ON MOBILE PHONE**
[Q219]Ndini	[Q219]I do
[Q219]Vabereki/ Vanondichengeta	[Q219]My parents/ legal guardians
[Q219]Dzimwe Hama	[Q219]Other relative
[Q219]Shamwari	[Q219]Friend
[Q219]Mudiwa diwa	[Q219]Boyfriend/girlfriend
[Q219]Handishandise mari paairtime	[Q219]I don't spend money on airtime
[Q219]Vamwewo (Tsanangudza pazasi)	[Q219]Other (specify below)
[Q219_97]Handizivi	[Q219_97]Don't know
[Q219_99]Hapana	[Q219_99]No response
[Q219_other] Tsanangudza vamwe vano badhara	[Q219_other] specify Other person who pays for credit
[Q220_age] Wakatanga kushandisa cell phone une makore mangani?	[Q220_age] At what age did you start using a mobile phone?
**MIBVUNZO YEVANE PHONE CHETE**	**ONLY FOR THOSE WHO OWN A PHONE**
[Q221]Wakawana cell phone yako yekutanga sei?	[Q221] How did you get your first phone?
[Q221] Tsanangudza zvimwe zvakaita kuti uve nephone yekutanga	[Q221] Specify other reason why you got your first phone
[Q222] Akatenga cell phone yako yekutanga ndiyani?	[Q222]Who bought your first phone?
[Q222] Tsanangura mumwe akatenga phone	[Q222] Specify other person who bought first phone
**Inguva dzipi dzinowanzo kunge dzakadzima cell phone yako?**	**When is your phone usually switched off**
[Q223]Hapana pandinodzima	[Q223] Never
[Q223]Pandinenge ndakavata	[Q223]When I am sleeping
[Q223]Pandinenge ndiri kuchikoro/kubasa	[Q223]When I am at school/ work
[Q223]Kana ndisisina mari yekufonesa	[Q223]When I have run out of credit
[Q223]Kana bhatiri rava nemoto mushoma	[Q223]When the battery is low
[Q223]Dzimwewo nguva (tsanangurai pazasi)	[Q223]Other (specify below)
[Q223]Handizivi	[Q223]Don't know
[Q223]Hapana minduro	[Q223]No response
[Q223] Tsanangurai dzimwe nguva dzinodzimwa phone	[Q223]Specify other time phone is switched off
[Q224] Kakawanda zvakadii kamunoita kuti cell phone yenyu isarire zvinonzwika ('SILENT' or 'VIBRATE')?	[Q224] How often do you place your phone on 'SILENT' or 'VIBRATE' mode?
[Q225]Unotakura cell phone yako kuchikoro here?	[Q225] Do you take your cell phone to school ?
[Q226] Unogona kugara usina cell phone kwezuva rese here?	[Q226] Can you do without a mobile for a day?
[Q227] Kana ati kwete, nemhaka yei?	[Q227] Why cannot you not do without a phone
[Q228] Kwamuri chii chakanakira kuita cell phone?	[Q228] According to you, the best part of having a mobile phone is
[Q228] Tsanangurai zvimwe zvakanakira kuita cell phone?	[Q228] What are the other best part of having a phone
**Vasina Phone**	**For those with No Phone**
[Q229] Kana usina cell phone, nemhaka yei musina?	[Q229] If you don't have a mobile phone, kindly tell me why not?

For peer review only

[Q229] Zwimwe zvinoita kuti musave ne phone	[Q229] Whats the other reason for not having a phone
[Q235] Mune hurongwa here hwekutenga cell phone munguva inotevera?	[Q235] Are you planning to buy a mobile phone in near future?
[Q236] Kana mati hongu, riinhi?	[Q236] When are you planning to buy a mobile phone?
**Unoshandisa cell phone zvakadini kana uchiita izvi:**	**How frequently do you use a mobile phone for:**
[Q237]Kufona	[Q237]Phone calls
[Q237]Kutumira ma message kana pictures (kwete ne app)	[Q237]Sending text or picture messages (not via an app)
[Q237]Kutumira ma message kana chat (pa Whatsapp, Facebook)	[Q237]Sending instant messages or chat services (e.g Whatsapp, Facebook etc.)
[Q237]Kutamba ma games	[Q237]Playing games
[Q237]Kuona TV/movies/videos	[Q237]Watch TV/movies/videos
[Q237Kuterera kana ku downloader music/radio	[Q237]Listen to/ download music/radio
[Q237]Kunzwa news dzemitambo kana zvimwe	[Q237]Getting news updates (sports or other)
[Q237]Calculator	[Q237]Calculator
[Q237]]Social network yakafanana ne twitter, Instagram, Facebook	[Q237]]Social networking sites e.g. twitter, Instagram, Facebook
[Q237]Kubhadhara kana kutengesa zvinhu	[Q237]Making payments/ purchasing goods
**Unoshandisa cell phone zvakadini kana uchiita izvi:**	**How frequently do you use a mobile phone for:**
[Q237]Camera	[Q237]Camera
[Q237]Kufamba kwako nema maps	[Q237]Navigating with maps
[Q237]E-mailing	[Q237]E-mailing
[Q237]Uchitsvaga ruzivo rwezvekuchikoro uchiita homework kana zvekubasa	[Q237]Research for school/work or doing homework /revision e.g. Ruzivo
[Q237]Uchitsvaga ruzivo rwezveutano	[Q237]Search for information about my health
[Q237]Uchitsvaga ruzivo rwezvekudanana ne zvepabonde	[Q237]Search for information about relationships and sex
[Q237]Calendar	[Q237]Calendar
[Q237]Clock	[Q237]Clock
[Q237]Dictionary	[Q237]Dictionary
[Q237]Bible	[Q237]Bible
**Ndeapi ma apps matatu aunowanzoshandisa?**	**What are the three apps that you most often use ?**
[Q238_1]Sarudza app yokutanga	[Q238_1]Select the first app
[Q238_1]Tsanagudza rimwe zita	[Q238_1]Specify other
[Q238_2] Sarudza app yechipiri	[Q238_2]Select the second app
[Q238_2]Tsanagudza rimwe zita	[Q238_2]Specify other
[Q238_3]Sarudza app yechitatu	[Q238_3]Select the third app
[Q238_3]Tsanagudza rimwe zita	[Q238_3]Specify other
[Q239] Unombotamba ma games pa cell phone?	[Q239] Do you play any games on a cell phone?
**Ndeapi ma games matatu aunowanzotamba pa cell phone?**	**What are the three games you most often play on a cell phone?**
[Q239] Zita re Game rokutanga	[Q239] Name Game 1
[Q239] Tsanangudza Zita re Game rokutanga	[Q239] Specify Name Game 1
[Q239] Zita re Game rechipiri	[Q239] Name Game 2
[Q239] Tsanangudza Zita re Game rechipiri	[Q239] Specify Name Game 2
[Q239] Zita re Game rechitatu	[Q239] Name Game 3
[Q239] Tsanangudza Zita re Game rechitatu	[Q239] Specify Name Game 3
[Q240]Wakamboshandisa phone kuti uone kuti utano hwako hurikufamba sei?	[Q240] Have you ever used your phone for tracking your health?
[Q241]Nditaurire ma apps aunoshandisa kuona kuti utano hwako hwakamira sei	[Q241]Please list the applications that you use to track your personal health measures

For peer review only

ZVAKAVANZIKA kana PRIVATE Dzako	PRIVACY
[Q242]Unofunga kuti zvaunotumira paphone zviri 'private' kana zvakavanzika here?	[Q242]How 'private' do you consider the information that you send when using a phone?
[Q243]Unofunga kuti zvaunotumirwa paphone zviri 'private' kana zvakavanzika here?	[Q243]How 'private' do you consider the information that you receive when using a phone?
[Q244]Unofunga kuti zviri paphone zviri 'private' kana zvakavanzika here?	[Q244]How 'private' do you consider the information stored in your mobile phone?
[Q245] Une mapasswords yeku Locker phone yako here?	[Q245] Do you have passwords to lock/unlock your phone?
[Q246]Mune mapasswords eApplications ipi zvayo pacell phone yako?	[Q246]Do you have passwords for any applications on your phone?
[Q246b]Kana wati hongu, ndeapi maApplications anema passwords??	[Q246b]If yes, which ones?
**SECTION 3 PFUNGWA DZAKO - SSQ**	**SECTION 3 MENTAL HEALTH SSQ**
Iye zvinondichakubvunzai kuti mainzwa sei muvhiki rapfuura. Ndapota pindurai hongu kana kwete pamubvunzo umwe neumwe	Now I am going to ask you how you have been feeling over the past week. Please respond yes or no to each question
Muvhiki rapfuura	**In the past week**
[Q301]Pane pandaimboona ndichinyanya kufungisisa kana kufunga zvakawanda	[Q301]There were times in which I was thinking deeply or thinking about many things
[Q302]Pane pandaimbotadza kuisa pfungwa dzangu pamwechete	[Q302]I found myself sometimes failing to concentrate
[Q303]Pane pandaimboshatirwa kana kuita hasha zvenhando	[Q303]I lost my temper or got annoyed over trivial matters
[Q304]Pane pandaimborota hope dzinotyisa kana dzisina kunaka	[Q304]I had nightmares or bad dreams
[Q305]Pane pandaimboona kana kunzwa zvinhu zvaisaonekwa kana kunzwikwa nevamwe	[Q305]I sometimes saw or heard things which others could not see or hear
[Q306]Pane pandaimborwadziwa nemudumbu	[Q306]My stomach was aching
Muvhiki rapfuura	**In the past week**
[Q307]Pane pandaimbovhundutswa nezvinhu zvisina mature	[Q307]I was frightened by trivial things
[Q308]Pane pandaimbotadza kurara kana kushaya hope	[Q308]I sometimes failed to sleep or lost sleep
[Q309]Pane pandaimbonzwa kuomerwa neupenyu zvekuti ndaimbochema kana kunzwa kuda kuchema	[Q309]There were moments when I felt life was so tough that I cried or wanted to cry
[Q310]Pane pandaimbonzwa kuneta (kurukutika)	[Q310]I felt run down (tired)
[Q311]Pane pandaimboita pfungwa dzekuda kuzviuraya	[Q311]At times I felt like committing suicide
[Q312]Ndainzwa kusafara nezvinhu zvandaiita zuva nezuva	[Q312]I was generally unhappy with things that I would be doing each day
[Q313]Basa rangu rava kurasarira mumashure	[Q313]My work was lagging behind
[Q314]Ndainzwa zvichindiomera kuti ndoita zvipi	[Q314]I felt I had problems in deciding what to do
**PFUNGWA DZAKO - SDQ**	**MENTAL HEALTH AND WELL-BEING SDQ**
**Ndinokumbira kuti upe mhinduro dzako wakatarisa zvaiitika kana mamiriro epfungwa dzako mwedzi mitanhatu yapfuura.**	Please give your answers based on how things have been for you over the last six months.
**MUMWEDZI MITANHATU YAPFUURA NDAINZWA KANA KUFUNGA IZVI**	**MENTAL HEALTH AND WELL-BEING SDQ in the last six months**
[Q401]Ndinoyedza kuve munhu akanaka kune vamwe vanhu. Ndinoda kuita zvinoita kuti vafare	[Q401]I try to be nice to other people. I care about their feelings.
[Q402]Handigadzikane, handigoni kugara panzvimbo nguva yakareba	[Q402]I am restless, I cannot stay still for long
[Q403]Ndinogara ndichinzwa musoro, mudumbu nekungorwara rwara	[Q403]I get a lot of headaches, stomach-aches or sickness

For peer review only

[Q404]Nguva zhinji ndinogovana nevamwe(zvekudya, zvekutambisa, zvinyoreso nezvimwe)	[Q404]I usually share with others (food, games, pens etc.)
[Q405]Ndinotsamwa zvekutsamwa zviya zvakare ndinokurumidza kushatirwa	[Q405]I get very angry and often lose my temper
[Q406]Nguva zhinji ndinenge ndiri ndega. Ndinotamba ndega kana kugara ndega	[Q406]I am usually on my own. I generally play alone or keep to myself
[Q407]Ndinowanzoita zvandinenge ndaudzwa	[Q407]I usually do as I am told
[Q408]Ndinofunganya zvakanyanya	[Q408]I worry a lot
**MUMWEDZI MITANHATU YAPFUURA NDAINZWA KANA KUFUNGA IZVI**	**MENTAL HEALTH AND WELL-BEING SDQ in the last six months**
[Q409]Ndinobatsira kana mumwe munhu arwadziswa kana asiri kunzwa zvakanaka kana ashatiriswa	[Q409]I am helpful if someone is hurt, upset or feeling ill
[Q410]Ndinogara ndichingoshanyarika ndisingagadzikane	[Q410]I am constantly fidgeting or squirming
[Q411]Ndine shamwari imwechete kana dzakawanda	[Q411]I have one good friend or more
[Q412]Ndinorwa zvakanyanya .Ndinogona kuti vamwe vanhu vaite zvandinoda	[Q412]I fight a lot. I can make other people do what I want.
[Q413]Nguva zhinji ndinenge ndakasuwa, moyo wangu uchirwadza kana kuda kuchema	[Q413]I am often unhappy, down-hearted or tearful
[Q414]Vamwe vanhu vezera rangu vanondifarira	[Q414]Other people my age generally like me
[Q415]Ndinokurumidza kuita charinga, ndinotadza kunyatsoita sezvandinotarisirwa	[Q415]I am easily distracted, I find it difficult to concentrate
[Q416]Ndinonzwa kutya munzvimbo itsva. Ndinokurumidza kushaya chivimbo nezvandinenge ndichiita	[Q416]I am nervous in new situations. I easily lose confidence.
[Q417]Ndine tsitsi kuvanhu vechidiki	[Q417]I am kind to younger children.
[Q418]Ndinogara ndichipomerwa kunyepa nekusataura chokwadi	[Q418]I am often accused of lying or cheating
**MUMWEDZI MITANHATU YAPFUURA NDAINZWA KANA KUFUNGA IZVI**	**MENTAL HEALTH AND WELL-BEING SDQ in the last six months**
[Q419]Vamwe vana vanondisvora vachindichisidzira nekundirova kana kundituka kana kungoita zvinondidzikisira	[Q419]Other children or young people pick on me or bully me
[Q420]Kazhinji ndinobatsira vamwe(vabereki, vadzidzisi nevana)	[Q420]I often volunteer to help others (parents, teachers, children)
[Q421]Ndinonyatsofunga ndisati ndaita zvinhu	[Q421]I think before I do things
[Q422]Ndinotora zvinhu zvisiri zvangu kubva kumba, kuchikoro kana kumwewo	[Q422]I take things that are not mine from home, school or elsewhere
[Q423]Ndinowirirana zvakanaka nevanhu vakuru panevezera rangu	[Q423]I get on better with adults than with people my own age
[Q424]Ndine zvandinotya, ndinokurumidza kuvhunduka/kutya	[Q424]I have many fears, I am easily scared
[Q425]Ndinopedza basa randinenge ndichiita. Ndinonyatsoita sezvinotarisirwa	[Q425]I finish the work I’m doing. My attention is good

For peer review only

Table S1: Shona symptom questionnaire (SSQ): item response rates by questionnaire version used (n=634)

	**Total** [a]	**Version 1**	**Version 2**	**p-value for age-adjusted difference between versions** [b]
n	634	123	511	
Median SSQ (IQR)	7 (4, 9)	7 (4,9)	7 (4,9)	
Mean SSQ (95% CI)	6.44 (6.13, 6.74)	6.36 (5.60, 7.12)	6.46 (6.13, 6.79)	p=0.826
At risk for CMD (SSQ>=8)	37.38 (32.99, 41.99)	41.46 (32.40, 51.14)	36.40 (31.48, 41.62)	p=0.300
At risk for CMD (SSQ>=9)	28.55 (24.88, 32.53)	31.71 (24.33, 40.14)	27.79 (23.66, 32.33)	p=0.346
Severely at risk of CMD (SSQ>=11)	11.51 (9.08, 14.51)	13.82 (8.10, 22.59)	10.96 (8.42, 14.15)	p=0.410
I sometimes saw or heard things which others could not see or hear	9.78 (7.47, 12.71)	12.20 (7.07, 20.24)	9.20 (6.78, 12.36)	p=0.369
At times I felt like committing suicide	11.20 (9.01, 13.84)	14.63 (9.13, 22.64)	10.37 (8.10, 13.20)	p=0.166
Red flag	22.08 (18.55, 26.07)	27.64 (19.02, 38.32)	20.74 (17.04, 25.02)	p=0.160

a. mean and proportions taking into account clustered study design but not adjusted for age
b. Adjusted Wald test accounting for clustered study design and adjusting for age in years

Table S2 SSQ – item response rates by sex and overall response (yes, no) (n=634)

**Shona Symptom Questionnaire items**		**Males**		**Females**		**Total**
	N		N		N	
There were times in which I was thinking deeply or thinking about many things	153	64.56%	253	63.73%	406	64.04%
I found myself sometimes failing to concentrate	145	61.18%	225	56.68%	370	58.36%
I lost my temper or got annoyed over trivial matters	129	54.4%	237	59.7%	366	57.7%
I had nightmares or bad dreams	143	60.3%	257	64.7%	400	63.1%
I sometimes saw or heard things which others could not see or hear	34	14.4%	28	7.1%	62	**9.8%**
My stomach was aching	146	61.6%	273	68.8%	419	66.1%
I was frightened by trivial things	88	37.1%	150	37.8%	238	37.5%
I sometimes failed to sleep or lost sleep	119	50.2%	211	53.2%	330	52.1%

For peer review only

There were moments when I felt life was so tough that I cried or wanted to cry	116	49.0%	227	57.2%	343	54.1%
I felt run down (tired)	127	53.6%	242	61.0%	369	58.2%
At times I felt like committing suicide	17	7.2%	54	13.6%	71	**11.2%**
I was generally unhappy with things that I would be doing each day	91	38.4%	110	27.7%	201	31.7%
My work was lagging behind	85	35.9%	97	24.4%	182	28.7%
I felt I had problems in deciding what to do	117	49.4%	207	52.1%	324	51.1%

For peer review only

Table S3: Strength and Difficulties Questionnaire (SDQ): item response rates by sex and overall response (n=634)

**Mean SDQ scores**	All (n=634)			Males (n=237)			Females (n=397)		
	**Mean**	S.D.	Alpha	**Mean**	S.D.	Alpha	**Mean**	S.D.	Alpha
Difficulties total	**10.74**	6.25	0.75	**10.72**	6.23	0.74	**10.75**	6.28	0.75
Externalising score	**4.31**	3.48	0.68	**4.65**	3.59	0.67	**4.10**	3.41	0.69
Internalising score	**6.43**	3.94	0.63	**6.07**	3.69	0.59	**6.64**	4.08	0.64
Emotional symptoms	**3.66**	2.66	0.60	**3.38**	2.56	0.59	**3.83**	2.71	0.61
Conduct problems	**2.22**	1.97	0.46	**2.24**	2.09	0.49	**2.20**	1.89	0.46
Hyperactivity/inattention	**2.09**	2.03	0.52	**2.41**	2.07	0.50	**1.90**	1.99	0.54
Peer problems	**2.77**	2.07	0.38	**2.69**	1.94	0.33	**2.81**	2.15	0.41
Prosocial behaviour	**8.13**	2.22	0.68	**8.14**	2.24	0.69	**8.12**	2.20	0.67

Range of all scales 0-10, except Difficulties Total which ranges 0-40

For peer review only

Table S4 Mean SDQ scores by sex with adjustment for age and cluster sampling design

Mean SDQ scores	All (n=634)		Males (n=237)		Females (n=397)		Difference Wald		
	Mean	95% CI	Mean	95% CI	Mean	95% CI	F	d.f.	P
Difficulties total	**10.74**	6.25	**10.71**	9.84 -11.58	**10.75**	10.12-11.38	0.01	1,123	0.9379
Externalising score	**4.31**	3.48	**4.63**	4.14-5.13	**4.11**	3.79-4.44	3.86	1,123	0.0517
Internalising score	**6.43**	3.94	**6.08**	5.58-6.57	**6.64**	6.23-7.05	3.02	1,123	0.0848

For peer review only

STROBE 2007 (v4) Statement—Checklist of items that should be included in reports of *cross-sectional studies*

Section/Topic	Item #	Recommendation	Reported on page #
**Title and abstract**	1	(*a*) Indicate the study's design with a commonly used term in the title or the abstract	3
		(*b*) Provide in the abstract an informative and balanced summary of what was done and what was found	3
**Introduction**			
Background/rationale	2	Explain the scientific background and rationale for the investigation being reported	4
Objectives	3	State specific objectives, including any prespecified hypotheses	4
**Methods**			
Study design	4	Present key elements of study design early in the paper	4
Setting	5	Describe the setting, locations, and relevant dates, including periods of recruitment, exposure, follow-up, and data collection	4
Participants	6	(*a*) Give the eligibility criteria, and the sources and methods of selection of participants	4
Variables	7	Clearly define all outcomes, exposures, predictors, potential confounders, and effect modifiers. Give diagnostic criteria, if applicable	5
Data sources/ measurement	8*	For each variable of interest, give sources of data and details of methods of assessment (measurement). Describe comparability of assessment methods if there is more than one group	5
Bias	9	Describe any efforts to address potential sources of bias	*5*
Study size	10	Explain how the study size was arrived at	5
Quantitative variables	11	Explain how quantitative variables were handled in the analyses. If applicable, describe which groupings were chosen and why	5-6
Statistical methods	12	(*a*) Describe all statistical methods, including those used to control for confounding	5-6
		(*b*) Describe any methods used to examine subgroups and interactions	
		(*c*) Explain how missing data were addressed	
		(*d*) If applicable, describe analytical methods taking account of sampling strategy	
		(*e*) Describe any sensitivity analyses	
**Results**			

For peer review only

Participants	13*	(a) Report numbers of individuals at each stage of study—eg numbers potentially eligible, examined for eligibility, confirmed eligible, included in the study, completing follow-up, and analysed	7
		(b) Give reasons for non-participation at each stage	7
		(c) Consider use of a flow diagram	Figure 1
Descriptive data	14*	(a) Give characteristics of study participants (eg demographic, clinical, social) and information on exposures and potential confounders	7
		(b) Indicate number of participants with missing data for each variable of interest	7
Outcome data	15*	Report numbers of outcome events or summary measures	7-9
Main results	16	(*a*) Give unadjusted estimates and, if applicable, confounder-adjusted estimates and their precision (eg, 95% confidence interval). Make clear which confounders were adjusted for and why they were included	7-14 and Tables 1-4
		(*b*) Report category boundaries when continuous variables were categorized	
		(*c*) If relevant, consider translating estimates of relative risk into absolute risk for a meaningful time period	
Other analyses	17	Report other analyses done—eg analyses of subgroups and interactions, and sensitivity analyses	Supplementary material
**Discussion**			
Key results	18	Summarise key results with reference to study objectives	14
Limitations	19	Discuss limitations of the study, taking into account sources of potential bias or imprecision. Discuss both direction and magnitude of any potential bias	16
Interpretation	20	Give a cautious overall interpretation of results considering objectives, limitations, multiplicity of analyses, results from similar studies, and other relevant evidence	15-16
Generalisability	21	Discuss the generalisability (external validity) of the study results	15
**Other information**			
Funding	22	Give the source of funding and the role of the funders for the present study and, if applicable, for the original study on which the present article is based	17

*Give information separately for cases and controls in case-control studies and, if applicable, for exposed and unexposed groups in cohort and cross-sectional studies.

Note: An Explanation and Elaboration article discusses each checklist item and gives methodological background and published examples of transparent reporting. The STROBE checklist is best used in conjunction with this article (freely available on the Web sites of PLoS Medicine at http://www.plosmedicine.org/, Annals of Internal Medicine at http://www.annals.org/, and Epidemiology at http://www.epidem.com/). Information on the STROBE Initiative is available at www.strobe-statement.org.

For peer review only
